# A Bacterial Homolog of a Eukaryotic Inositol Phosphate Signaling Enzyme Mediates Cross-kingdom Dialog in the Mammalian Gut

**DOI:** 10.1016/j.celrep.2014.01.021

**Published:** 2014-02-13

**Authors:** Régis Stentz, Samantha Osborne, Nikki Horn, Arthur W.H. Li, Isabelle Hautefort, Roy Bongaerts, Marine Rouyer, Paul Bailey, Stephen B. Shears, Andrew M. Hemmings, Charles A. Brearley, Simon R. Carding

**Affiliations:** 1 Gut Health and Food Safety Programme, Institute of Food Research, Norwich NR4 7UA, UK; 2 School of Biological Sciences, University of East Anglia, Norwich NR4 7TJ, UK; 3 School of Chemistry, University of East Anglia, Norwich NR4 7TJ, UK; 4 Norwich Medical School, University of East Anglia, Norwich NR4 7TJ, UK; 5 Department of Computational and Systems Biology, John Innes Centre, Norwich NR4 7UH, UK; 6 Laboratory of Signal Transduction, National Institute of Environmental Health Sciences, Research Triangle Park, NC 27709, USA

## Abstract

Dietary InsP_6_ can modulate eukaryotic cell proliferation and has complex nutritive consequences, but its metabolism in the mammalian gastrointestinal tract is poorly understood. Therefore, we performed phylogenetic analyses of the gastrointestinal microbiome in order to search for candidate InsP_6_ phosphatases. We determined that prominent gut bacteria express homologs of the mammalian InsP_6_ phosphatase (MINPP) and characterized the enzyme from *Bacteroides thetaiotaomicron* (BtMinpp). We show that BtMinpp has exceptionally high catalytic activity, which we rationalize on the basis of mutagenesis studies and by determining its crystal structure at 1.9 Å resolution. We demonstrate that BtMinpp is packaged inside outer membrane vesicles (OMVs) protecting the enzyme from degradation by gastrointestinal proteases. Moreover, we uncover an example of cross-kingdom cell-to-cell signaling, showing that the BtMinpp-OMVs interact with intestinal epithelial cells to promote intracellular Ca^2+^ signaling. Our characterization of BtMinpp offers several directions for understanding how the microbiome serves human gastrointestinal physiology.

## Highlights

• *Bacteroides thetaiotaomicron* (Bt) secretes a cell-signaling InsP6 phosphatase MINPP
• BtMinpp is exceptionally active and rationalized from its crystal structure
• BtMinpp is secreted in outermembrane vesicles
• BtMinpp/OMVs promote Ca^2+^ signaling in intestinal epithelial cells

## Graphical Abstract

**Figure:**
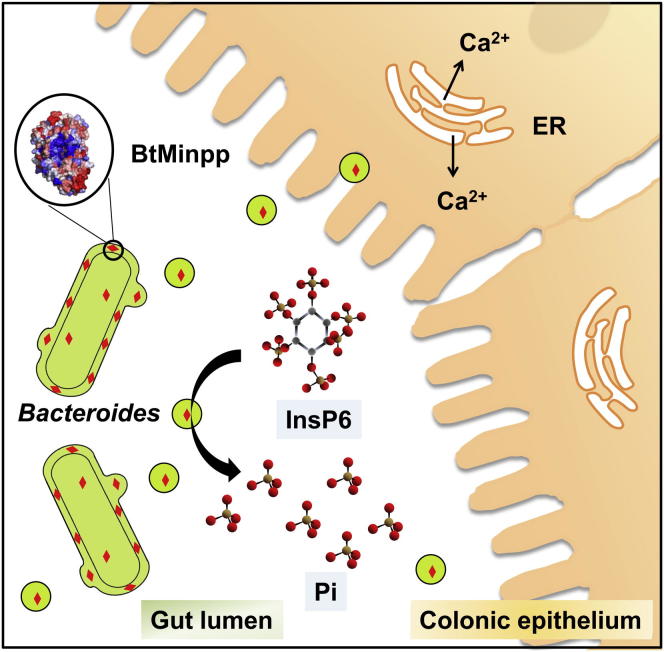

## Teaser

Brearley, Carding, and colleagues now find that the prevalent gut bacterium *Bacteroides thetaiotaomicron* (Bt) contributes to human physiology, metabolism, and homeostasis through gastrointestinal InsP_6_ metabolism and secretion of a mammalian cell-signaling InsP_6_ phosphatase, MINPP. BtMinpp catalytic activity was exceptionally high and rationalized from its crystal structure. BtMinpp is packaged inside outermembrane vesicles (OMVs) protecting against intestinal proteases. BtMinpp and OMVs also promoted Ca^2+^ signaling in intestinal epithelial cells, an example of cross-kingdom dialog.

## Introduction

The adult human gastrointestinal (GI) tract accommodates a bacterial community that comprises trillions of cells. This microbiota has many essential roles in human health (Tremaroli and Bäckhed, 2012): it suppresses proliferation of pathogenic microbes and has important nutritional consequences, including vitamin synthesis and fermentation of complex dietary carbohydrates. Microbial metabolites also regulate the signaling activities of the host’s intestinal epithelium, which, for example, aids the development and maintenance of local immune responses. Thus, considerable efforts are now being made to determine the precise nature of the dialog between gut bacteria and the human host. Much of the previous work in this area has focused on the roles of diffusible, small-molecule hormones and nutrients. We now describe a vehicle produced by a prevalent gut bacterium, *Bacteroides thetaiotaomicron* (Bt): a vesicle-enclosed homolog of a mammalian cell-signaling InsP_6_ phosphatase, MINPP.

Enzymatic homeostasis of InsP_6_ levels in the gut can have far-reaching consequences for human health. Considerable quantities of InsP_6_ are ingested daily as it is the primary storage form of phosphorus in cereals and legumes (Kumar et al., 2010). InsP_6_ is therefore a source of inositol and phosphate, two vital nutrients. However, InsP_6_ is also considered to have antinutritive properties, given that it inhibits polysaccharide digestibility and chelates divalent cations, thereby limiting their bioavailability in the GI tract (Kumar et al., 2010). More recently, with the emergence of cell-signaling activities for InsP_6_ and other members of the inositol phosphate family, dietary InsP_6_ has received separate attention for its anticarcinogenic properties (Fox and Eberl, 2002, Vucenik and Shamsuddin, 2003), at least when present at high concentrations. Conversely, lower concentrations of InsP_6_ may stimulate tumor cell proliferation (Windhorst et al., 2013). In any case, exogenous InsP_6_ can enter cells and be metabolized (Windhorst et al., 2013), thereby potentially contributing to intracellular signaling processes in intestinal epithelial and immune cells. For example, InsP_6_ is the precursor for inositol pyrophosphates, which have pleiotropic functions including the regulation of energy metabolism (Szijgyarto et al., 2011), insulin sensitivity (Chakraborty et al., 2010), and bactericidal activities of immune cells (Prasad et al., 2011).

Humans and other monogastric animals lack enzymes capable of participating in dietary InsP_6_ homeostasis, relying instead on exogenous phosphatases provided by resident microbes (Haros et al., 2007, Steer et al., 2004). Surprisingly, the nature of the genes that encode these phosphatases is unknown for virtually the entire microbiome of the human GI tract, although they are generally assumed to be “phytases” (Haefner et al., 2005, Steer et al., 2004, Tamayo-Ramos et al., 2012). Such enzymes occur in a number of environmental niches in which they scavenge inorganic phosphate from InsP_6_, but they have no signaling function. Moreover, if phytases were to be secreted into the GI tract, it is unclear how they might access dietary InsP_6_ while avoiding digestive proteases. In the current study, our phylogenetic, biochemical, and structural characterization of BtMinpp characterizes an unexpected repertoire of widely distributed and highly active class of InsP_6_ phosphatases in the GI tract.

## Results

### *B. thetaiotaomicron* Express a Homolog of a Mammalian Inositol Phosphate Signaling Phosphatase

The Bacteroidetes are a dominant constituent of the mammalian GI tract microbiota. The genome of *B. thetaiotaomicron* (Xu et al., 2003) contains a gene, *BT_4744*, which has a primary annotation (Hidden Markov Models-based annotation) as encoding a putative multiple inositol-polyphosphate phosphatase (MINPP, EC 3.1.3.62). This is a notable observation because bacteria have not previously been suggested to utilize the inositol phosphate signaling cascade (Michell, 2008). Indeed, MINPPs have only previously been considered to function inside animal cells, mainly by regulating levels of InsP_5_ and InsP_6_ (Chi et al., 2000, Romano et al., 1998). Strikingly, BT_4744 shares no significant sequence similarity with the bacterial or fungal phytases described in the literature (see Table S1). We shall refer to the *BT_4744*-encoded enzyme as BtMinpp, and below we further justify this characterization.

**Figure 1:**
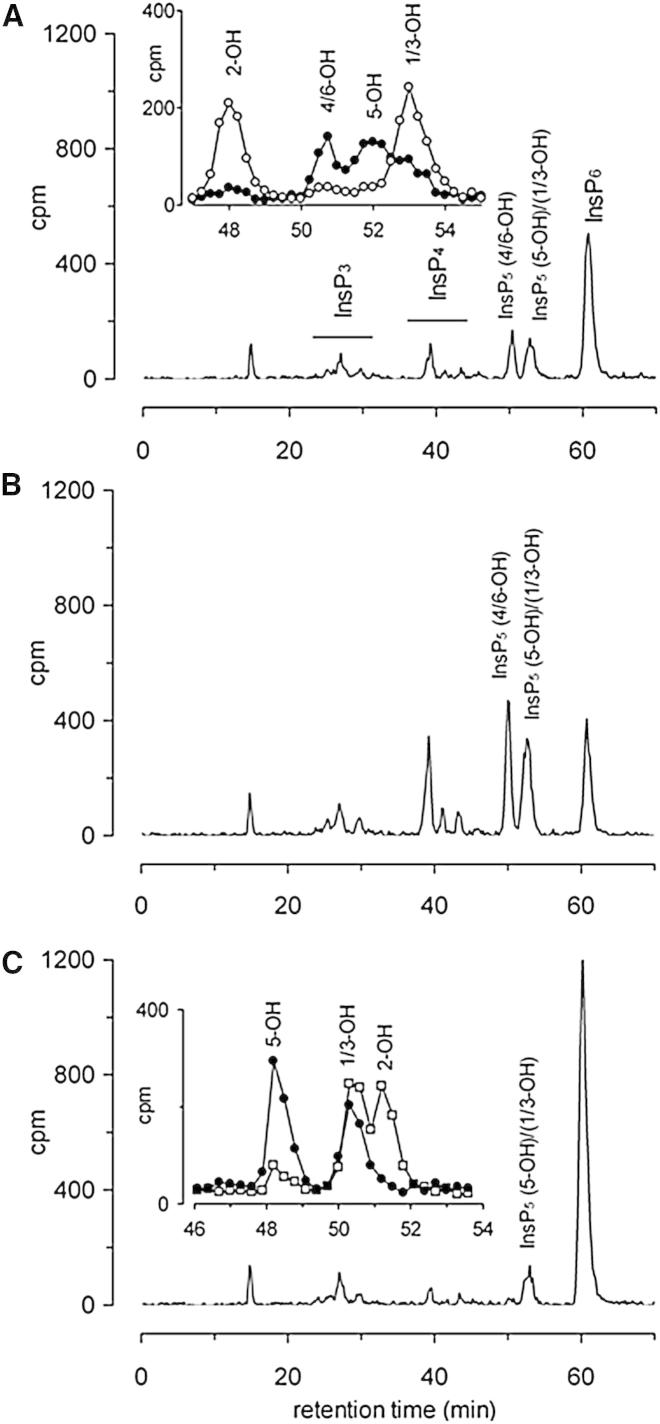
Mutation of Active Site Residues Alters the Specificity of Initial Attack on InsP_6_ Substrate (A–C) Products of reaction of native BtMinpp- (A), A31Y- (B), and R183D- (C) substituted enzyme with *myo*-inositol(1,[^32^P]2,3,4,5,6)P_6_ were resolved by Partisphere SAX HPLC. Mutated proteins were incubated at a concentration of 2.5 μg/ml and native protein at 0.25 μg/ml, with 1 mM InsP_6_. The regions of the chromatogram in which InsP_3_, InsP_4_, and specific InsP_5_ isomers elute are indicated in (A). For native enzyme (inset in A), reaction products were mixed with standards of *myo*-[2-^3^H]inositol (1,3,4,5,6)P_5_ (InsP_5_ [2-OH]), D/L- *myo*-[2-^3^H]inositol (1,2,4,5,6)P_5_ (InsP_5_ [1/3-OH]), and D/L- *myo*-[2-^3^H]inositol (1,2,3,4,5)P_5_ (InsP_5_ [4/6-OH]). Fractions, 0.25 min, were collected and radioactivity was estimated by scintillation counting; ^3^H, open circles; ^32^P, filled circles. For R183D-substituted enzyme (inset in C), the reaction products were additionally mixed with standards of *myo*-[^14^C]InsP_5_ [2-OH] and D/L- *myo*-[^14^C]InsP_5_ [1/3-OH] and resolved on a Adsorbosphere SAX HPLC column. This column separates InsP_5_ [2-OH] from InsP_5_ [1/3-OH]. Fractions, 0.25 min, were collected and radioactivity estimated by scintillation counting: ^14^C, open squares; ^32^P, filled circles.

High-performance liquid chromatography (HPLC) analysis of the catalytic activity of a His-tagged recombinant form of BtMinpp confirmed that this protein has InsP_6_ phosphatase activity (Figure 1A). The V_max_ value for InsP_6_ (178 μmol/mg/min; Table S2) is the highest yet reported for any member of the MINPP family, being several orders of magnitude greater than that for the human (0.006 μmol/mg/min) and avian (0.7 μmol/mg/min) representatives (Ali et al., 1995, Cho et al., 2006). We incubated BtMinpp with Ins(1,[^32^P]2,3,4,5,6)P_6_ substrate and identified the InsP_5_ products from their HPLC elution positions relative to *myo*-[2-^3^H]InsP_5_ standards (Figure 1A, inset). Although Ins(1,3,4,5,6)P_5_ (InsP_5_ [2-OH]) cannot be detected using Ins(1,[^32^P]2,3,4,5,6)P_6_ as substrate, we can be confident that InsP_5_ [2-OH] is not a major product because of the retention of radiolabel in progressively less-phosphorylated products, InsP_4_, InsP_3_, and InsP_2_ (Figure S1C). In any case, the use of Ins(1,[^32^P]2,3,4,5,6)P_6_ as substrate revealed that multiple InsP_5_ products were formed by BtMinpp, including D-and/or L-Ins(1,2,3,4,5)P_5_ (InsP_5_ [4/6-OH]), Ins(1,2,3,4,6)P_5_ (InsP_5_ [5-OH]), and D-and/or L-Ins(1,2,4,5,6)P_5_ (InsP_5_ [1/3-OH]) (Figure S2). Such a lack of specificity toward the site of initial attack on InsP_6_ is a defining characteristic of MINPPs of both plants (Dionisio et al., 2007) and animals (Figure S1A; Ali et al., 1995, Craxton et al., 1997). This catalytic flexibility contrasts with the more precise positional specificity of phytases. In fact, the distinct specificities of different types of phytases are sufficient to serve as a classification system: there are 3-phytases (E.C. 3.1.3.8) (see also Figure S1B), 6-phytases (E.C. 3.1.3.26), and 5-phytases (E.C. 3.1.3.72).

Although mammalian MINPPs exhibit positional promiscuity toward InsP_6_ (Craxton et al., 1997), these enzymes are highly specific toward certain other inositol phosphate substrates. For example, they primarily remove the 3-phosphate from Ins(1,3,4,5,6)P_5_ and Ins(1,3,4,5)P_4_ (Craxton et al., 1997) and display a considerably higher V_max_ toward Ins(1,3,4,5)P_4_ substrate than to InsP_6_ (Nogimori et al., 1991). The *Bacteroides* enzyme shares with mammalian MINPPs increased catalytic activity toward Ins(1,3,4,5)P_4_ substrate (Figure S2A), yielding a V_max_ value 20- to 36-fold higher than that obtained with InsP_6_ (Table S2). In contrast, incubation of BtMinpp with Ins(1,3,4,5)P_4_ (Figure S2A) yielded two InsP_3_ peaks that coeluted with Ins(1,4,5)P_3_ and Ins(1,3,4)P_3_ standards. The pH optima of 2.5, 4.0, and 7.5 (Figure S3) overlaps with the pH profile of the human GI tract (Fallingborg, 1999), consistent with the successful adaptation of BtMinpp to this environment.

### The Crystal Structure of BtMinpp in Complex with a Substrate Analog

**Figure 2:**
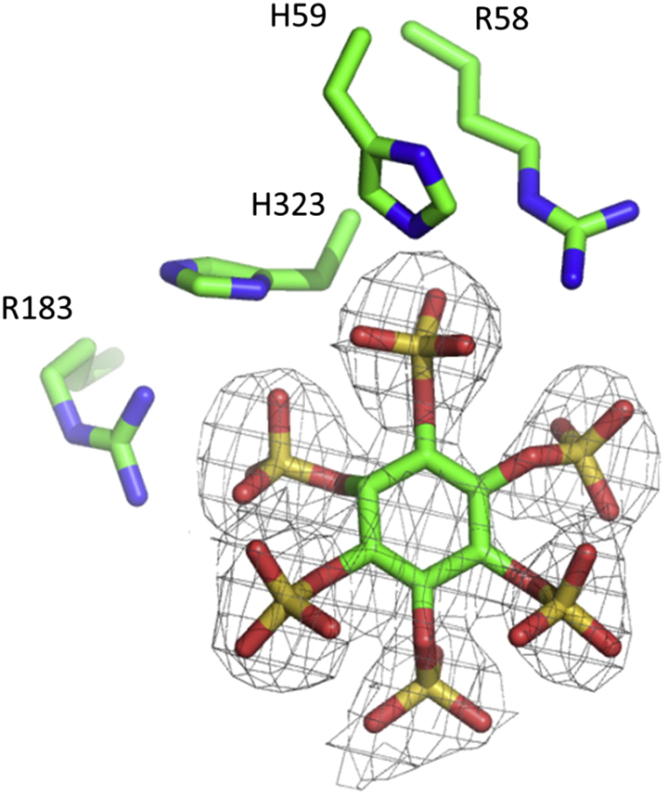
Simulated Annealing Omit Map Revealing the Location of InsS_6_ Bound to the Active Site of BtMinpp A region of the simulated annealing omit electron density map (gray lines) (Brünger and Rice, 1997) calculated with data to a resolution of 2.42 Å and contoured at 1.8 σ. Selected active site residues are shown in stick representation and labeled. The location of the inhibitor in the final refined structure of the complex is shown superimposed on the omit map electron density.

There is no published description of the structures of any members of the MINPP family. The crystal structure (diffraction data were collected on beamlines I02 and I24 at the Diamond Light Source, UK) of BtMinpp expressed in *Escherichia coli* as an N-terminal 6 × His fusion protein was solved at pH 5.0 by SAD using phases from a selenomethionyl derivative. Refinement against native data at 1.93 Å resolution gave a final structural model with an *R*_cryst_ of 16.6% (*R*_free_ 21.3%) and excellent geometry. A single inorganic phosphate group is bound at the active sites of both molecular copies of BtMinpp in the crystallographic asymmetric unit, presumably scavenged from the phosphate buffer used during purification of the enzyme (Table 1; Figure S4). A second structure was solved by difference Fourier analysis at 2.42 Å resolution in which the inorganic phosphate ions are displaced by the nonhydrolysable InsP_6_ analog, *myo*-inositol hexakissulfate (InsS_6_), again bound in the active sites of both molecules (*R*_cryst_ 15.6%; *R*_free_ 21.7%) (Figure 2; Table 1). Comparison of the structures of analog- and phosphate-bound forms revealed an rmsd of 0.20 Å for C_α_ atoms suggesting limited conformational changes occur between the two states.

**Figure 3:**
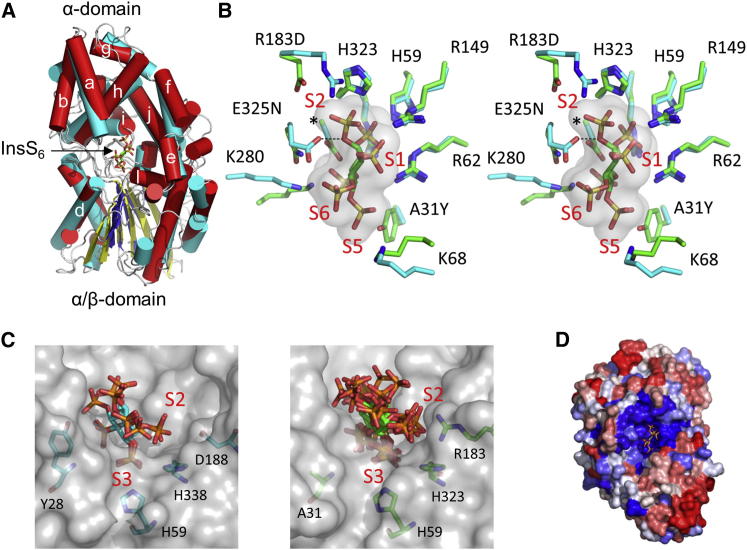
X-Ray Crystal Structure of the BtMinpp (A) Cylinder and ribbon diagram showing a superposition of the crystal structures of BtMinpp (α helices in red, β sheet in yellow) and the *Aspergillus niger* phytase PhyA (Oakley, 2010) (α helices in cyan, β sheet in blue) both taken from their complexes with the InsP_6_ analog, InsS_6_. The core α+β and capping α-domains are indicated. The naming of α helices in the capping domain of BtMinpp (indicated by a lowercase letter) follows that suggested for PhyA (Oakley, 2010). The atoms of the bound substrate analog are shown as sticks. (B) A stereoview of the superposition of the side chains of InsS_6_-binding residues (shown as sticks) of BtMinpp (carbon atoms colored cyan) and *Aspergillus niger* PhyA (carbon atoms colored green). The residues selected for display form the primary sites of interaction with InsS_6_ in both complexes. The van der Waals surface of InsS_6_ is shown in gray. Residue labels follow the numbering for the BtMinpp structure. The leading character identifies the residue in BtMinpp, whereas the trailing identifies the corresponding variant residue found in PhyA. An asterisk (^∗^) indicates the position of the A324D substitution. Selected binding pockets (S1, S2, S5, and S6) are indicated, numbered according to corresponding sulfate group on the bound ligand. Pocket S4 is obscured in this orientation by the 6-sulfate group of the ligand. The interaction distance between a carboxyl group oxygen of residue E325 and the bridging ether oxygen of the S3 sulfate group of InsS_6_ is 3.3 Å and is indicated by a dashed line. (C) Predicted binding modes within 1 kcal mol^-1^ of the minimum binding energy resulting from molecular docking experiments for InsP_6_ with PhyA (left, three modes) and BtMinpp (right, seven modes). Selected active site residues are shown as sticks and labeled as are binding pockets S2 and S3. The enzyme molecular surfaces are shown in gray. (D) View of a ConSurf (Landau et al., 2005) color-coded surface representation of BtMinpp. The normalized conservation scores calculated by ConSurf are a relative measure of evolutionary conservation at each residue position based on the alignment of 23 MINPP sequences. The highest scores (8 and 9 on the ConSurf scale) represent the most conserved residue positions and are shown colored blue. The residues with the lowest scores (i.e., the most variable) are colored red. The bound InsS_6_ ligand is shown in stick format.

BtMinpp folds into two domains, an α/β domain and an α domain. Despite their low amino acid sequence homology, and functional diversity, all available crystal structures of members of branch 2 of the histidine phosphatase superfamily possess this general domain arrangement with a structurally conserved α/β domain (the “core” domain) and a more variable α-domain (the “cap” domain) (Rigden, 2008). The cleft between the two domains always contains the active site, which is highly specialized in each of the distinct branch two subfamilies, allowing them to perform different functions. The active site in BtMinpp is lined with predominantly basic amino acids, thereby rationalizing how the enzyme can bind unusually highly phosphorylated substrates such as InsP_6_ (Figure 3A). InsS_6_ is bound in the active site with its 3-sulfate group occupying the position taken by inorganic phosphate in the enzyme-phosphate complex and presumably that of the hydrolyzed phosphate group in substrates. This conclusion is consistent with the sulfur atom of this group lying 3.1 Å from the imidazole Nε2 atom of the side chain of H59. By analogy with other members of the superfamily, we predict that H59 of BtMinpp acts as a nucleophile during catalysis to generate a phosphohistidine intermediate. In phytases, the aspartate residue from a proximal HD sequence motif takes the role of proton donor for the leaving group (Kostrewa et al., 1997). However, a distinguishing feature of mammalian and plant MINPPs is the substitution of this motif with an HAE tripeptide in which the glutamic acid residue (E325 in BtMinpp) is the candidate proton donor. Indeed, in our crystal complex with InsS_6_ one of the E325 side-chain carboxyl oxygen atoms is only 3.3 Å from the ether oxygen bridging the 3-sulfate to the inositol ring, the analog to the phosphate-leaving group in InsP_6_.

The structure of the InsS_6_-protein crystal complex rationalizes the ability of BtMinpp to remove the 3-phosphate from its natural substrate, InsP_6_. However, BtMinpp also removes alternative phosphates from InsP_6_ (Figure 1A), indicating that the enzyme’s active site can bind substrate in alternate orientations. To analyze which features of the catalytic core of BtMinpp permit this positional promiscuity, we compared its structure with that of *A. niger* PhyA phytase (Oakley, 2010), a member of a different subfamily of clade 2 histidine acid phosphatases, that hydrolyzes InsP_6_ by specific removal of its 3-phosphate (as does the closely related *A. ficuum* PhyA; Figure S2B). A comparison of this PhyA with BtMinpp yielded an rmsd of 2.5 Å, albeit with low sequence identity (16% over 360 structurally equivalent residues; Table S3). The overall topologies of these two enzymes are superficially similar, which is why we have labeled those secondary structural elements in BtMinpp that correspond to those found in PhyA (Figures 3A and S5).

Accordingly, we performed an overlay of the InsS_6_-bound structures of BtMinpp and *A. niger* phytase based on the coordinates of the backbone atoms of the active site fingerprint sequence (RHGXRXP; residues 58–64 in BtMinpp) found in the A-a loop and those of the short region containing the presumed proton donor, E325 (HAE; residues 323–325) from the N-terminal region of helix l. The rmsd was 0.30 Å for these 30 atoms. The conformations of InsS_6_ were very similar (rmsd 0.50 Å for the six inositol ring carbon atoms) in these overlayed structures. Nevertheless, this superposition (Figure 3B) revealed some significant differences in the nature of the ligand-binding residues in the two proteins. Binding pocket S2 shows the most extensive differences. Here, R183, conserved in MINPP enzymes and forming an ion pair with the 2-sulfate of the ligand, replaces the Asp in PhyA. Additionally, the HAE tripeptide of BtMinpp replaces the characteristic HD sequence motif in PhyA. Both substitutions substantially alter the volume and character of S2. The substitution of the phytase proton donor (Asp) with Ala324 in BtMinpp increases volume and decreases polarity of both S2 and the adjacent S4 pockets. Finally, a tyrosine residue that contributes to pockets S4 and S5 is replaced by Ala31in BtMinpp. These substitutions at positions 31 and 324 make the S4 and S5 pockets of BtMinpp significantly larger than those in PhyA. The significance of the larger BtMinpp ligand-binding site is that it may permit alternate substrate binding modes. Molecular docking experiments further support the concept of a reduced discrimination against alternative InsP_6_-binding modes in BtMinpp compared to PhyA (Figure 3C; Table S4): PhyA showed an exclusive preference for a limited set of highly similar InsP_6_ binding modes all presenting the D-3 phosphate in binding pocket S3. Termed the obverse binding mode, this places the D-2 axial phosphate in the S2 binding pocket. BtMinpp was more permissive, allowing binding of not only the D-3 phosphate in pocket S3 but also the D-5 and D-6 phosphates. These additional binding modes present equatorial phosphates of InsP_6_ in the S2 pocket and are distributed between obverse and reverse presentations (the latter having the inositol ring flipped over to present its opposite face to the enzyme). Given that the A31Y and R183D substitutions in phyA relative to BtMinpp were identified by our structural studies as making particularly significant contributions to the volume and polarity of the active site, we tested that hypothesis with a mutagenic approach.

### Impact of Mutating the Active Site of BtMinpp on the Specificity of Initial Attack on InsP_6_ Substrate

When incubated with InsP_6_, the A31Y mutant of BtMinpp yielded an HPLC profile of InsP_5_ products (Figure 1B) very similar to that observed for wild-type enzyme (Figure 1A). Thus, the positional specificity toward InsP_6_ was not affected by this mutation. Nevertheless, it should be noted that the HPLC profile of the wild-type enzyme was obtained with one-tenth the protein concentration of that of the A31Y mutant (we estimated that the A31Y mutant was approximately 2.5-fold less active). Elevating the wild-type enzyme concentration 10-fold resulted in complete dephosphorylation of InsP_5_ and InsP_6_ (Figure S2B). Remarkably, the R183D substitution abolished the production of InsP_5_ [4/6-OH], but retained a peak with the retention time of InsP_5_ [5-OH]/ InsP_5_ [1/3-OH] (Figure 1C). Using an Adsorbosphere SAX column to resolve InsP_5_ [5-OH] from InsP_5_ [1/3-OH] and InsP_5_ [2-OH], we confirmed that the R183D mutant had lost the ability to attack the 4/6-position (compare Figures 1A and 1C), but retained the ability to attack the 5- and 1/3-positions (Figure 1C, inset). Thus, our mutagenic data confirm that the positional flexibility of BtMinpp toward InsP_6_ reflects a larger and less discriminating active site compared to phyA. The R183D mutant was also about 10-fold less active than wild-type enzyme (compare Figures 1A and 1C), suggesting that increased catalytic activity may be an evolutionary advantage of BtMinpp’s more flexible active site.

Given that human MINPP1 and BtMinpp share a common ability to attack InsP_6_ substrate at different positions and remove a 3-phosphate from Ins(1,3,4,5)P_4_ (see above), we considered whether our crystallographic analysis of BtMinpp offers an explanation of the substrate specificity of mammalian MINPPs. Analysis of the conservation of residues that are predicted to contribute atoms to the molecular surface of 23 representative MINPP enzymes from different kingdoms of life reveals highest sequence identity in a region of the active site centered on H59 (Figure 3D). There is 52% sequence identity between human MINPP1 and BtMinpp for those active site residues that have an atom within 8 Å of the InsS_6_ bound to the bacterial enzyme. Interestingly, residue A31, forming part of binding pocket S5 in BtMinpp, is commonly replaced with a lysine in other MINPP enzymes (Figure S5). Introduction of a lysine residue may allow additional polar contacts with highly negatively charged substrates. Its size may also serve to make binding pocket S5 smaller. This substitution may therefore be of relevance in explaining the differential activity of *Bacteroides* and eukaryotic enzymes toward lower inositol phosphate substrates by enforcing more positional specificity on the mammalian enzyme.

### The MINPP Protein Family Includes Microbial Members

Having defined Minpp as a bacterial product, we next performed a phylogenetic analysis to determine how widespread is the distribution of *minpp* homologs in microbial organisms. A BLASTP search of BtMinpp encoded protein sequence against the nonredundant protein database identified 326 sequences with significant alignments (e value ≤ 1 × 10^−4^ and a minimal number of 60 identical amino acids over the entire sequence length). These included proteins of bacterial, animal, and plant origin. Whereas most bacterial phyla were represented, no representative of the Firmicutes, a major phylum of the human GI tract microbiota, was included. Nevertheless, a significant number of the other dominant GI-resident organisms (*Bacteroides*, *Bifidobacterium*, *Prevotella*, and *Alistipes)* encode proteins belonging to the MINPP family. In contrast, a search for microbial genes predicted to encode microbial phytase enzymes among 341 genomes of the human GI tract (Human Microbiome Project at http://commonfund.nih.gov/hmp/index) revealed that microbial phytases are scarce in this environment and are only present in the genome of subdominant bacterial species (Table S5). This analysis leads us to conclude that bacterial Minpps are the major representative of InsP_6_-degrading enzymes within the GI tract of humans and are encoded by dominant bacterial species that are niche-adapted human symbionts.

**Figure 4:**
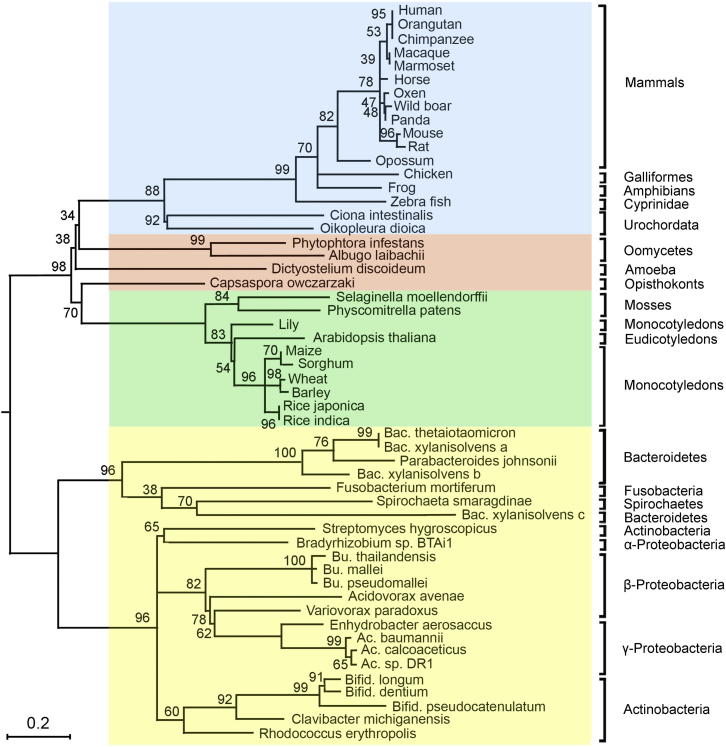
The MINPP Family Phylogenetic Tree A phylogenetic tree derived from the alignment of 54 MINPP protein representatives from different kingdoms of life was constructed using the Maximum Likelihood method. The different kingdoms have been assigned the following background colors: animals, blue; plants, green; protists, salmon-pink; bacteria, yellow. Ac, *Acinetobacter*; Bac, *Bacteroides*; Bu, *Burkholderia*; Bifid, *Bifidobacterium*. The tree is drawn to scale, with branch lengths measured in the number of substitutions per site.

A phylogenetic tree (Figure 4) was constructed from the alignment of 54 representatives (Table S6) of different kingdoms of life. In general, MINPP-related sequences are found within the bacteria, plant, and animal kingdoms with the sequences in plants and animals being evolutionarily conserved within their respective clade. However, in the bacterial group, the Bacteroidetes phylum does not group with the Actinobacteria phylum as previously reported (Ciccarelli et al., 2006). The different Minpps from the bacterial species displayed in the tree all appear equally related to their eukaryotic homologs (Figure 4) with the eukaryotic and bacterial proteins each forming monophyletic groups, suggesting an ancient origin for the MINPP family of proteins.

Among the 2,536 completed bacterial genomes listed in EMBL-EBI (http://www.ebi.ac.uk/genomes/bacteria.html), only 55 contained one or more copies of a gene homologous to BtMinpp, accounting for 2.2% of the completed genomes. In contrast, greater than 50% of animal and plant completed genomes contained at least one copy of a gene predicted to encode a MINPP representative. Our phylogenetic analyses strengthen the conclusion from our biochemical and structural studies (see above) that the bacterial Minpp-related protein sequences constitute a different family, distinct from phytases, but nevertheless a subset of clade 2 of the histidine phosphatase superfamily (HAP; IPR000560) (Rigden, 2008).

### InsP_6_ Metabolism by BtMinpp Secreted from *B. thetaiotaomicron* in Outer Membrane Vesicles

**Figure 5:**
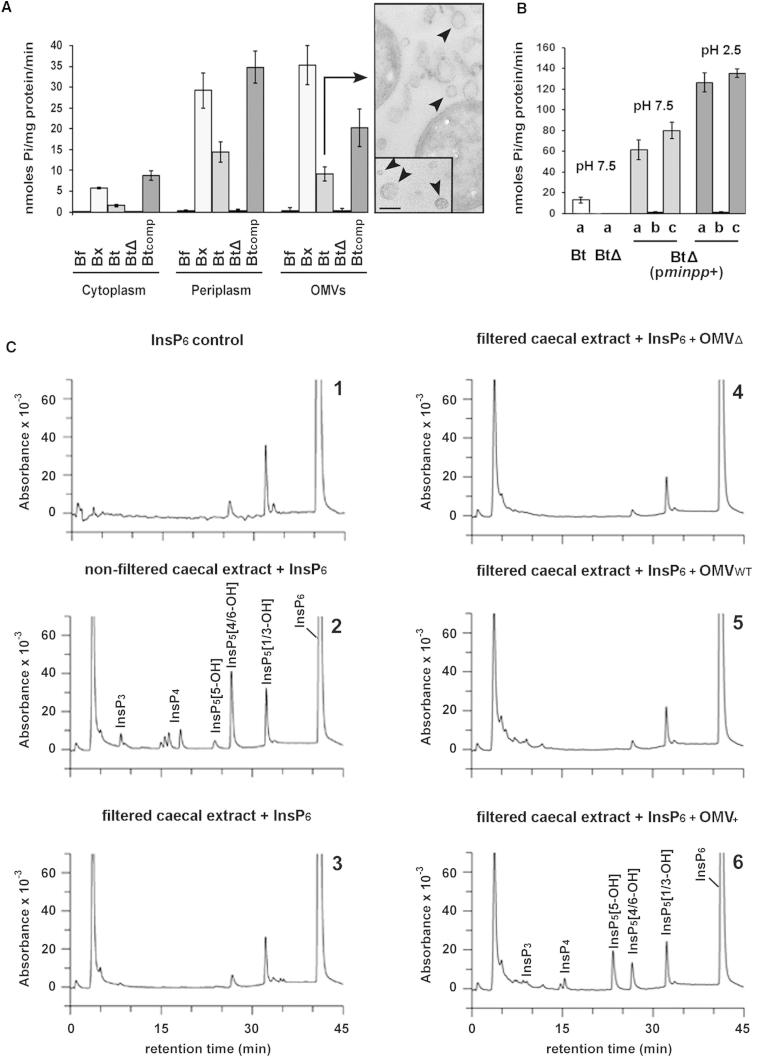
Intact OMVs Produced by *B. thetaiotaomicron* Degrade Exogenous InsP_6_ (A) Minpp activity was assessed in different fractions of *B. thetaiotaomicron* and other *Bacteroides* spp. using InsP_6_ as a substrate and a molybdate/malachite green-based enzyme assay to measure released phosphate. The different fractions were extracted from *B. fragilis* (Bf), *B. xylanisolvens* (Bx), *B. thetaiotaomicron* (Bt), Bt *minpp*-deleted mutant strain GH59 (Bt_Δ_), and the same strain containing the pGH38 plasmid (GH120) expressing BtMinpp (Bt_comp_). The values shown represent the mean ± SEM values obtained from five to six independent experiments. The TEM images show Bt cells and associated OMVs (arrowed) with the inset showing images of isolated OMVs. Scale bar, 100 nm. (B) (a) Degradation of InsP_6_ by intact OMVs produced by *B. thetaiotaomicron* wild-type strain (Bt), the *minpp*-deleted mutant (Bt_Δ_), and the *minpp*-deleted mutant containing the pGH037 plasmid overexpressing BtMinpp (Bt_Δ_ (p*minpp*+). OMVs were isolated, concentrated, and resuspended in a Tris buffer (pH 7.5) and for Bt_Δ_ (p*minpp*+) in a glycine-HCl buffer (pH 2.5) to which InsP_6_ substrate was added and the suspension was incubated for 1 hr at 37°C. (b) BtMinpp activity was measured in the buffer fraction of Bt_Δ_ (p*minpp*+) OMV suspensions after incubation for 1 hr at 37°C and removal of OMVs. (c) Enzyme activity was measured in sonicated extracts of OMVs recovered from the mixtures described in (b). The values shown represent the mean ± SEM values obtained from at least five independent experiments. (C) Cecal contents were obtained from C57BL/6 mice as described in the Experimental Procedures. Each HPLC chromatogram panel shown is representative of samples from four different mice. InsP_6_ was added to all samples. (C1) InsP_6_ control, showing contaminating InsP_5_s of this commercial InsP_6_ sample; (C2) nonfiltered cecal extract; (C3) cecal extracts filtered (100 kDa molecular weight cutoff membrane); (C4–6) cecal extracts filtered (100 kDa molecular weight cutoff membrane) and supplemented with OMVs from either a BtMinpp-deleted mutant (C4), the WT strain (C5), or a strain overexpressing Minpp (C6).

The key question of how BtMinpp in the gut survives a hostile protease-containing environment yet accesses extracellular InsP_6_ was addressed. BtMinpp is predicted to contain an N-terminal signal peptide (http://www.cbs.dtu.dk/services/SignalP/), so we hypothesized that the protein would be secreted into the periplasmic space. Indeed, the periplasmic fraction of *B. thetaiotaomicron* showed substantial Minpp activity (Figure 5A). This observation led us to investigate if BtMinpp might also be packaged into outer membrane vesicles (OMVs). Although OMV blebbing from the cell surface has been observed in different *Bacteroides* species, *B. fragilis* is the only example for which various enzymatic activities in OMVs have previously been assayed (Patrick et al., 1996). Transmission electron microscopy confirmed the presence of membrane blebs and the release of intact OMVs by *B. thetaiotaomicron* (Figure 5A). Substantial InsP_6_ phosphatase activity was detected in OMV protein extracts (Figure 5A).

We prepared the protein fraction from the cytoplasm, periplasm and OMVs of a strain of *B. thetaiotaomicron* from which we had deleted the *minpp* gene. All of these fractions had negligible InsP_6_ phosphatase activity (Figure 5A), indicating that BtMinpp accounts for the InsP_6_ phosphatase activity observed in the corresponding fractions prepared from the wild-type strain (Figure 5A). Expression of *minpp* in *trans* in the mutant increased the activity to levels higher than in the wild-type parental strain in each of the subcellular fractions (Figure 5A). It is noteworthy that no InsP_6_ hydrolysis activity was detected in another human gut *Bacteroides* species, *B. fragilis*, that does not contain a gene related to *minpp.* In contrast, high levels of InsP_6_ phosphatase activity were detected in the periplasm and OMVs of *B. xylanisolvens* (Figure 5A), which contains three copies of the *minpp* gene.

We next investigated the capacity of intact vesicles containing BtMinpp to degrade InsP_6_ in the external milieu. InsP_6_ was hydrolyzed by OMVs produced and isolated from the wild-type strain, whereas no phosphate release from InsP_6_ was detected by OMVs produced by the Minpp1-deficient strain (Figure 5B). We also engineered a *B. thetaiotaomicron* strain that overexpresses BtMinpp and recorded between 6 and 12 times more InsP_6_ degradation for its isolated OMVs compared to those from wild-type *B. thetaiotaomicron* (Figure 5B). The conditioned media from which OMVs had been removed showed no enzyme activity; sonication of the OMVs was required for enzyme activity to be released (Figure 5B). These findings are consistent with OMVs being intact and that the enzyme is retained in the vesicles and is not released due to leakage.

The functional significance of this packaging of BtMinpp in OMVs was investigated in physiological experiments using the cecal contents of mice to which InsP_6_ substrate was added and using HPLC to detect InsP_6_ metabolites. Note that in these experiments the commercial InsP_6_ sample was contaminated with small peaks of InsP_5_ (Figure 5C1). Control cecal extracts contained InsP_6_-phosphatase activity (Figure 5C2) that was no longer detected after filtration using a 100 kDa cutoff membrane (Figure 5C3), capable of removing large protein complexes or OMVs produced by resident bacteria. In further experiments, no significant InsP_6_-phosphatase activity was observed in filtered cecal extracts supplemented with OMVs prepared from Minpp-deficient bacteria (Figure 5C4). The assay was also unable to detect InsP_6_ phosphatase activity in OMVs produced by wild-type *B. thetaiotaomicron* (Figure 5C5), probably due to limitations in its sensitivity (see Figure 5B). However, when filtered cecal contents were supplemented with OMVs produced by *B. thetaiotaomicron* that overexpress Minpp, considerable InsP_6_-phosphatase activity was detected (Figure 5C6). Collectively, these findings demonstrate that BtMinpp is retained inside the OMVs, which InsP_6_ must enter in order to access the BtMinpp.

### Minpp1-Loaded Vesicles Induce the Release of Intracellular Calcium in Colonic Epithelial Cells

**Figure 6:**
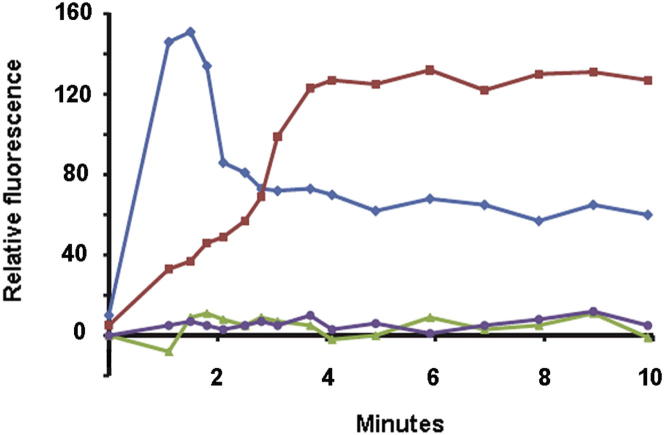
Minpp1-Loaded Vesicles Modify Colonic Epithelial Cell Signaling Fluorescence intensity of Fluo-8 AM-loaded HT29 cells in response to treatment with ionomycin (blue), OMVs from a BtMinpp-deleted mutant (purple), OMVs containing the overexpressed BtMinpp (red), or PBS alone (green). The data shown are representative of three experiments, each with three replicates.

It is well established that OMVs produced by bacterial pathogens such as *Helicobacter pylori*, *Legionella pneumophila*, or *E. coli* are capable of interacting with host cells via a membrane fusion event or via adhesin-receptor-mediated attachment to deliver virulence factors such as proteases and toxins (Ellis and Kuehn, 2010). We hypothesized therefore that either mechanism might be used by *B. thetaiotaomicron* OMVs to deliver BtMinpp into host intestinal epithelial cells, with the potential consequence that BtMinpp could interact with the inositol polyphosphate signaling pathways of host cells. Indeed, Yu et al. (2003) have shown that in mammalian cells expression of an engineered truncated cytosolic form of the endoplasmic reticulum-confined MINPP1 leads to a significant enhancement of Ins(1,4,5)P_3_ concentration, triggering the release of calcium from intracellular stores via the InsP_3_ receptor/Ca^2+^ channel. To test these ideas, BtMinpp-containing OMVs were added to HT29 human colonic epithelial cells in the absence of extracellular calcium. Immediately after addition of the OMVs, a gradual increase in intracellular calcium concentration was observed that plateaued within approximately 4 min (Figure 6). By contrast, no calcium response was observed when HT29 cells were incubated with OMVs that did not contain any BtMinpp. This result suggests that OMVs interact with epithelial cells leading to the release of BtMinpp and generation of InsP_3_ products. Additionally, it is possible that attachment of OMVs to target cells may locally increase the concentration of an InsP_6_ metabolite that stimulates Ca^2+^ mobilization. Nevertheless, irrespective of their mechanism of action, OMVs containing BtMinpp are able, in vitro, to trigger the release of calcium from intracellular stores to the cytosol of colonic epithelial cells.

## Discussion

The main impact of our study centers on the identification of a homolog of a eukaryotic inositolphosphate phosphatase, MINPP, in major species of human gut bacterial genomes. Detailed biomolecular and phylogenetic analyses of Minpp from *B. thetaiotaomicron* (BtMinpp) validated it as a member of the MINPP family with an exceptionally high catalytic activity that makes it exquisitely suited for facilitating InsP_6_ homeostasis in the mammalian GI tract. Moreover, we rationalize the catalytic activity and evolutionary conservation of BtMinpp by mutagenic studies and by an atomic-level description of the structure of this enzyme at 1.9 Å resolution. We further demonstrate that BtMinpp is packaged inside OMVs, thereby protecting the phosphatase activity from degradation by gastrointestinal proteases, and also facilitating an example of cross-kingdom, long-range, cell-to-cell signaling; we show that the OMVs that are released by *B. thetaiotaomicron* are able to deliver BtMinpp to intestinal epithelial cells, promoting intracellular Ca^2+^ signaling.

The physiological significance of InsP_6_ phosphatase activity in the human gut is likely complex and multifactorial. First, there is nutritional benefit to both the host and the bacterial community from the inorganic phosphate and the inositol moiety that are both released. Additionally, the hydrolysis of InsP_6_ eliminates its antinutritive properties, such as divalent ion chelation and inhibition of polysaccharide digestibility. High concentrations of InsP_6_ have been considered to have anticarcinogenic properties in the human colon (Fox and Eberl, 2002, Vucenik and Shamsuddin, 2003). However, a recent paper indicates that lower and more physiologically relevant levels of InsP_6_ in the diet might promote tumor development (Windhorst et al., 2013). This last finding raises the possibility of further human health impacts of bacterial Minpp in the GI tract.

*B. thetaiotaomicron* is a dominant human GI symbiont. Moreover, our phylogenetic studies indicate that the majority of other bacteria known to harbor a *minpp* gene are also members of the human intestinal microbiota. These 149 representatives are from the *Bacteroides*, *Bifidobacterium*, *Prevotella*, and *Alistipes* communities. Indeed, our analysis of InsP_6_ hydrolysis ex vivo from endogenous cecal contents indicates that the endogenous enzymatic activity is positionally promiscuous toward its substrates, and contained in OMVs. These are both properties of BtMinpp making it likely that bacterial Minpps are a widespread yet hitherto unappreciated source of highly active InsP_6_ phosphatase activity in the GI tract.

Our characterization of the crystal structure of BtMinpp provides a description of members of the MINPP family at the atomic level. In particular, the structure of the protein in complex with InsS_6_, a substrate analog, gave insight into the reaction mechanism. We propose that H59 acts as a nucleophile for cleavage of the 3-phosphate from InsP_6_, with E325 in an HAE tripeptide being the proton donor for the leaving group. Our structural work also illuminates the positional promiscuity of the MINPP superfamily, which distinguishes it from the various classes of phytases in the HAP family that each exhibits a distinct preference for a particular phosphate group (e.g., 3-phytases, 4/6-phytases, and 5-phytases; Konietzny and Greiner, 2002, Kumar et al., 2010). Our overlay of the structure of the active sites of *A. niger* 3-phytase and BtMinpp revealed that the latter has a larger and less polar ligand-binding pocket that, according to our molecular models, accepts InsP_6_ in several different orientations (Figure 3C). Furthermore, in a synthetic biology approach, we mutated R183 in BtMinpp to the corresponding Arg in the 3-phytase in *A. niger*. This resulted in BtMinpp losing the ability to remove the 4/6-phosphate from InsP_6_ (Figure 1). That is, the R183D mutation caused the positional preference of the enzyme to become more phytase-like in nature.

Interestingly, A31 in BtMinpp is commonly replaced with Lys in other MINPPs (Figure S5), and so this residue may be of relevance in explaining the differential positional specificities of *Bacteroides* and eukaryotic enzymes toward lower inositol phosphate substrates. For example, the mammalian MINPPs only removes the 3-phosphate from Ins(1,3,4,5)P_4_ (Caffrey et al., 1999), whereas the bacterial enzyme is less selective. It is an intriguing possibility that a lack of positional selectivity may be associated with elevated catalytic activity of BtMinpp against InsP_6_. Nevertheless, we found a 52% sequence identity between human MINPP1 and BtMinpp for those active site residues that have an atom within 8 Å of the InsS_6_ that cocrystallized in the active site. Such a high degree of evolutionary conservation would suggest that bacterial and mammalian Minpps have originated from a common primordial ancestor. It is alternately possible that bacteria acquired Minpps from eukaryotes by horizontal gene transfer (HGT), although known cases of this phenomenon are rare (Keeling and Palmer, 2008). Our phylogenetic analyses were unable to distinguish between these two alternatives.

Our characterization of the properties of a vesicularized bacterial homolog of a mammalian signaling enzyme challenges established orthodoxies concerning our understanding of the mechanisms by which symbiotic bacteria in the mammalian GI tract interact with their host. First, it has been thought that OMVs primarily mediate pathogenic processes (Ellis and Kuehn, 2010). Our data reveal that commensal gut bacteria also utilize OMVs in a manner that is beneficial to the host, by contributing to InsP_6_ homeostasis. The ability of BtMinpp-containing OMVs to stimulate intracellular Ca^2+^ release in human colonic epithelial cells suggests a further biological significance to bacterial Minpps, namely, a role in interkingdom communication pathways (Figure 6). Another example of this phenomenon is the OMV-mediated delivery of immunoregulatory capsular polysaccharide A from *B. fragilis* to host dendritic cells to effect disease protection (Shen et al., 2012). Nevertheless, the application of an enzyme to mediate dialog between gut bacteria and the human host is an addition to a field of research that has previously focused on the roles of diffusible, small-molecule hormones and nutrients. Further research into variations in the expression and secretion of Minpps by the different species of GI bacteria could increase our insight into the complex interrelationships between the microbiome and the human host.

## Experimental Procedures

### Bacterial Strains and Growth Conditions

All *E. coli* and *Bacteroides* strains used in this study are listed in Table S7. The bacterial growth conditions are described in the Supplemental Information.

### HPLC

Inositol phosphate products of assays using ^32^P-labeled substrate were resolved by HPLC, details of which are provided in the Supplemental Information.

### Crystal Structure Determination

A single crystal of selenomethionyl-derivitized BtMinpp was used to collect a SAD data set, which was processed using the CCP4 package to obtain a crystal structure as described in the Supplemental Information.

### Phylogenetic Analysis

The evolutionary history of the MINPP protein was inferred using the Maximum Likelihood method in the MEGA5 software tool (Tamura et al., 2011). Amino acid sequences were aligned with PRANK and highly variable regions removed from the data set. The Whelan and Goldman (2001) model of evolution was used, and initial tree(s) for the heuristic search was obtained automatically as follows. When the number of common sites was <100 or less than one-fourth of the total number of sites, the maximum parsimony method was used; otherwise, BIONJ method with MCL distance matrix was used. To provide statistical support for each node on the tree, a consensus tree was generated from 1,000 bootstrap data sets.

### Periplasmic and OMV Protein Extraction and BtMinpp Phosphatase Activity Measurement

The method of Osborn et al. (1972) was used to obtain periplasmic fractions of *B. thetaiotaomicron* and OMVs were harvested from bacterial cultures by ultracentrifugation and assayed for phosphatase activity using the PiColorLock Gold Phosphate Detection System as described in the Supplemental Information.

### Intracellular Calcium Measurement

HT-29 cells were propagated in Sarstedt culture flasks (25 cm^2^) in a 5% CO_2_ humidified atmosphere at 37°C. Cells were fed with DMEM (Lonza) supplemented with 10% heat-inactivated FBS (Biosera) and 2 mM L-glutamine (Lonza). For the intracellular calcium measurement assay, cells were seeded in 24-well microplates (Corning CellBind Surface) at a density of 10^5^ cells/well. After 16 hr, the culture medium was replaced with fresh medium containing 5 μM of the calcium indicator Fluo-8 AM (AAT Bioquest), 4 mM of Probenecid (Sigma-Aldrich), and 0.025% (w/v) Pluronic acid F-127 (Invitrogen) according to a method adapted from Abrahamse and Rechkemmer, 2001, and cells were incubated for 30 min at 37°C, 5% CO_2_. The medium was removed, and calcium-free PBS was added before the cells were incubated at 37°C for another 30 min. The cell monolayer was washed twice with calcium-free PBS and PBS was added. Fluo-8 fluorescence was measured immediately after addition of Ionomycin (Sigma-Aldrich) 1 μg/ml, PBS, or purified OMVs (corresponding to 10 μg of soluble protein) with a FLUOstar Optima fluorescence plate reader (BMG Labtechnologies) fitted with custom excitation/emission filters (485/538 nm). OMVs were purified from 500 ml culture supernatants, washed with calcium-free PBS, and concentrated to a 2 ml suspension.

## Author Contributions

R.S. and S.R.C. conceived the study and R.S., S.R.C., C.A.B., S.B.S., and A.M.H. wrote the paper. R.S., S.R.C., C.A.B., A.M.H., P.B., and S.B.S. designed experiments and analyzed data. R.S., S.O., N.H., A.W.H.L., C.A.B., I.H., R.B., M.R., and S.B.S. performed the experiments. S.R.C. and S.B.S. obtained funding to support the study.

## Figures and Tables

**Table 1:** X-Ray Data Collection and Structure Refinement Statistics

	SAD Data	Phosphate^a^	InsS_6_^a^
**Data Collection**			

Wavelength (Å)	0.9799	0.9778	0.9778
Space group	P 2_1_	P 2_1_	P 2_1_
Cell parameters			
a, b, c (Å)	53.1, 121.0, 76.0	52.7, 120.6, 76.1	52.2, 117.3, 75.5
β (°)	107.8	107.9	107.46
Resolution limits (Å) (high-resolution bin)^b^	50.00–2.50 (2.64–2.50)	62.07–1.93 (1.98–1.93)	61.44–2.42 (2.48–2.42)
R_sym_^c^	0.098 (0.265)	0.048 (0.473)	0.069 (0.529)
<(I)/sd(I) >	16.4 (7.3)	12.6 (2.1)	10.5 (2.9)
Completeness (%)	100.0 (100.0)	97.1 (97.0)	99.2 (99.2)
Multiplicity	7.4 (7.5)	2.3 (2.4)	3.3 (3.2)
Anomalous completeness (%)	99.9 (100.0)	—	—
Anomalous multiplicity	3.8 (3.8)	—	—
Overall temperature factor (Å^2^)		26.0	48.7

**Refinement**			

Protein monomers per asymmetric unit		2	2
Total nonhydrogen atoms		7194	6747
Water molecules		451	127
R_cryst_^d^ (%)		16.6 (23.5)	15.6 (19.7)
R_free_^e^ (%)		21.3 (28.2)	21.7 (27.0)
Ramachandran analysis (%)			
Most favored		97.58	97.37
Outliers		0.25	0.25
Rmsds			
Bonds, Å		0.007	0.008
Angles, °		0.976	1.17
Planes, Å		0.005	0.006
Mean atomic b value (Å^2^)		24.2	39.8

a Phosphate and IHS refer to the complexes of BtMinpp with inorganic phosphate and *myo*-inositol hexakis sulfate, respectively.

b Figures in brackets refer to the high resolution data bin as indicated.

c R_sym_ = Σ∣I_i_ - 〈I〉∣/ Σ I_i_ where 〈I〉 is the average of symmetry equivalent reflections and the summation extends over all observations for all unique reflections.

d R_cryst_ = Σ∣∣F_o_∣-∣F_c_∣∣/ Σ∣F_o_∣ where F_o_ and F_c_ are the measured and calculated structure factors, respectively

e For R_free_ the summations extends over a randomly selected subset (5%) of reflections excluded from all stages of refinement

## Accession Numbers

The Protein Data Bank ID codes for the atomic coordinates and structure factors for the phosphate- and InsS_6_-bound complexes of BtMinpp reported in this paper are 4FDT and 4FDU, respectively.

## Supplemental Information

Document S1. Supplemental Experimental Procedures, Figures S1–S5, Tables S1–S7

Document S2. Article plus Supplemental Information
