# Supplementary material for: A Bacterial Homolog of a Eukaryotic Inositol Phosphate Signaling Enzyme Mediates Cross-kingdom Dialog in the Mammalian Gut
Source: Cell Rep. 2014 Feb 13;6(4):646–56. doi: 10.1016/j.celrep.2014.01.021 (PMC3969271; doi:10.1016/j.celrep.2014.01.021)
Supplement: Document S1. Supplemental Experimental Procedures, Figures S1–S5, Tables S1–S7 [file mmc1.pdf]

Supplemental Information

Supplemental Experimental Procedures

Bacterial growth conditions

Purification of recombinant BtMinpp

BtMinpp enzymatic properties

HPLC

Inositol phosphates

$InsP_6$ assays

$InsP_4$ assay

Crystal structure determination

Molecular docking calculations

Site directed mutagenesis

Comparison of the structure of BtMinpp with other Branch 2 Histidine Phosphatases

Construction of *minpp* deletion mutant

Complementation of the *minpp* deletion mutant

Periplasmic protein extraction

Glucose-6-phosphate dehydrogenase assay

Outer membrane vesicles protein extraction

$InsP_6$ phosphatase activity measurement

Measurement of Minpp activity in the extracellular medium

Measurement of the catalytic activity of BtMinpp in resuspended OMVs

References

Supplemental Figure Legends

Figure S1, Related to Figure 1. The products of BtMinpp activity against $InsP_6$ are distinct from the products of attack of fungal phytase.

Figure S2, Related to Figure 1. The products of hydrolysis of Ins(1,3,4,5)P_4 and $InsP_6$

Figure S3, Related to Figure 1. Relative $InsP_6$ phosphatase activity (%) of recombinant BtMinpp at different pH values.

Figure S4, Related to Figure 2 Active site electron density for the complex of BtMinpp with inorganic phosphate.

Figure S5, Related to Figure 2. An alignment of the sequences of BtMinpp and selected MINPPs.

Supplemental Tables

Table S1, Related to Figure 1. Alignment of BT_4744 protein sequence with known MINPPs and phytases

Table S2, Related to Figure 1. Molecular and enzymatic properties of BtMinpp

Table S3, Related to Figure 2. Statistics from the comparison of BtMinpp with the structures of selected branch 2 histidine phosphatases

Table S4, Related to Figure 2.. Predicted low energy binding modes resulting from *in silico* docking of $InsP_6$ to enzyme structures

Table S5, Related to Figure 4. Genes annotated as phytases in the microbiome of the human gastrointestinal tract

Table S6, Related to Figure 4. Source organisms of MINPP proteins

Table S7, Related to Experimental Procedures. *E. coli* and *Bacteroides* strains used in the study

Materials and methods

Bacterial growth conditions

E. coli was grown at 37°C in Luria-Bertani medium (Sambrook & Russel, 2001) supplemented with 100 μg/mL ampicillin and/or 34 μg/mL chloramphenicol, 50 μg/mL kanamycin or 100 μg/mL spectinomycin. *Bacteroides* species were grown under anaerobic conditions at 37°C in BHI medium (Oxoid) supplemented with 0.001% haemin (BHIH). Antibiotic-resistance markers in *Bacteroides thetaiotaomicron* were selected using tetracycline 1 μg/mL, erythromycin 5 μg/mL or gentamicin 200 μg/mL. *E. coli* electrocompetent cells were prepared and transformed by the method of Sambrook and Russel (Sambrook & Russel, 2001).

Purification of recombinant and selenomethionine-labeled BtMinpp

The 405 amino acids product of the BtMinpp C-terminal region excluding the 20 amino acid N-terminal predicted signal peptide was purified using the His-Tag technique. A PCR fragment was generated using the primer pair MINPPam (CATGCATATGCAAACTAAGATACAGAAGTA) and MINPPav (CATGGGATCCCTATTCATTGAAAACTGA) and the resulting fragment was cloned into the *Nde*I/*Bam*HI restriction sites of the pET-15b expression vector (Novagen), which carries an N-terminal His-Tag sequence. The resulting plasmid pGH07 was used to transform BL21-CodonPlus(DE3)-RIL *E. coli* cells (Stratagene) and B834 (DE3) (Novagen) transformed with the pACYC-based tRNA-encoding plasmid RIL (Stratagene). The Ni-NTA Fast Start kit (Qiagen) was used to purify the protein according to the instruction of the manufacturer. Cells were grown at 30°C during 16 hours with a concentration of 0.5 mM IPTG. The imidazole used to elute the protein was removed using a PD-10 desalting column (Amersham Biosciences) and the buffer exchanged with 50 mM Tris-HCl, 300 mM NaCl pH7.2. The protein concentration was determined by direct UV measurement at 280 nm and by using the Bradford method. For the production of selenomethionine-labelled His-tagged BtMinpp, the plasmid pGH07 was transformed into the *E. coli* protease-deficient B834(DE3) strain (Novagen) containing the pACYC-based tRNA-encoding plasmid RIL (Stratagene)

using double selection with ampicillin (100 µg/mL) and chloramphenicol (34 µg/mL). The resulting strain was grown overnight in LB medium and 20mL of culture was used to inoculate 1 liter of Medium Base and Nutrient Mix (Athena Enzyme Systems) supplemented with 40 µg/mL L-selenomethionine solution (Athena Enzyme Systems). The cells were grown to an OD of 0.5 before induction with 0.5 mM IPTG. Cells were harvested after 16 h induction and recombinant selenomethionine-labelled proteins were extracted and purified using the Ni-NTA Fast Start kit (Qiagen) as described above.

BtMinpp enzymatic properties

Standard assay. $InsP_6$ phosphatase activity was measured using the PiColorLock Gold Phosphate Detection System (Innova Biosciences) based on the change in absorbance of the dye malachite green in the presence of phosphomolybdate complexes. 10 µM $InsP_6$ (Merck) was mixed with purified BtMinpp or protein extracts pre-treated with PiBind™ resin (Innova Biosciences) that removes contaminating Pi from buffers.

pH optimum and pH stability. The assay was performed using a variety of buffers: pH 1.0–3.5, 0.2 M glycine–HCl; pH 4.0–6.0, 0.05 M sodium acetate; pH 6.5–9.5, 0.2 M Tris–HCl. To determine the optimal pH, 2 ng of BtMinpp was incubated for 5 minutes at 37°C at different pH in the presence of 100µM $InsP_6$ and Pi release was measured under the standard $InsP_6$ dephosphorylation assay conditions. pH stability was determined after dilution and incubation of 10 ng of enzyme in different buffers and storage at 4°C for 24 hours.

Effect of temperature on enzyme activity. The temperature profile of the purified enzyme was determined in the temperature range from 10 C° to 80 C° using the standard assay. To check thermal stability, the purified enzyme was incubated at different temperatures for 10 minutes, cooled to 4 C°, and assayed using the standard $InsP_6$ dephosphorylation assay.

Effect of pepsin on enzyme activity. Enzyme inactivation by pepsin was determined by incubating 20 mU of BtMinpp in 0.2 M glycine–HCl pH 2.5, containing 3000 U pepsin for 30 min at 37 C°. After incubation, $InsP_6$ phosphatase activity was determined using the standard $InsP_6$ dephosphorylation assay.

HPLC

Inositol phosphate products of assays using ^{32}P-labelled substrate were resolved by HPLC on a 23.5 cm x 4.6 mm i.d.WVS Partisphere SAX column (Whatman, UK, Ltd) fitted with a SAX guard cartridge (Whatman). The column was eluted at 1 mL/min with a gradient formed by mixing solvents from buffer reservoirs containing: water (A) and 1.25M-$(NH_4)_2HPO_4$, adjusted to pH 3.8 with H_3PO_4 (B). Solvents were mixed according to the following gradient: time (min), %B; 0,0; 5,0; 65,100; 75,100. Additional separations were performed on a 25 cm x 4.6 mm Alltech Adsorbosphere SAX column (Grace Davison Discovery Sciences, Deerfield, IL, USA) fitted with a Alltech guard cartridge. The column was eluted at 1 mL/min with a gradient formed by mixing solvents from buffer reservoirs containing: water (A) and 1.25M-$(NH_4)_2HPO_4$, adjusted to pH 3.8 with H_3PO_4 (B). Solvents were mixed according to the following gradient: time (min), %B; 0,0; 5,0; 65,25; 67,0. Radioactivity was estimated by Cerenkov counting with a Canberra Packard (Pangbourne, Berks, UK) A500 series Radiomatic Flo Detector fitted with a 0.5 mL flow cell. The integration interval for counting was 12 s. Data was exported from the FloOne for Windows software (Canberra Packard) as an ASCII file and redrawn in Delta Graph v.4.0 (DeltaPoint, Monterey, CA). In some experiments fractions (0.2 or 0.3 min) were collected and radioactivity estimated by Cerenkov counting in a scintillation counter. Subsequently, 1mL water and 4mL Optima Flo-AP (Canberra Packard) scintillation fluid were added and the fractions were counted for ^{3}H or ^{14}C by scintillation counting in either a Wallac (Turku, Finland) 1409 or LabLogic (Sheffield, UK) 300SL scintillation counter.

Inositol phosphates

Myo-inositol(1,[^{32}P]2,3,4,5,6)P_6 was prepared using recombinant AtIPK1 according to Nagy et al. (Nagy et al, 2009). *Myo*-[2-^{3}H]inositol-l and *myo*-[^{14}C]inositol-labelled $InsP_5$ standards were prepared by acid treatment of the respective Ins(1,3,4,5,6)P_5 according to Brearley and Hanke (Brearley & Hanke, 1996).

Assays with Ins(1,[^{32}P]2,3,4,5,6)P_6 substrate

BtMinpp was assayed at 37 °C by the addition of 10 μL of 0.44 mg/mL protein in 20 mM glycine-HCl, pH 2.5, to 1 mL of 10 mM sodium salt of $InsP_6$ (Sigma) containing approximately 74 kBq Ins(1,[^{32}P]2,3,4,5,6)P_6. Aliquots of the assay were removed at intervals and subjected to chromatography. The data shown in Figure S3C was obtained after 60 min. Human MINPP1 was assayed at a protein concentration of 0.1 mg/mL in 25 mM Tris, 150 mM NaCl, 2mM $MgCl_2$, pH 7.2, with approximately 3.7 kBq of Ins(1,[^{32}P]2, 3,4,5,6)P_6. Reaction products were analyzed after 2 hours at 37 °C (Figure S3A). *Aspergillus ficuum* phytase (Sigma) was assayed at 37 °C by the addition of 10 μL of 0.44 mg/mL protein in 20 mM glycine-HCl, pH 2.5, to 1 mL of 10 mM sodium salt of $InsP_6$ (Sigma) containing approximately 7.4 kBq Ins(1,[^{32}P]2,3,4,5,6)P_6. The data shown in Figure S3B were obtained after 60 min. Assays of BtMinpp mutants were performed, beside native protein, at 37 °C by the addition of 1 μL of 1 mg/mL protein to 0.4 mL of 10 mM sodium salt of $InsP_6$ (Sigma) in 50 mM glycine-HCl, pH 3.5, containing approximately 7.4 kBq Ins(1,[^{32}P]2,3,4,5,6)P_6. The data shown in Figure 1 were obtained after 2 min. Reactions were stopped by boiling for 2 min, before addition of nucleotide standards (to monitor chromatography) and radiolabelled standards (to confirm identity of $InsP_5$ peaks).

Assay of BtMinpp with Ins(1,3,4,5)P_4 substrate

BtMinpp (2.5 ng/mL) was incubated for 30 min at 37 ^{0}C with 10 μM [^{3}H]Ins(1,3,4,5)P_4 in assay buffer containing 25 mM HEPES, 50 mM KCl. Assays were quenched and neutralized and analyzed by HPLC as described previously (Craxton et al, 1997).

Crystal structure determination

Recombinant BtMinpp was purified prior to crystallization by gel filtration using a Superdex 75 16/60 column and concentrated to 1 mg/ml. Crystallization proceeded by vapour diffusion using a 1:1 mixture of BtMinpp and a precipitant containing 0.2 M ammonium acetate pH 5.0, 18 % (w/v) PEG 3350. This crystal form, of space group $P2_1$, has a solvent content of 48 % and has 2 molecules of Minpp in the asymmetric unit. A native dataset was collected on beamline I-02 of the Diamond Light

Source (DLS) (Didcot, UK) from a single crystal at 100 K cryoprotected with 25 % (v/v) ethylene glycol. Crystals of selenomethionyl-derivitized BtMinpp were obtained by the same procedure except that the precipitant employed for crystallization contained 0.2 M imidazole-malate pH 6.0, 15 % (w/v) PEG 4000. A single crystal was used to collect a SAD (single-wavelength anomalous dispersion) dataset on beamline I-24 of the DLS at the Se K edge (λ = 0.9799 Å) to a resolution of 2.50 Å. Datasets were processed using MOSFLM (Leslie, 1999) and SCALA (Evans, 2006) as part of the CCP4 package (Collaborative Computational Project, 1994). The structure of SeMet Minpp was determined by SAD phasing using SHELX (Sheldrick, 2008) and chain-traced with BUCCANEER (Cowtan, 2006). This interim structural model was transferred to the cell of the native protein and refined against the native protein structure factors using PHENIX (Adams et al, 2010), alternating with manual rebuilding in COOT (Emsley & Cowtan, 2004). Refinement converged to give a final model for native Minpp displaying R-work and R-free values of 16.6 % and 21.3 %, respectively (Figure S4). When analyzed for stereochemical quality using MOLPROBITY (Chen et al, 2010) the final structure has only 2 residues (Ala 220 in both molecular copies in the AU) in the disallowed region of the Ramachadran plot. Following refinement this structure was found to contain a single inorganic phosphate ion bound at the active site. To produce crystals of substrate analogue-bound enzyme, phosphate-bound crystals were soaked in mother liquor solution containing 1 mM *myo*-inositol hexakis (hydrogen sulfate) hexapotassium salt ($InsS_6$) and 25 % (v/v) ethylene glycol. Collection of X-ray diffraction data followed on beamline I-24 of the DLS giving a dataset to a resolution of 2.42 Å. The structure of the $InsS_6$-bound complex was solved and refined to give R-work and R-free values of 15.6 % and 21.7 %, respectively. When analyzed for stereochemical quality using MOLPROBITY (Chen et al, 2010), the $InsS_6$-bound structure again has only residue Ala 220 in both molecular copies in the disallowed region of the Ramachandran plot. Full data collection, phasing and refinement statistics for all structures reported herein are presented in Table S3III.

Molecular docking calculations

Molecular docking experiments using torsionally-flexible phytate as ligand and the crystal structures of BtMinpp and *A.niger* PhyA (Kostrewa et al, 1997) as receptors were carried out using AutoDock

Vina (Trott & Olson, 2010). A D-2 axial model for $InsP_6$ (D-2 axial and five equatorial phosphates) was used as representative of the predominant conformation at the acidic pH used in our hydrolysis assays (Isbrandt & Oertel, 1980). Atomic coordinates for the ligand obtained from the Hic-Up database (Kleywegt et al, 2003). The structures of ligand and receptor were formatted with AutoDockTools 1.5.4 (Morris et al, 2009). The chemical bonds involving the phosphate groups of $InsP_6$ were defined as rotatable giving a total of 12 degrees of torsional freedom. Fixed (i.e. inflexible) enzyme models were used. A search space of 16 × 16 × 16 $Å^3$ was used, centred on and encompassing each enzyme active site. Docking poses were visualized with PyMOL (The PyMOL Molecular Graphics System, Version 1.3, Schrödinger, LLC). Productive binding modes were assigned as those poses found with (i) binding energies within 1 $kcalmol^{-1}$ of the global energy minimum and (ii) a His59 imidazole Nε2 atom to phosphate phosphorous distance of 4 Å or less. These were classified according to the phosphate groups bound in the S3 and S2 specificity subsites (Table SIV).

Site-directed mutagenesis

The A31Y and R183D BtMinpp protein variants were generated using the PCR-based Phusion Site-Directed Mutagenesis kit (Thermo Scientific) according to the manufacturer's instructions. The primer pairs A31Y (GAAGTATGCAGGGACGTACATGCCCTATCCTAATAG), A31Y_antisense (TGTATCTTAGTTTGCATATGGCTGCCG) and R183D (CAATATAATCATATCCTTGATTTTTTTGATCTGAATAAATC), R183D_antisense (TTTTCCTTCACTTCGCTGTAC) were used to obtain the A31Y and R183D amino acid substitutions, respectively, using pGH07 as a template. The resulting plasmids were used to transform Rosetta2(DE3)pLysS *E. coli* cells and protein expression and purification were carried out as described above.

Comparison of the structure of BtMinpp with other Branch 2 Histidine Phosphatases

The refined crystal structure of BtMinpp was compared to a representative subset of the available branch 2 histidine phosphates of known structure (Rigden, 2008). Dali-Lite (Holm & Park, 2000) was used to perform the structural alignments. Residue identities are calculated as the percentage of

residues found to be structurally-equivalent and chemically identical in the alignment. RMSDs are based on structurally-equivalent alpha-carbon atom positions. The protein structures with the following PDB codes were included: 1. Phytase PhyA from *A.fumigatus* (AfPhyA), PDB entry 1SK8, Uniprot sequence entry O000092 (Liu et al, 2004); 2. Phytase PhyA from *A.niger* (AnPhyA), 3K4Q, P34752 (Oakley, 2010); 3. Phytase PhyB from *A.niger* (AnPhyB), 1QFX, P34755 (Kostrewa et al, 1999); 4. Phytase from *Debaryomyces castellii* (DcPhyt), 2GFI, A2TBB4 (Ragon et al, 2009); 5. Phytase AppA from *E.coli* (EcAppA), 1DKL, P07102 (Lim et al, 2000); 6. Glucose-1-phosphatase from *E.coli* (EcG1Pase), 1NT4, P19926 (Lee et al, 2003); 7. Histidine acid phosphatase from *Francisella tularensis* (FtHAP), 3IT1, Q2A612 (Singh et al, 2009); 8. Phytase from *Hafnia Alvei* (HfPhyA), 4ARO, H9TUK5 (Ariza et al, 2013); 9. Human prostatic acid phosphatase (HsPAP), 1CVI, P15309 (Jakob et al, 2000); 10. Rat prostatic acid phosphatase (RnPAP), 1RPA, P20646 (Lindqvist et al, 1993).

Construction of *minpp* deletion mutant

A 755 bp chromosomal DNA fragment downstream from *minpp*, including the last 196 nucleotides within the C-terminal region, was amplified by PCR and cloned into the *Sph*I/*Sal*I sites of the pBluescript SK- vector into *E. coli* GC10 cells (Sigma). Subsequently, a 2.2-kb PCR-generated *Hind*III/*Pst*I fragment containing the tetracycline resistance gene *tetQ* from the *Bacteroides* plasmid pBT-2 (Tancula et al, 1992) was cloned into the *Hind*III/*Pst*I sites of the resulting plasmid, upstream from the 5'-truncated *minpp* gene. A *Sal*I/*Sac*I fragment containing the tetQ and 'minpp genes was cloned into the *E. coli-Bacteroides* shuttle suicide vector pFD516 (Smith et al, 1995). Finally, a 783 bp chromosomal DNA fragment upstream from *minpp*, including the first 244 nucleotides within the N-terminal region, was amplified by PCR and cloned into the *Sph*I/*Sal*I sites of *the* resulting pFD516-based plasmid. The new plasmid pGH051 containing the Δ*minpp*::*tetQ* construct, was mobilized from *E. coli* GC10 into *B. thetaiotaomicron* by triparental filter mating protocols (Shoemaker et al, 1986) using *E. coli* HB101(pRK2013) as the helper strain. Transconjugants were selected on BHI-hemin agar containing gentamicin and tetracycline. Determination of sensitivity to either tetracycline or erythromycin was carried out to identify recombinants that were tetracycline resistant and

erythromycin sensitive. PCR analysis and sequencing were used to confirm the double-crossover genetic allele exchange. A transconjugant, GH59, containing the Δ*minpp*::*tetQ* construct inserted into the *B. thetaiotaomicron* chromosome was selected for further studies.

Complementation of the *minpp* deletion mutant and overexpression

To complement the *B. thetaiotaomicron minpp* deletion mutant GH59, the *Bacteroides* expression vector pFI2716 (Wegmann et al, 2013) was employed. The primer pair *Bsp*HI_minPP1_5' (ATTCATGAAAAGATTATTATTTGTT) and minPP1_*Bam*HI_3' (TAGGATCCTATTCATTGAAAACTGA) was used to amplify a 1279 bp region encoding *minpp* from *B. thetaiotaomicron* VPI-5482 genomic DNA. The *minpp* fragment was digested with *Bsp*HI and *Bam*HI before cloning into the *Nco*I/*Bam*HI sites of the *Bacteroides* expression vector pFI2716, creating pGH035. Conversion of tetracycline resistance to clindamycin-erythromycin resistance: the primer pair ccr_amont2 (CATGCATATGAGCTCCATGCTATAGCTACC) and ccr_aval2 (CATGGGATCCGCCAGCCGTTATGCGGCAGC) was used to amplify a 1252 bp region encoding *ermF* from the plasmid pFD516 (Smith et al, 1995). The *ermF* fragment was digested with *Nde*I and cloned into *Nde*I digested (blunted) and *Nsi*I digested pGH035, to replace the existing 1803 bp *tetQ* portion of pGH035, creating pGH038. For overexpression purposes the *Bsp*HI and *Bam*HI digested minpp fragment described above was cloned into similar sites of the medium-level expression vector pGH020 (Wegmann et al, 2013) creating pGH036. This was followed by the conversion from tetracycline resistance to erythromycin resistance as described above, creating pGH037. The transformation of the *minpp* deletion mutant GH59 with pGH037 resulted in the creation GH115.

Periplasmic protein extraction

Bacteroides species were grown in 20 mL of BHI supplemented with 0.001% hemin for 16 hours. The cells were centrifuged at 3500 g for 10 minutes and the periplasmic fraction was prepared according to the method described by Osborn *et al*. (Osborn et al, 1972). Briefly, the cell pellet was resuspended in 4 ml of fractionation buffer (Tris 30 mM, sucrose 20%, EDTA 1mM, pH8) and incubated for 10 minutes at 20°C. The cell suspension was centrifuged for 10 minutes at 3000 g and the cell pellet was

resuspended in 0.8 ml of ice-cold 5 mM $MgSO_4$ and the suspension was left on ice for 10 minutes. The osmotic shock fluid was harvested by centrifugation for 10 minutes at 3000 g, 4°C. To verify that no cross-contamination of cytoplasmic proteins occurred, glucose-6-phosphate dehydrogenase activity was assessed (described in *S1 Materials and Methods*) in the different periplasmic fractions and no activity was detected.

Glucose-6-phosphate dehydrogenase assay

100 µl of periplasmic or cytoplasmic extract was added to the following solution, pre-incubated at 30°C: 2.5 mL of 50 mM glycylglycine buffer pH 7.4, 0.2 mL of 150 mM $MgCl_{2,}$; 0.1 mL of 20 mM NADP and 0.1 mL of 60 mM glucose-6-phosphate (Noltmann et al, 1961). The increase in A_{340}/min for 4 to 5 minutes was recorded (UVIKON XS spectrophotometer, NorthStar Scientific) and the A_{340}/minute from the initial linear portion of the curve was calculated.

Outer membrane vesicles protein extraction

Cultures of bacteria were centrifuged at 5000 g for 15 minutes at 4ºC and the supernatant filtered through a 0.22 µm PES membrane (Sartorius) to remove debris and cells. Supernatants were concentrated by molecular weight (100 kDa MWCO, Sartorius) and the retentate ultracentrifuged (150,000 g for 2 h at 4 °C in a Ti70 rotor (Beckman Instruments). The supernatant was carefully aspirated from the tubes and the vesicle pellet was resuspended with 25 mM Tris buffer (pH 7.4). The pellet was washed with the same Tris buffer and centrifuged at 16000 g for 30 min. The pellet was resuspended in Tris buffer and OMV sterility examined by checking for growth of any contaminating bacterial cells on BHIH agar. The OMVs were disrupted by sonication. The OMV protein content was determined using the Total Protein Micro protein assay reagent kit (Sigma).

$InsP_6$ phosphatase activity measurement

Contaminating inorganic phosphate was removed from *Bacteroides* cytoplasmic, periplasmic and OMV fractions using PiBind™ resin (Innova Biosciences) prior to $InsP_6$ phosphatase activity measurements. The phosphatase activity was measured after diluting 10 µl of phosphate-depleted

extracts with Tris buffer (25mM, pH 7.5) in the presence of 10 μM of $InsP_6$ (Merck) and the mixture was incubated at 37ºC for 1 hour. $InsP_6$ dephosphorylation was measured using a molybdate/malachite green-based activity assay for the quantification of phosphate release (PiColorLock Gold Phosphate Detection System, Innova Biociences) according to the manufacturer's instructions.

Measurement of Minpp activity in the extracellular medium

20 mL of *Bacteroides* 16-hour cultures in BHI-hemin were centrifuged at 3500 g for 10 minutes and the supernatants were filtered through a 0.22 μm PES membrane (Sartorius) to remove debris and cells. The supernatants were then filtered through a 1000 kDa MWCO (Sartorius) filter to remove OMVs and the filtrate was further filtrated through a 10 kDa MWCO filter (Sartorius) to concentrate the 20 mL solution containing Minpp (49 kDa) down to 200 μl (100x). $InsP_6$ phosphatase activity was measured following depletion of inorganic phosphate with PiBind™ resin (Innova Biosciences) in 10 μl of the concentrated solution using the malachite green activity assay.

Measurement of the catalytic activity of BtMinpp in resuspended OMVs

Bacterial cells from 500 mL cultures were centrifuged at 5000 g for 20 minutes at 4ºC and the supernatant filtered through a 0.22 μm PES membrane (Sartorius) to remove debris and cells. Supernatants were concentrated by molecular weight (100 kDa MWCO) using a Vivaflow 50 ultrafiltration device (Sartorius) at 4ºC. To rinse the vesicles, the 5 mL of residual retentate was diluted 100 times in Tris buffer (25mM, pH 7.5) and the resulting 500 mL concentrated to 5 mL. The vesicles were further concentrated to 2 mL with a Vivaspin 20 centrifugal concentrator (Sartorius) and filtered through a 0.22 μm PES membrane (Sartorius). Vesicles concentrations were adjusted according to the protein concentration obtained after sonication of the suspensions using the Bradford method. To measure the activity of BtMinpp, 5 μL of vesicle suspension (corresponding to 5 μg of soluble protein) was added to 70 μL of either Tris buffer (25mM, pH 7.5) or in glycine-HCl buffer (20 mM, pH 2.5) and the PiColorLock Gold Phosphate Detection System was used. For *ex vivo* $InsP_6$ degradation experiments, caecal contents from 4 C57BL/6 mice were homogenised in distilled water,

centrifuged at 10,000 g for 5 min and the pellet was washed once with distilled water. Supernatants at each washing step were collected and the volume was adjusted to a final dilution factor of 1:10 (wt:v). To remove microorganisms, the supernatants were filtered through a 0.22 µm PES membrane (Sartorius). The pH of the extracts was measured at 6.5. 10 µl of vesicle suspension was added to 90 µl of extract to which 1mM of $InsP_6$ was added. HPLC: Inositol phosphates were resolved by anion exchange chromatography on a Dionex CarboPac PA-200 column (3mm x 250mm) with guard column (3mm x 50mm) eluted at a flow rate of 0.4 $ml.min^{-1}$ with a gradient of methane sulfonic acid prepared from buffer reservoirs containing water (A) and 0.6M methane sulfonic acid (B) mixed in ratio: time (min), % B; 0,10; 22,60; 25,100; 38,100; 39,10; 49,10. Inositol phosphates were detected by absorbance measurement at 290nm after post-column addition at 0.2 ml. min^{-1}, of 0.1% (w/v) ferric nitrate in 2% (w/v) perchloric acid using a 375 µl knitted reaction coil.

References

Adams PD, Afonine PV, Bunkoczi G, Chen VB, Davis IW, Echols N, Headd JJ, Hung LW, Kapral GJ, Grosse-Kunstleve RW, McCoy AJ, Moriarty NW, Oeffner R, Read RJ, Richardson DC, Richardson JS, Terwilliger TC, Zwart PH (2010) PHENIX: a comprehensive Python-based system for macromolecular structure solution. *Acta Crystallogr D* 66: 213-221

Ariza A, Moroz OV, Blagova EV, Turkenburg JP, Waterman J, Roberts SM, Vind J, Sjoholm C, Lassen SF, De Maria L, Glitsoe V, Skov LK, Wilson KS (2013) Degradation of phytate by the 6-phytase from Hafnia alvei: a combined structural and solution study. *PloS one* 8: e65062

Brearley CA, Hanke DE (1996) Metabolic evidence for the order of addition of individual phosphate esters to the myo-inositol moiety of inositol hexakisphosphate in the duckweed Spirodela polyrhiza L. *Biochem J* 314: 227-233

Caffrey JJ, Hidaka K, Matsuda M, Hirata M, Shears SB (1999) The human and rat forms of multiple inositol polyphosphate phosphatase: functional homology with a histidine acid phosphatase up-regulated during endochondral ossification. *Febs Lett* 442: 99-104

Chassard C, Delmas E, Lawson PA, Bernalier-Donadille A (2008) Bacteroides xylanisolvens sp nov., a xylan-degrading bacterium isolated from human faeces. *Int J Syst Evol Micr* 58: 1008-1013

Chen VB, Arendall WB, Headd JJ, Keedy DA, Immormino RM, Kapral GJ, Murray LW, Richardson JS, Richardson DC (2010) MolProbity: all-atom structure validation for macromolecular crystallography. *Acta Crystallogr D* 66: 12-21

Collaborative Computational Project N (1994) The CCP4 suite: Programs for protein crystallography. *Acta Crystallogr D Biol Crystallogr* 50: 760-763

Cowtan K (2006) The Buccaneer software for automated model building. 1. Tracing protein chains. *Acta Crystallogr D* 62: 1002-1011

Craxton A, Caffrey JJ, Burkhart W, Safrany ST, Shears SB (1997) Molecular cloning and expression of a rat hepatic multiple inositol polyphosphate phosphatase. *Biochem J* 328: 75-81

Dassa E, Boquet PL (1985) Identification of the Gene Appa for the Acid-Phosphatase (Ph Optimum 2.5) of Escherichia-Coli. *Mol Gen Genet* 200: 68-73

Ehrlich KC, Montalbano BG, Mullaney EJ, Dischinger HC, Jr., Ullah AH (1993) Identification and cloning of a second phytase gene (phyB) from Aspergillus niger (ficuum). *Biochemical and biophysical research communications* 195: 53-57

Emsley P, Cowtan K (2004) Coot: model-building tools for molecular graphics. *Acta Crystallogr D* 60: 2126-2132

Evans P (2006) Scaling and assessment of data quality. *Acta Crystallogr D Biol Crystallogr* 62: 72-82

Holm L, Park J (2000) DaliLite workbench for protein structure comparison. *Bioinformatics* 16: 566-567

Isbrandt LR, Oertel RP (1980) Conformational States of Myoinositol Hexakis(Phosphate) in Aqueous-Solution - a C-13 Nmr, P-31 Nmr, and Raman-Spectroscopic Investigation. *J Am Chem Soc* 102: 3144-3148

Jakob CG, Lewinski K, Kuciel R, Ostrowski W, Lebioda L (2000) Crystal structure of human prostatic acid phosphatase. *The Prostate* 42: 211-218

Kleywegt GJ, Henrick K, Dodson EJ, van Aalten DMF (2003) Pound-wise but penny-foolish-How well do macromolecules fare in macromolecular refinement? *Structure* 11: 1051-1059

Kostrewa D, Leitch FG, DArcy A, Broger C, Mitchell D, vanLoon APGM (1997) Crystal structure of phytase from Aspergillus ficuum at 2.5 angstrom resolution. *Nat Struct Biol* 4: 185-190

Kostrewa D, Wyss M, D'Arcy A, van Loon APGM (1999) Crystal structure of Aspergillus niger pH 2.5 acid phosphatase at 2.4 angstrom resolution. *J Mol Biol* 288: 965-974

Lee DC, Cottrill MA, Forsberg CW, Jia Z (2003) Functional insights revealed by the crystal structures of Escherichia coli glucose-1-phosphatase. *J Biol Chem* 278: 31412-31418

Leslie AG (1999) Integration of macromolecular diffraction data. *Acta Crystallogr D Biol Crystallogr* 55: 1696-1702

Lim D, Golovan S, Forsberg CW, Jia Z (2000) Crystal structures of Escherichia coli phytase and its complex with phytate. *Nat Struct Biol* 7: 108-113

Lindqvist Y, Schneider G, Vihko P (1993) Three-dimensional structure of rat acid phosphatase in complex with L(+)-tartrate. *J Biol Chem* 268: 20744-20746

Liu Q, Huang Q, Lei XG, Hao Q (2004) Crystallographic snapshots of Aspergillus fumigatus phytase, revealing its enzymatic dynamics. *Structure* 12: 1575-1583

Morris GM, Huey R, Lindstrom W, Sanner MF, Belew RK, Goodsell DS, Olson AJ (2009) AutoDock4 and AutoDockTools4: Automated Docking with Selective Receptor Flexibility. *J Comput Chem* 30: 2785-2791

Nagy R, Grob H, Weder B, Green P, Klein M, Frelet-Barrand A, Schjoerring JK, Brearley C, Martinoia E (2009) The Arabidopsis ATP-binding Cassette Protein AtMRP5/AtABCC5 Is a High Affinity Inositol Hexakisphosphate Transporter Involved in Guard Cell Signaling and Phytate Storage. *J Biol Chem* 284: 33614-33622

Noltmann E, Gubler CJ, Kuby SA (1961) Glucose 6-Phosphate Dehydrogenase (Zwischenferment) .1. Isolation of Crystalline Enzyme from Yeast. *J Biol Chem* 236: 1225-&

Notredame C, Higgins DG, Heringa J (2000) T-Coffee: A novel method for fast and accurate multiple sequence alignment. *J Mol Biol* 302: 205-217

Oakley AJ (2010) The structure of *Aspergillus nige*r phytase PhyA in complex with a phytate mimetic. *Biochem Biophys Res Comm* 397: 745-749

Osborn MJ, Gander JE, Parisi E, Carson J (1972) Mechanism of Assembly of Outer Membrane of Salmonella-Typhimurium - Isolation and Characterization of Cytoplasmic and Outer Membrane. *J Biol Chem* 247: 3962-&

Ragon M, Hoh F, Aumelas A, Chiche L, Moulin G, Boze H (2009) Structure of Debaryomyces castellii CBS 2923 phytase. *Acta Crystallogr F* 65: 321-326

Rigden DJ (2008) The histidine phosphatase superfamily: structure and function. *Biochem J* 409: 333-348

Rodriguez E, Han YM, Lei XG (1999) Cloning, sequencing, and expression of an Escherichia coli acid phosphatase/phytase gene (appA2) isolated from pig colon. *Biochemical and biophysical research communications* 257: 117-123

Romano PR, Wang J, O'Keefe RJ, Puzas JE, Rosier RN, Reynolds PR (1998) HiPER1, a phosphatase of the endoplasmic reticulum with a role in chondrocyte maturation. *J Cell Sci* 111: 803-813

Sambrook J, Russel DW (2001) *Molecular cloning: A laboratory Manual*, New York, USA: Cold Spring Harbor Press.

Sheldrick GM (2008) SHELXL97 and SHELXS97, programs for crystal structure solution and refinement. *Acta Crystallogr* 64: 112-122

Shoemaker NB, Getty C, Gardner JF, Salyers AA (1986) Tn4351 Transposes in Bacteroides Spp and Mediates the Integration of Plasmid R751 into the Bacteroides Chromosome. *J Bacteriol* 165: 929-936

Singh H, Felts RL, Schuermann JP, Reilly TJ, Tanner JJ (2009) Crystal Structures of the histidine acid phosphatase from Francisella tularensis provide insight into substrate recognition. *J Mol Biol* 394: 893-904

Smith CJ, Rollins LA, Parker AC (1995) Nucleotide sequence determination and genetic analysis of the Bacteroides plasmid, pBI143. *Plasmid* 34: 211-222

Tancula E, Feldhaus MJ, Bedzyk LA, Salyers AA (1992) Location and Characterization of Genes Involved in Binding of Starch to the Surface of Bacteroides-Thetaiotaomicron. *J Bacteriol* 174: 5609-5616

Trott O, Olson AJ (2010) Software News and Update AutoDock Vina: Improving the Speed and Accuracy of Docking with a New Scoring Function, Efficient Optimization, and Multithreading. *J Comput Chem* 31: 455-461

Ullah AH, Dischinger HC, Jr. (1993) Aspergillus ficuum phytase: complete primary structure elucidation by chemical sequencing. *Biochemical and biophysical research communications* 192: 747-753

van Hartingsveldt W, van Zeijl CM, Harteveld GM, Gouka RJ, Suykerbuyk ME, Luiten RG, van Paridon PA, Selten GC, Veenstra AE, van Gorcom RF, et al. (1993) Cloning, characterization and overexpression of the phytase-encoding gene (phyA) of Aspergillus niger. *Gene* 127: 87-94

Wegmann U, Horn N, Carding SR (2013) Defining the Bacteroides Ribosomal Binding Site. *Appl Environ Microb* 79: 1980-1989

Figure S1. The products of BtMinpp activity against $InsP_6$ are distinct from the products of attack of fungal phytase. Phytase and Minpps were incubated with *myo*-inositol(1,[^{32}P]2,3,4,5,6)P_6 and the products resolved on a Partisphere SAX HPLC column eluted with a gradient of $(NH_4)_2HPO_4$. human MINPP1 (A), *Aspergillus ficuum* phytase (Sigma) (B), recombinant *B. thetaiotaomicron* BtMinpp (C). The traces also show the elution of internal standards of AMP, ADP and ATP monitored at a wavelength of 259nm (grey line), and radioactivity monitored on-line by Cerenkov counting (black line). The regions of the chromatogram in which $InsP_2$, $InsP_3$ and $InsP_4$ isomers elute are indicated in (A). The Ins(1,[^{32}P]2,3,4,5,6)P_6 preparation used in (A) contained residual [^{32}P]ATP.

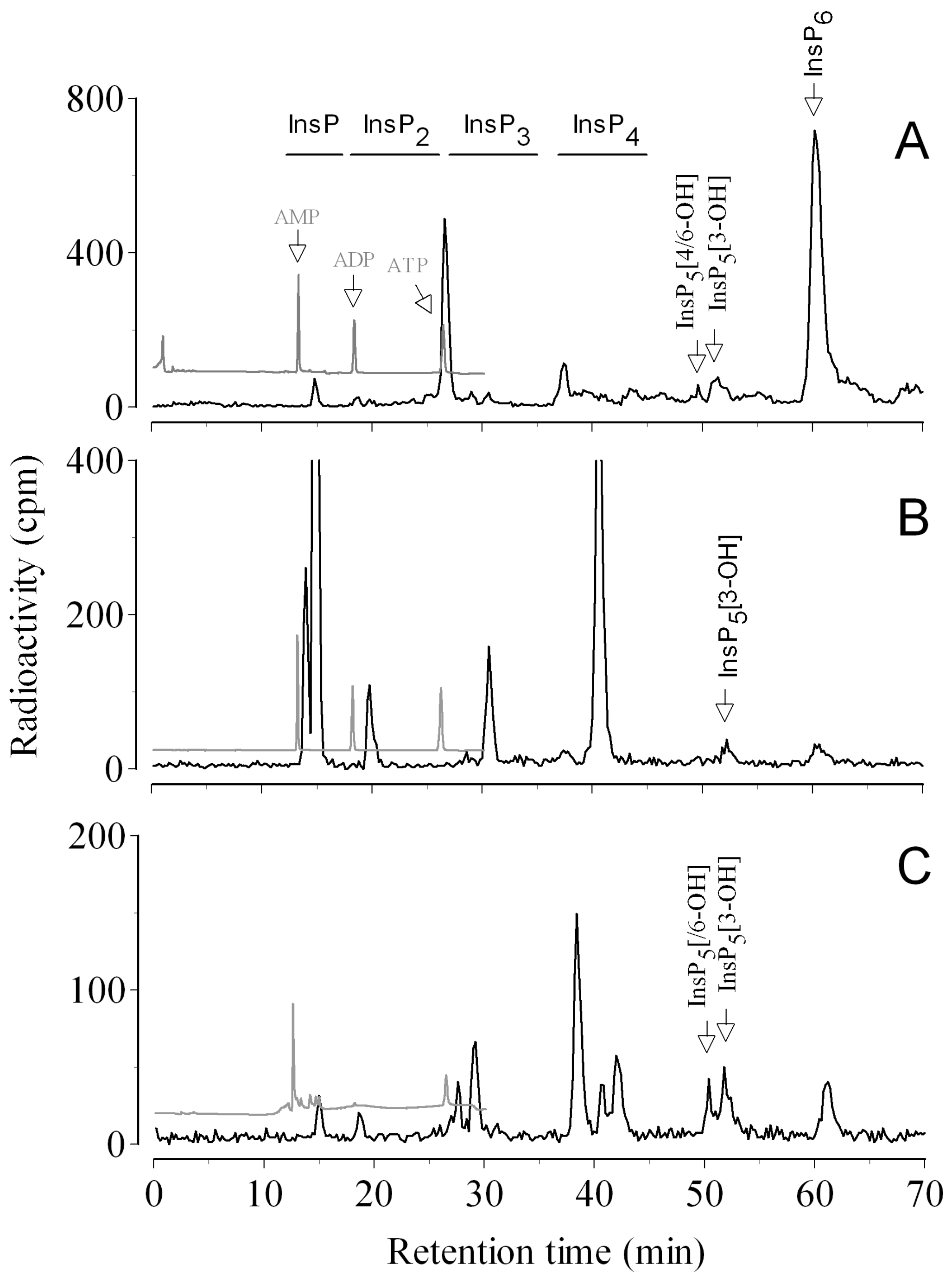

Figure S2. Products of hydrolysis of Ins(1,3,4,5)P_4 and InsP_6. (A) Native BtMinpp was incubated with [3H]Ins(1,3,4,5)P_4 at pH 7.0. The products were mixed with [14C]Ins(1,3,4)P_3 and resolved by HPLC. Fractions were collected and radioactivity estimated by scintillation counting: 3H, open circles; 14C, filled circles. The position of elution of Ins(1,4,5)P_3, revealed in parallel HPLC runs, is also shown. (B) Native BtMinpp was incubated with *myo*-inositol(1,[32P]2,3,4,5,6)P_6. Products were resolved by Partisphere SAX HPLC and detected by on-line Cerenkov counting. The regions of the chromatogram in which InsP_3 and InsP_4 isomers elute are indicated. The peak eluting at approximately 15 min was present in the preparation of *myo*-inositol(1,[32P]2,3,4,5,6)P_6.

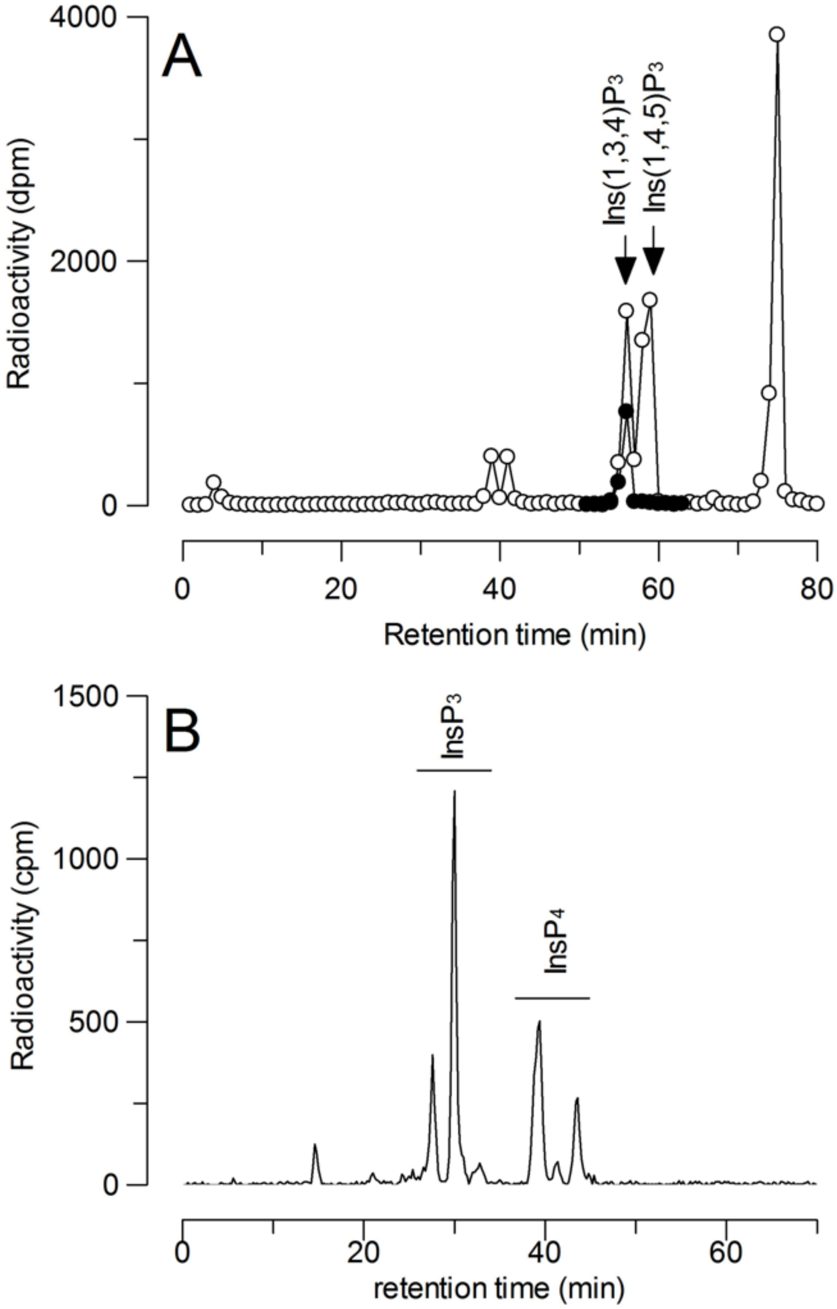

Figure S3. Relative $InsP_6$ phosphatase activity (%) of recombinant BtMinpp at different pH values

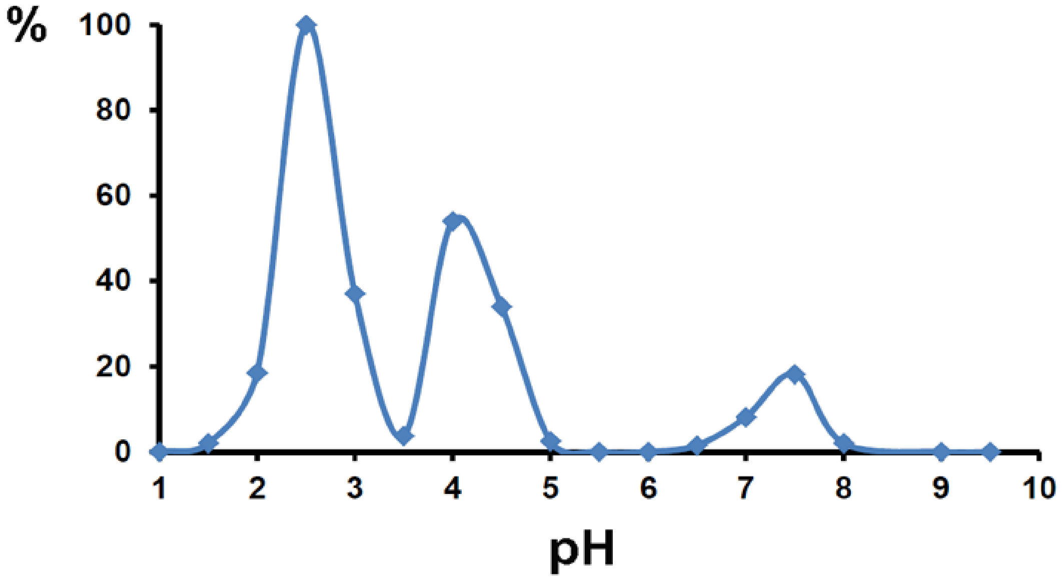

Figure S4. Active site electron density for the complex of BtMinpp with inorganic phosphate. A region of the Sigma-A weighted double difference Fourier electron density map contoured at 1.1 σ for the residues of the fingerprint RHGXRXP (58-64) and HAE (323-325) residues of the active site of the complex of BtMinpp with inorganic phosphate. Active site residues and phosphate group are shown as sticks.

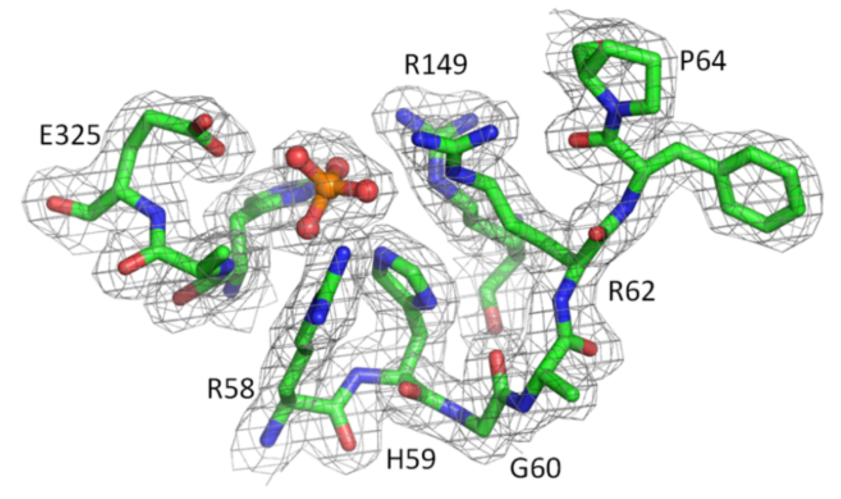

Figure S5. A T-coffee (Notredame et al, 2000) alignment of the sequences of BtMinpp and selected MINPPs. Sequences are: BtMinpp – this study, sequence database entry Q89YI8; Human – Human multiple inositol polyphosphate histidine phosphatase 1, Q9UNW1; Rat - Rat multiple inositol polyphosphate histidine phosphatase 1 O35217; Chicken - Histidine phosphatase of the endoplasmic reticulum from chicken, Q92170. Residue numbering follows that of BtMinpp. Absolutely conserved residues are shown in red boxes, conserved residues are boxed in blue. Secondary structural elements of BtMinpp are shown, labelled to reflect homology with the corresponding elements in the structure of *A. niger* PhyA (Oakley, 2010). Blue triangles indicate residues which make direct contact with the $InsS_6$ ligand in its complex with BtMinpp, whilst green triangles show those additional residues which have an atom within 8 Å of the ligand.

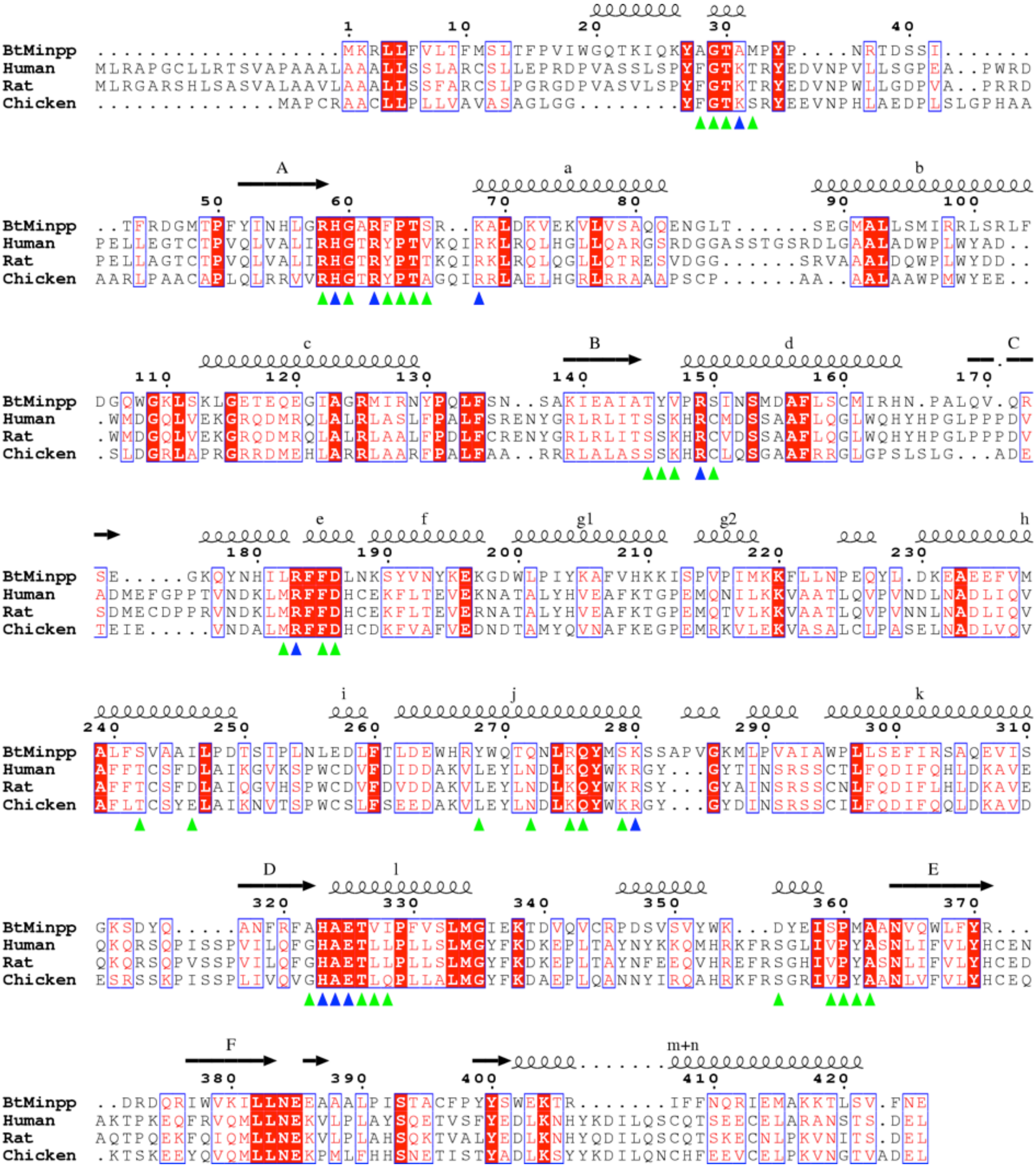
BtMinpp ....................MKRLLFVLTFMSLTFPVIWGQTKIQKYAGTAMPYP....NRTDSSI.......
Human MLRAPGCLLRTSVAPAAALAAALLSSLARCSLLEPRDPVASSLSPYFGTKTRYEDVNPVLLSGPEA..PWRD
Rat MLRGARSHLSASVALAAVLAAALLSSFARCSLPGRGDPVASVLSPYFGTKTRYEDVNPWLLGDPVA..PRRD
Chicken .............MAPCRAACLLPLLVAVASAGLGG........YFGTKSRYEEVNPHLAEDPLSLGPHAA
BtMinpp ..TFRDGMTPFYINHLGRHGARFPTSR..KALDKVEKVLVSAQQENGLT......SEGMALLSMIRRLSRLF
Human PELLEGTCTPVQLVALIRHGTRYPTVKQIRKLRQLHGLLQARGSRDGGASSTGSRDLGAALADWPLWYAD..
Rat PELLAGTCTPVQLVALIRHGTRYPTTKQIRKLRQLQGLLQTRESVDGG......SRVAAALDQWPLWYDD..
Chicken AARLPAACAPLQLRRVVRHGTRYPTAGQIRRLAELHGRLRRAAAPSCP......AA..AALAAWPMWYEE..
BtMinpp DGQWGKLSKLGETEQEGIAGRMIRNYPQLFSN..SAKIEAIATYVPRSINSMDAFLSCMIRHN.PALQV.QR
Human .WMDGQLVEKGRQDMRQLALRLASLFPALFSRENYGRLRLITSSKHRCMDSSAAFLQGLWQHYHPGLPPPDV
Rat .WMDGQLVEKGRQDMRQLALRLAALFPDLFCRENYGRLRLITSSKHRCVDSSAAFLQGLWQHYHPGLPPPDV
Chicken .SLDGRLAPRGRRDMEHLARRLAARFPALFAA..RRRLALASSSKHRCLQSGAAFRRGLGPSLSLG...ADE
BtMinpp SE.....GKQYNHILRFFDLNKSYVNYKEKGDWLPIYKAFVHKKISPVPIMKKFLLNPEQYL.DKEAEEFVM
Human ADMEFGPPTVNDKLMRFFDHCEKFLTEVEKNATALYHVEAFKTGPEMQNILKKVAATLQVPVNDLNADLIQV
Rat SDMECDPPRVNDKLMRFFDHCEKFLTEVERNATALYHVEAFKTGPEMQTVLKKVAATLQVPVNNLNADLIQV
Chicken TEIE.....VNDALMRFFDHCDKFVAFVEDNDTAMYQVNAFKEGPEMRKVLEKVASALCLPASELNADLVQV
BtMinpp ALFSVAAILPDTSIPLNLEDLFTLDEWHRYWQTQNLRQYMSKSSAPVGKMLPVAIAWPLLSEFIRSAQEVIS
Human AFFTCSFDLAIKGVKSPWCDVFDIDDAKVLEYLNDLKQYWKRGY...GYTINSRSSCTLFQDIFQHLDKAVE
Rat AFFTCSFDLAIQGVHSPWCDVFDVDDAKVLEYLNDLKQYWKRSY...GYAINSRSSCNLFQDIFLHLDKAVE
Chicken AFLTCSYELAIKNVTSPWCSLFSEEDAKVLEYLNDLKQYWKRGY...GYDINSRSSCILFQDIFQQLDKAVD
BtMinpp GKSDYQ.....ANFRFAHAETVIPFVSLMGIEKTDVQVCRPDSVSVYWK...DYEISPMAANVQWLFYR...
Human QKQRSQPISSPVILQFGHAETLLPLLSLMGYFKDKEPLTAYNYKKQMHRKFRSGLIVPYASNLIFVLYHCEN
Rat QKQRSQPVSSPVILQFGHAETLLPLLSLMGYFKDKEPLTAYNFEEQVHREFRSGHIVPYASNLIFVLYHCED
Chicken ESRSSKPISSPLIVQVGHAETLQPLLALMGYFKDAEPLQANNYIRQAHRKFRSGRIVPYAANLVFVLYHCEQ
BtMinpp ..DRDQRIWVKILLNEEAAALPISTACFPYYSWEKTR.......IFFNQRIEMAKKTLSV.FNE
Human AKTPKEQFRVQMLLNEKVLPLAYSQETVSFYEDLKNHYKDILQSCQTSEECELARANSTS.DEL
Rat AQTPQEKFQIQMLLNEKVLPLAHSQKTVALYEDLKNHYQDILQSCQTSKECNLPKVNITS.DEL
Chicken .KTSKEEYQVQMLLNEKPMLFHHSNETISTYADLKSYYKDILQNCHFEEVCELPKVNGTVADEL

Table S1 Alignment of BtMinpp protein sequence with known MINPPs and phytases*

Accession	Description	Coverage (%)	E value	Identity	Alignment scores
NP_813655	*B. thetaiotaomicron* BtMinpp	100	0	425/425 (100%)	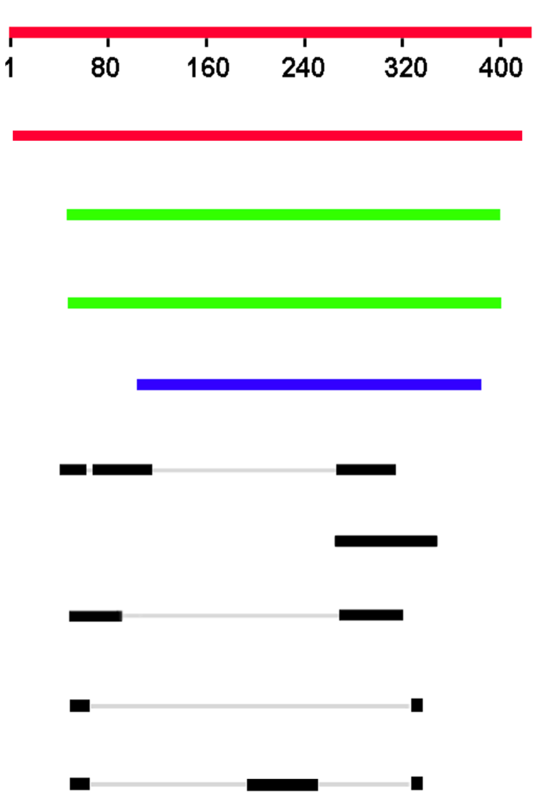
ZP_03475410	*Parabacteroides johnsonii* PRABACTJOHN_01069	97	0	257/416 (62%)	
NP_004888	Human MINPP1 (Caffrey et al, 1999)	82	2e-14	90/381 (23%)	
NP_062136	Rat MINPP1 (Craxton et al, 1997)	82	6e-15	81/377 (21%)	
NP_989975	Chicken HiPER1 (Romano et al, 1998)	65	5e-08	70/306 (23%)	
CAA78904	*Aspergillus niger* PhyA (van Hartingsveldt et al, 1993)	28	4e-05	32/83 (38%)	
AAA02934	*A. niger* PhyB (Ehrlich et al, 1993)	18	6e-04	21/84 (25%)	
AAB26466	*A. ficuum* phytase (Ullah & Dischinger, 1993)	19	2e-05	32/83 (38%)	
AAA72086	*Escherichia coli* AppA (Dassa & Boquet, 1985)	5	0.27	7/15 (47%)	
AAR87658	*E. Coli* AppA2 (Rodriguez et al, 1999)	17	0.18	21/70 (30%)	

*Similarities of BtMinpp to known proteins were determined using the BLASTP 2 sequences tool for local alignment. BtMinpp is shown on the top line of the Table so that its entire length can be displayed for comparative purposes. The colour key for the alignment scores (in bits) is: red, ≥ 200; green, 50-80; blue, 40-50; black, 18-40.

Table S2. Molecular and Enzymatic Properties of BtMinpp

Molecular mass	49 kDa, monomer
pH optimum	2.5, 4.0 and 7.5 (see Figure S1)
pH stability	Stable for 24 hours at pH between 2 and 5 at 4°C
Kinetic constants at pH 7.5 and 37°C	$\underline{InsP_6}$ K_M : 44.78 ± µM<br>V_{max} : 69.63 µmoles/min/mg<br>K_{cat} : 57.1 s^{-1}<br>$\underline{InsP_4}$ $K_{M\,:}$ 19.4 ± 8.9 µM<br>$V_{max\,:}$ 1423 ± 24 µmoles/min/mg<br>K_{cat} : 1167 s^{-1}
Kinetic constants at pH 2.5 and 37°C	$\underline{InsP_6}$ K_M : 18.4 ± 4.1 µM<br>V_{max} : 178 ± 32 µmoles/min/mg<br>K_{cat} : 146 s^{-1}<br>$\underline{InsP_4}$ K_M : 27.1 ± 7.2 µM<br>V_{max} : 6385 ± 732 µmoles/min/mg<br>K_{cat} : 5238 s^{-1}
Pepsin resistance	High; >95% of initial activity after 30min at 37°C with 3000U pepsin
Enzyme activity at low pH	pH 2.0: 18% of initial activity<br>pH 1.0: 1.6% of initial activity
Temperature optimum	55°C
Residual activity at high temperatures	70°C, 10 min: 1.26% of initial activity<br>80°C, 10 min: 0% of initial activity

Table S3. Statistics from the comparison of BtMinpp with the structures of branch 2 histidine phosphatases. The residue identity is calculated as the percentage of residues found to be structurally-equivalent and chemically identical in the alignment.

Protein	PDB entry	Uniprot entry	Z-score	Number of Equivalent residues	Residue identity (%)	RMSD (Å)
AfPhyA	1SK8	O00092	35.0	366	15	2.7
AnPhyA	3K4Q	P34752	34.5	360	16	2.5
AnPhyB	1QFX	P34755	32.6	362	14	3.2
DcPhyt	2GFI	A2TBB4	33.1	363	18	2.7
EcAppA	1DKL	P07102	23.9	308	13	4.1
EcG1Pase	1NT4	P19926	24.4	306	12	3.8
FtHAP	3IT1	O57942	25.1	291	14	3.6
HaPhyA	4ARO	H9TUK5	23.6	306	12	4.2
HsPAP	1CVI	P15309	25.5	300	13	3.6
RnPAP	1RPA	P20646	25.1	300	14	3.6

Table S4. Predicted low energy binding modes resulting from *in silico* docking of $InsP_6$ to enzyme structures. Columns show the identities of the phosphate groups of $InsP_6$ bound at either the S3 or S2 specificity subsites of BtMinpp and PhyA (*A.niger* phytase, PDB entry 3K4Q). The binding modes are indicated as either obverse (O) or reverse (R).

**Enzyme**	**S3 pocket**	**S2 pocket**	**Binding Mode**
BtMinpp	D-5	D-6	R
	D-6	D-1	R
	D-3	D-2	O
	D-6	D-5	O
PhyA	D-3	D-2	O

Table S5. Genes annotated as phytases in 341 microbial genomes isolated from the human gastrointestinal tract (from the Human Microbiome Project, http://www.hmpdacc-resources.org/cgi-bin/imgm_hmp/main.cgi)

Genome	Phylum	Locus Tag	Gene Product Name
Acidaminococcus sp. D21	Firmicutes	ACDG_00696	phytase
Oxalobacter formigenes OXCC13	Proteobacteria	OFBG_00876	4-phytase
Paenibacillus sp. HGF7	Firmicutes	HMPREF9413_5666	3-phytase
Providencia stuartii ATCC 25827	Proteobacteria	PstuA_020100004501	4-phytase

Table S6. Source organisms for MINPP proteins*

Common name	NCBI Reference Sequence	Scientific name
Acidovorax avenae	YP_004236708.1	*Acidovorax avenae subsp. avenae* ATCC 19860
Acinetobacter baumannii	YP_001846348.1	*Acinetobacter baumannii* ACICU
Acinetobacter calcoaceticus	ADY81607.1	*Acinetobacter calcoaceticus* PHEA-2
Acinetobacter sp. DR1	YP_003732360.1	*Acinetobacter sp.* DR1
Aeromonas veronii	YP_004393571.1	*Aeromonas veronii* B565
Bacteroides thetaiotaomicron	NP_813655.1	*Bacteroides thetaiotaomicron* VPI-5482
Bacteroides xylanisolvens	CBK68912.1	*Bacteroides xylanisolvens* XB1A
Bacteroides xylanisolvens	CBK66891.1	*Bacteroides xylanisolvens* XB1A
Bacteroides xylanisolvens	CBK68541.1	*Bacteroides xylanisolvens* XB1A
Bifidobacterium dentium	YP_003361382.1	*Bifidobacterium dentium* Bd1
Bifidobacterium longum	YP_002321769.1	*Bifidobacterium longum subsp. infantis* ATCC 15697
Bifidobacterium pseudocatenulatum	ZP_03743199.1	*Bifidobacterium pseudocatenulatum* DSM 20438
Bradyrhizobium sp. BTAi1	YP_001236730.1	*Bradyrhizobium sp. BTAi1*
Burkholderia ambifaria	ZP_02888054.1	*Burkholderia ambifaria* IOP40-10
Burkholderia cenocepacia	ZP_04942680.1	*Burkholderia cenocepacia* PC184
Burkholderia pseudomallei	ZP_01765847.1	*Burkholderia pseudomallei* 305
Burkholderia thailandensis	ZP_02466008.1	*Burkholderia thailandensis* MSMB43
Clavibacter michiganensis	YP_001221200.1	*Clavibacter michiganensis* subsp. *michiganensis* NCPPB 382
Enhydrobacter aerosaccus	ZP_05619011.1	*Enhydrobacter aerosaccus* SK60
Fusobacterium mortiferum	ZP_04568756.1	*Fusobacterium mortiferum* ATCC 9817
Marinomonas sp. MWYL1	YP_001339041.1	*Marinomonas sp.* MWYL1
Parabacteroides johnsonii	ZP_03475410.1	*Parabacteroides johnsonii* DSM 18315
Photobacterium leiognathi	ZP_08311993.1	*Photobacterium leiognathi* subsp. *mandapamensis* svers.1.1.
Rhodococcus erythropolis	YP_002763531.1	*Rhodococcus erythropolis* PR4
Spirochaeta smaragdinae	YP_003803564.1	*Spirochaeta smaragdinae* DSM 11293
Streptomyces hygroscopicus	ZP_07292879.1	*Streptomyces hygroscopicus* ATCC 53653
Variovorax paradoxus	YP_004156191.1	*Variovorax paradoxus* EPS
Arabidopsis thaliana	NP_563856.1	*Arabidopsis thaliana*
Barley	ABJ98329.1	*Hordeum vulgare*
Lily	ABD96177.1	*Lilium longiflorum*
Maize	NP_001149246.1	*Zea mays*
Physcomitrella patens	XP_001765102.1	*Physcomitrella patens subsp. patens*
Rice Indica	EAY92352.1	*Oryza sativa Indica Group*
Rice Japonica	NP_001051705.1	*Oryza sativa Japonica Group*
Selaginella moellendorffii	XP_002985961.1	*Selaginella moellendorffii*
Sorghum	XP_002466207.1	*Sorghum bicolor*
Wheat	ABJ98331.1	*Triticum aestivum*
Albugo laibachii	CCA15134.1	*Albugo laibachii Nc14*
Capsaspora owczarzaki	EFW47484.1	*Capsaspora owczarzaki ATCC 30864*
Dictyostelium discoideum	XP_638245.1	*Dictyostelium discoideum AX4*
Phytophthora infestans	XP_002907675.1	*Phytophthora infestans T30-4*
Chimpanzee	XP_507896.2	*Pan troglodytes*

Common marmoset	XP_003087945.1	*Callithrix jacchus*
Giant panda	XP_002756410.1	*Ailuropoda melanoleuca*
Gray short-tailed opossum	XP_001374318.1	*Monodelphis domestica*
Horse	XP_001503270.1	*Equus caballus*
Human	AAD02437.1	*Homo sapiens*
Macaque	XP_001101346.1	*Macaca mulatta*
Mouse	EDL41725.1	*Mus musculus*
Oikopleura dioica	CBY40869.1	*Oikopleura dioica*
Oxen	NP_001033664.2	*Bos taurus*
Rat	NP_062136.1	*Rattus norvegicus*
Sumatran orangutan	NP_001126173.1	*Pongo abelii*
Vase tunicate	XP_002121164.1	*Ciona intestinalis*
Western clawed frog	XP_002935472.1	*Xenopus (Silurana) tropicalis*
Wild boar	XP_001927707.1	*Sus scrofa*

*Animals, blue; Plants, green; Protists, salmon-pink; Bacteria, yellow.

Table S7. *Escherichia coli* and *Bacteroides* strains used in this study

Species	Strain	Relevant characteristics*	Source or reference
E. coli	HB101(pRK2013)	$Kana^r$, Contains RK2 transfer genes	DMSZ Collection
	GC10	Cloning strain	Sigma
	GC10(pGH051)	Tet^R	This work
	BL21-CodonPlus (DE3) RIL	Cm^R	Stratagene
	Rosetta™ 2(DE3)pLysS	Cm^R	Novagen
	B834 (DE3)		Novagen
	B834-CodonPlus (DE3) RIL	Cm^R, B834 (DE3) transformed with RIL plasmid	This work
	GH24	Amp^R Cm^R, *E. coli* BL21 (DE3) codon+ with pGH07	This work
	GH25	Amp^R Cm^R, *E. coli* B834 (DE3) codon+ with pGH07	This work
B. fragilis	NCTC 9343		DMSZ Collection
B. xylanisolvens	XB1A		(Chassard et al, 2008)
B. thetaiotomicron	VPI-5482		DMSZ Collection
B. thetaiotomicron	GH59	VPI-5482 with tetracycline marked minpp, Tet^r	This work
B. thetaiotomicron	GH120	GH59 containing pGH038 plasmid, Tet^r, Cc^r, expressing low levels of BtMinpp	This work
B. thetaiotomicron	GH115	GH59 containing pGH037 plasmid, Tet^r, Cc^r, expressing high levels of BtMinpp	This work

* Kana, kanamycin; Tet, tetracycline; Cm, chloramphenicol, Amp, ampicillin; Cc, clindamycin
