# Supplementary material for: A Bacterial Homolog of a Eukaryotic Inositol Phosphate Signaling Enzyme Mediates Cross-kingdom Dialog in the Mammalian Gut
Source: Cell Rep. 2014 Feb 13;6(4):646–56. doi: 10.1016/j.celrep.2014.01.021 (PMC3969271; doi:10.1016/j.celrep.2014.01.021)
Supplement: Document S2. Article plus Supplemental Information [file mmc2.pdf]

Cell Press
Open ACCESS
Cell Reports
Article

A Bacterial Homolog of a Eukaryotic Inositol Phosphate Signaling Enzyme Mediates Cross-kingdom Dialog in the Mammalian Gut

Régis Stentz,[1] Samantha Osborne,[1] Nikki Horn,[1] Arthur W.H. Li,[2] Isabelle Hautefort,[1] Roy Bongaerts,[1] Marine Rouyer,[1] Paul Bailey,[5] Stephen B. Shears,[6] Andrew M. Hemmings,[2,3] Charles A. Brearley,[2,*] and Simon R. Carding[1,4,*]
[1]Gut Health and Food Safety Programme, Institute of Food Research, Norwich NR4 7UA, UK
[2]School of Biological Sciences, University of East Anglia, Norwich NR4 7TJ, UK
[3]School of Chemistry, University of East Anglia, Norwich NR4 7TJ, UK
[4]Norwich Medical School, University of East Anglia, Norwich NR4 7TJ, UK
[5]Department of Computational and Systems Biology, John Innes Centre, Norwich NR4 7UH, UK
[6]Laboratory of Signal Transduction, National Institute of Environmental Health Sciences, Research Triangle Park, NC 27709, USA
*Correspondence: c.brearley@uea.ac.uk (C.A.B.), simon.carding@ifr.ac.uk (S.R.C.)
http://dx.doi.org/10.1016/j.celrep.2014.01.021
This is an open-access article distributed under the terms of the Creative Commons Attribution License, which permits unrestricted use, distribution, and reproduction in any medium, provided the original author and source are credited.

SUMMARY

Dietary $InsP_6$ can modulate eukaryotic cell proliferation and has complex nutritive consequences, but its metabolism in the mammalian gastrointestinal tract is poorly understood. Therefore, we performed phylogenetic analyses of the gastrointestinal microbiome in order to search for candidate $InsP_6$ phosphatases. We determined that prominent gut bacteria express homologs of the mammalian $InsP_6$ phosphatase (MINPP) and characterized the enzyme from *Bacteroides thetaiotaomicron* (BtMinpp). We show that BtMinpp has exceptionally high catalytic activity, which we rationalize on the basis of mutagenesis studies and by determining its crystal structure at 1.9 Å resolution. We demonstrate that BtMinpp is packaged inside outer membrane vesicles (OMVs) protecting the enzyme from degradation by gastrointestinal proteases. Moreover, we uncover an example of cross-kingdom cell-to-cell signaling, showing that the BtMinpp-OMVs interact with intestinal epithelial cells to promote intracellular Ca^{2+} signaling. Our characterization of BtMinpp offers several directions for understanding how the microbiome serves human gastrointestinal physiology.

INTRODUCTION

The adult human gastrointestinal (GI) tract accommodates a bacterial community that comprises trillions of cells. This microbiota has many essential roles in human health (Tremaroli and Bäckhed, 2012): it suppresses proliferation of pathogenic microbes and has important nutritional consequences, including vitamin synthesis and fermentation of complex dietary carbohydrates. Microbial metabolites also regulate the signaling activities of the host's intestinal epithelium, which, for example, aids the development and maintenance of local immune responses. Thus, considerable efforts are now being made to determine the precise nature of the dialog between gut bacteria and the human host. Much of the previous work in this area has focused on the roles of diffusible, small-molecule hormones and nutrients. We now describe a vehicle produced by a prevalent gut bacterium, *Bacteroides thetaiotaomicron* (Bt): a vesicle-enclosed homolog of a mammalian cell-signaling $InsP_6$ phosphatase, MINPP.

Enzymatic homeostasis of $InsP_6$ levels in the gut can have far-reaching consequences for human health. Considerable quantities of $InsP_6$ are ingested daily as it is the primary storage form of phosphorus in cereals and legumes (Kumar et al., 2010). $InsP_6$ is therefore a source of inositol and phosphate, two vital nutrients. However, $InsP_6$ is also considered to have antinutritive properties, given that it inhibits polysaccharide digestibility and chelates divalent cations, thereby limiting their bioavailability in the GI tract (Kumar et al., 2010). More recently, with the emergence of cell-signaling activities for $InsP_6$ and other members of the inositol phosphate family, dietary $InsP_6$ has received separate attention for its anticarcinogenic properties (Fox and Eberl, 2002; Vucenik and Shamsuddin, 2003), at least when present at high concentrations. Conversely, lower concentrations of $InsP_6$ may stimulate tumor cell proliferation (Windhorst et al., 2013). In any case, exogenous $InsP_6$ can enter cells and be metabolized (Windhorst et al., 2013), thereby potentially contributing to intracellular signaling processes in intestinal epithelial and immune cells. For example, $InsP_6$ is the precursor for inositol pyrophosphates, which have pleiotropic functions including the regulation of energy metabolism (Szijgyarto et al., 2011), insulin sensitivity (Chakraborty et al., 2010), and bactericidal activities of immune cells (Prasad et al., 2011).

Humans and other monogastric animals lack enzymes capable of participating in dietary $InsP_6$ homeostasis, relying instead on exogenous phosphatases provided by resident microbes (Haros et al., 2007; Steer et al., 2004). Surprisingly, the nature of the genes that encode these phosphatases

CrossMark

Cell PRESS
Open ACCESS

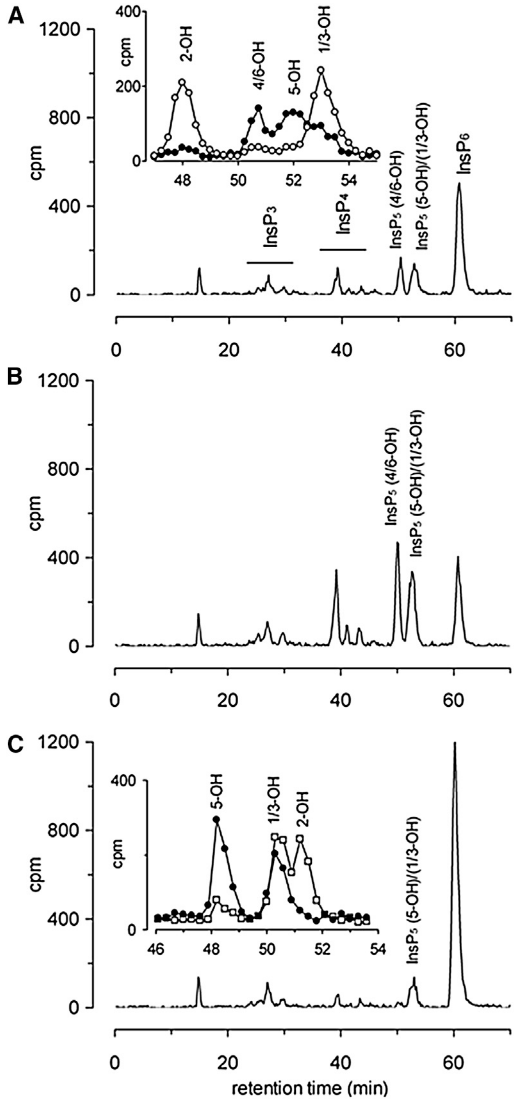

Figure 1. Mutation of Active Site Residues Alters the Specificity of Initial Attack on $InsP_6$ Substrate

(A–C) Products of reaction of native BtMinpp- (A), A31Y- (B), and R183D- (C) substituted enzyme with *myo*-inositol(1,[^{32}P]2,3,4,5,6)P_6 were resolved by Partisphere SAX HPLC. Mutated proteins were incubated at a concentration of 2.5 μg/ml and native protein at 0.25 μg/ml, with 1 mM $InsP_6$. The regions of the chromatogram in which $InsP_3$, $InsP_4$, and specific $InsP_5$ isomers elute are indicated in (A). For native enzyme (inset in A), reaction products were mixed with standards of *myo*-[2-^{3}H]inositol (1,3,4,5,6)P_5 ($InsP_5$ [2-OH]), D/L- *myo*-[2-^{3}H]inositol (1,2,4,5,6)P_5 ($InsP_5$ [1/3-OH]), and D/L- *myo*-[2-^{3}H]inositol (1,2,3,4,5)P_5 ($InsP_5$ [4/6-OH]). Fractions, 0.25 min, were collected and radioactivity was estimated by scintillation counting; ^{3}H, open circles; ^{32}P, filled circles. For R183D-substituted enzyme (inset in C), the reaction products were additionally mixed with standards of *myo*-[^{14}C]$InsP_5$ [2-OH] and D/L- *myo*-[^{14}C]$InsP_5$ [1/3-OH] and resolved on a Adsorbosphere SAX HPLC column. This column separates $InsP_5$ [2-OH] from $InsP_5$ [1/3-OH]. Fractions, 0.25 min, were collected and radioactivity estimated by scintillation counting: ^{14}C, open squares; ^{32}P, filled circles.

is unknown for virtually the entire microbiome of the human GI tract, although they are generally assumed to be "phytases" (Haefner et al., 2005; Steer et al., 2004; Tamayo-Ramos et al., 2012). Such enzymes occur in a number of environmental niches in which they scavenge inorganic phosphate from $InsP_6$, but they have no signaling function. Moreover, if phytases were to be secreted into the GI tract, it is unclear how they might access dietary $InsP_6$ while avoiding digestive proteases. In the current study, our phylogenetic, biochemical, and structural characterization of BtMinpp characterizes an unexpected repertoire of widely distributed and highly active class of $InsP_6$ phosphatases in the GI tract.

RESULTS

B. thetaiotaomicron Express a Homolog of a Mammalian Inositol Phosphate Signaling Phosphatase

The Bacteroidetes are a dominant constituent of the mammalian GI tract microbiota. The genome of *B. thetaiotaomicron* (Xu et al., 2003) contains a gene, *BT_4744*, which has a primary annotation (Hidden Markov Models-based annotation) as encoding a putative multiple inositol-polyphosphate phosphatase (MINPP, EC 3.1.3.62). This is a notable observation because bacteria have not previously been suggested to utilize the inositol phosphate signaling cascade (Michell, 2008). Indeed, MINPPs have only previously been considered to function inside animal cells, mainly by regulating levels of $InsP_5$ and $InsP_6$ (Chi et al., 2000; Romano et al., 1998). Strikingly, BT_4744 shares no significant sequence similarity with the bacterial or fungal phytases described in the literature (see Table S1). We shall refer to the *BT_4744*-encoded enzyme as BtMinpp, and below we further justify this characterization.

High-performance liquid chromatography (HPLC) analysis of the catalytic activity of a His-tagged recombinant form of BtMinpp confirmed that this protein has $InsP_6$ phosphatase activity (Figure 1A). The V_{max} value for $InsP_6$ (178 μmol/mg/min; Table S2) is the highest yet reported for any member of the MINPP family, being several orders of magnitude greater than that for the human (0.006 μmol/mg/min) and avian (0.7 μmol/mg/min) representatives (Ali et al., 1995; Cho et al., 2006). We incubated BtMinpp with Ins(1,[^{32}P]2,3,4,5,6)P_6 substrate and identified the $InsP_5$ products from their HPLC elution positions relative to *myo*-[2-^{3}H]$InsP_5$ standards (Figure 1A, inset). Although Ins(1,3,4,5,6)P_5 ($InsP_5$ [2-OH]) cannot be detected using Ins(1,[^{32}P]2,3,4,5,6)P_6 as substrate, we can be confident that $InsP_5$ [2-OH] is not a major product because of the retention of radiolabel in progressively less-phosphorylated products, $InsP_4$, $InsP_3$, and $InsP_2$ (Figure S1C). In any case, the use of Ins(1,[^{32}P]2,3,4,5,6)P_6 as substrate revealed that multiple $InsP_5$ products were formed by BtMinpp, including D-and/or L-Ins(1,2,3,4,5)P_5 ($InsP_5$ [4/6-OH]), Ins(1,2,3,4,6)P_5 ($InsP_5$ [5-OH]), and D-and/or L-Ins(1,2,4,5,6)P_5 ($InsP_5$ [1/3-OH]) (Figure S2). Such a lack of specificity toward the site of initial attack on $InsP_6$ is a defining characteristic of MINPPs of both plants (Dionisio et al., 2007) and animals (Figure S1A; Ali et al., 1995; Craxton et al., 1997). This catalytic flexibility contrasts with the more precise positional specificity of phytases. In fact, the distinct specificities of different types of phytases are sufficient

Cell PRESS
Open ACCESS

Table 1. X-Ray Data Collection and Structure Refinement Statistics

	SAD Data	Phosphate[a]	$InsS_6$[a]
Data Collection			
Wavelength (Å)	0.9799	0.9778	0.9778
Space group	P 2_1	P 2_1	P 2_1
Cell parameters			
a, b, c (Å)	53.1, 121.0, 76.0	52.7, 120.6, 76.1	52.2, 117.3, 75.5
β (°)	107.8	107.9	107.46
Resolution limits (Å) (high-resolution bin)[b]	50.00–2.50 (2.64–2.50)	62.07–1.93 (1.98–1.93)	61.44–2.42 (2.48–2.42)
R_{sym}[c]	0.098 (0.265)	0.048 (0.473)	0.069 (0.529)
<(I)/sd(I) >	16.4 (7.3)	12.6 (2.1)	10.5 (2.9)
Completeness (%)	100.0 (100.0)	97.1 (97.0)	99.2 (99.2)
Multiplicity	7.4 (7.5)	2.3 (2.4)	3.3 (3.2)
Anomalous completeness (%)	99.9 (100.0)	—	—
Anomalous multiplicity	3.8 (3.8)	—	—
Overall temperature factor ($Å^2$)		26.0	48.7
Refinement			
Protein monomers per asymmetric unit		2	2
Total nonhydrogen atoms		7194	6747
Water molecules		451	127
R_{cryst}[d] (%)		16.6 (23.5)	15.6 (19.7)
R_{free}[e] (%)		21.3 (28.2)	21.7 (27.0)
Ramachandran analysis (%)			
Most favored		97.58	97.37
Outliers		0.25	0.25
Rmsds			
Bonds, Å		0.007	0.008
Angles, °		0.976	1.17
Planes, Å		0.005	0.006
Mean atomic b value ($Å^2$)		24.2	39.8

[a]Phosphate and IHS refer to the complexes of BtMinpp with inorganic phosphate and *myo*-inositol hexakis sulfate, respectively.
[b]Figures in brackets refer to the high resolution data bin as indicated.
[c]$R_{sym} = \Sigma|I_i - \langle I \rangle|/ \Sigma I_i$ where $\langle I \rangle$ is the average of symmetry equivalent reflections and the summation extends over all observations for all unique reflections.
[d]$R_{cryst} = \Sigma||F_o|-|F_c||/\Sigma|F_o|$ where F_o and F_c are the measured and calculated structure factors, respectively
[e]For R_{free} the summations extends over a randomly selected subset (5%) of reflections excluded from all stages of refinement

to serve as a classification system: there are 3-phytases (E.C. 3.1.3.8) (see also Figure S1B), 6-phytases (E.C. 3.1.3.26), and 5-phytases (E.C. 3.1.3.72).

Although mammalian MINPPs exhibit positional promiscuity toward $InsP_6$ (Craxton et al., 1997), these enzymes are highly specific toward certain other inositol phosphate substrates. For example, they primarily remove the 3-phosphate from Ins(1,3,4,5,6)P_5 and Ins(1,3,4,5)P_4 (Craxton et al., 1997) and display a considerably higher V_{max} toward Ins(1,3,4,5)P_4 substrate than to $InsP_6$ (Nogimori et al., 1991). The *Bacteroides* enzyme shares with mammalian MINPPs increased catalytic activity toward Ins(1,3,4,5)P_4 substrate (Figure S2A), yielding a V_{max} value 20- to 36-fold higher than that obtained with $InsP_6$ (Table S2). In contrast, incubation of BtMinpp with Ins(1,3,4,5)P_4 (Figure S2A) yielded two $InsP_3$ peaks that coeluted with Ins(1,4,5)P_3 and Ins(1,3,4)P_3 standards. The pH optima of 2.5, 4.0, and 7.5 (Figure S3) overlaps with the pH profile of the human GI tract (Fallingborg, 1999), consistent with the successful adaptation of BtMinpp to this environment.

The Crystal Structure of BtMinpp in Complex with a Substrate Analog

There is no published description of the structures of any members of the MINPP family. The crystal structure (diffraction data were collected on beamlines I02 and I24 at the Diamond Light Source, UK) of BtMinpp expressed in *Escherichia coli* as an N-terminal 6 × His fusion protein was solved at pH 5.0 by SAD using phases from a selenomethionyl derivative. Refinement against native data at 1.93 Å resolution gave a final structural model with an R_{cryst} of 16.6% (R_{free} 21.3%) and excellent geometry. A single inorganic phosphate group is bound at the active sites of both molecular copies of BtMinpp in the crystallographic asymmetric unit, presumably scavenged from the phosphate buffer used during purification of the enzyme (Table 1; Figure S4). A second structure was solved by difference Fourier analysis at 2.42 Å resolution in which the inorganic phosphate ions are displaced by the nonhydrolysable $InsP_6$ analog, *myo*-inositol hexakissulfate ($InsS_6$), again bound in the active sites of both molecules (R_{cryst} 15.6%; R_{free} 21.7%) (Figure 2; Table 1). Comparison of the structures of analog- and phosphate-bound forms revealed an rmsd of 0.20 Å for C_α atoms suggesting limited conformational changes occur between the two states.

BtMinpp folds into two domains, an α/β domain and an α domain. Despite their low amino acid sequence homology, and functional diversity, all available crystal structures of members of branch 2 of the histidine phosphatase superfamily possess this general domain arrangement with a structurally conserved α/β domain (the "core" domain) and a more variable α-domain (the "cap" domain) (Rigden, 2008). The cleft between the two domains always contains the active site, which is highly specialized in each of the distinct branch two subfamilies, allowing them to perform different functions. The active site in BtMinpp is lined with predominantly basic amino acids, thereby rationalizing how the enzyme can bind unusually highly phosphorylated substrates such as $InsP_6$ (Figure 3A). $InsS_6$ is bound in the active site with its 3-sulfate group occupying the position taken by inorganic phosphate in the enzyme-phosphate complex and presumably that of the hydrolyzed phosphate group in substrates. This conclusion is consistent with the sulfur atom of this group lying 3.1 Å from the imidazole Nε2 atom of the side chain of H59. By analogy with other members of the superfamily, we predict that H59 of BtMinpp acts as a nucleophile during catalysis to generate a phosphohistidine intermediate. In phytases, the aspartate residue from a proximal HD sequence motif takes the role of proton

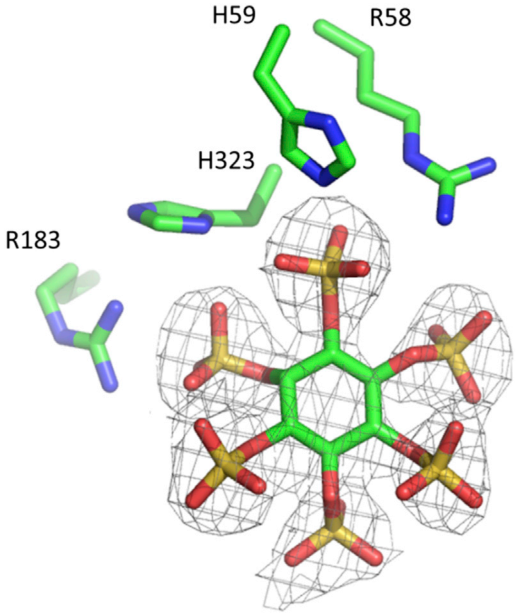

Figure 2. Simulated Annealing Omit Map Revealing the Location of $InsS_6$ Bound to the Active Site of BtMinpp

A region of the simulated annealing omit electron density map (gray lines) (Brünger and Rice, 1997) calculated with data to a resolution of 2.42 Å and contoured at 1.8 σ. Selected active site residues are shown in stick representation and labeled. The location of the inhibitor in the final refined structure of the complex is shown superimposed on the omit map electron density.

donor for the leaving group (Kostrewa et al., 1997). However, a distinguishing feature of mammalian and plant MINPPs is the substitution of this motif with an HAE tripeptide in which the glutamic acid residue (E325 in BtMinpp) is the candidate proton donor. Indeed, in our crystal complex with $InsS_6$ one of the E325 side-chain carboxyl oxygen atoms is only 3.3 Å from the ether oxygen bridging the 3-sulfate to the inositol ring, the analog to the phosphate-leaving group in $InsP_6$.

The structure of the $InsS_6$-protein crystal complex rationalizes the ability of BtMinpp to remove the 3-phosphate from its natural substrate, $InsP_6$. However, BtMinpp also removes alternative phosphates from $InsP_6$ (Figure 1A), indicating that the enzyme's active site can bind substrate in alternate orientations. To analyze which features of the catalytic core of BtMinpp permit this positional promiscuity, we compared its structure with that of *A. niger* PhyA phytase (Oakley, 2010), a member of a different subfamily of clade 2 histidine acid phosphatases, that hydrolyzes $InsP_6$ by specific removal of its 3-phosphate (as does the closely related *A. ficuum* PhyA; Figure S2B). A comparison of this PhyA with BtMinpp yielded an rmsd of 2.5 Å, albeit with low sequence identity (16% over 360 structurally equivalent residues; Table S3). The overall topologies of these two enzymes are superficially similar, which is why we have labeled those secondary structural elements in BtMinpp that correspond to those found in PhyA (Figures 3A and S5).

Accordingly, we performed an overlay of the $InsS_6$-bound structures of BtMinpp and *A. niger* phytase based on the coordinates of the backbone atoms of the active site fingerprint sequence (RHGXRXP; residues 58–64 in BtMinpp) found in the A-a loop and those of the short region containing the presumed proton donor, E325 (HAE; residues 323–325) from the N-terminal region of helix I. The rmsd was 0.30 Å for these 30 atoms. The conformations of $InsS_6$ were very similar (rmsd 0.50 Å for the six inositol ring carbon atoms) in these overlayed structures. Nevertheless, this superposition (Figure 3B) revealed some significant differences in the nature of the ligand-binding residues in the two proteins. Binding pocket S2 shows the most extensive differences. Here, R183, conserved in MINPP enzymes and forming an ion pair with the 2-sulfate of the ligand, replaces the Asp in PhyA. Additionally, the HAE tripeptide of BtMinpp replaces the characteristic HD sequence motif in PhyA. Both substitutions substantially alter the volume and character of S2. The substitution of the phytase proton donor (Asp) with Ala324 in BtMinpp increases volume and decreases polarity of both S2 and the adjacent S4 pockets. Finally, a tyrosine residue that contributes to pockets S4 and S5 is replaced by Ala31in BtMinpp. These substitutions at positions 31 and 324 make the S4 and S5 pockets of BtMinpp significantly larger than those in PhyA. The significance of the larger BtMinpp ligand-binding site is that it may permit alternate substrate binding modes. Molecular docking experiments further support the concept of a reduced discrimination against alternative $InsP_6$-binding modes in BtMinpp compared to PhyA (Figure 3C; Table S4): PhyA showed an exclusive preference for a limited set of highly similar $InsP_6$ binding modes all presenting the D-3 phosphate in binding pocket S3. Termed the obverse binding mode, this places the D-2 axial phosphate in the S2 binding pocket. BtMinpp was more permissive, allowing binding of not only the D-3 phosphate in pocket S3 but also the D-5 and D-6 phosphates. These additional binding modes present equatorial phosphates of $InsP_6$ in the S2 pocket and are distributed between obverse and reverse presentations (the latter having the inositol ring flipped over to present its opposite face to the enzyme). Given that the A31Y and R183D substitutions in phyA relative to BtMinpp were identified by our structural studies as making particularly significant contributions to the volume and polarity of the active site, we tested that hypothesis with a mutagenic approach.

Impact of Mutating the Active Site of BtMinpp on the Specificity of Initial Attack on $InsP_6$ Substrate

When incubated with $InsP_6$, the A31Y mutant of BtMinpp yielded an HPLC profile of $InsP_5$ products (Figure 1B) very similar to that observed for wild-type enzyme (Figure 1A). Thus, the positional specificity toward $InsP_6$ was not affected by this mutation. Nevertheless, it should be noted that the HPLC profile of the wild-type enzyme was obtained with one-tenth the protein concentration of that of the A31Y mutant (we estimated that the A31Y mutant was approximately 2.5-fold less active). Elevating the wild-type enzyme concentration 10-fold resulted in complete dephosphorylation of $InsP_5$ and $InsP_6$ (Figure S2B). Remarkably, the R183D substitution abolished the production of $InsP_5$ [4/6-OH], but retained a peak with the retention time of $InsP_5$ [5-OH]/ $InsP_5$ [1/3-OH] (Figure 1C). Using an Adsorbosphere SAX column to resolve $InsP_5$ [5-OH] from $InsP_5$ [1/3-OH] and $InsP_5$ [2-OH], we confirmed that the R183D mutant had lost the ability to attack the 4/6-position (compare Figures 1A and 1C),

Cell PRESS
Open ACCESS

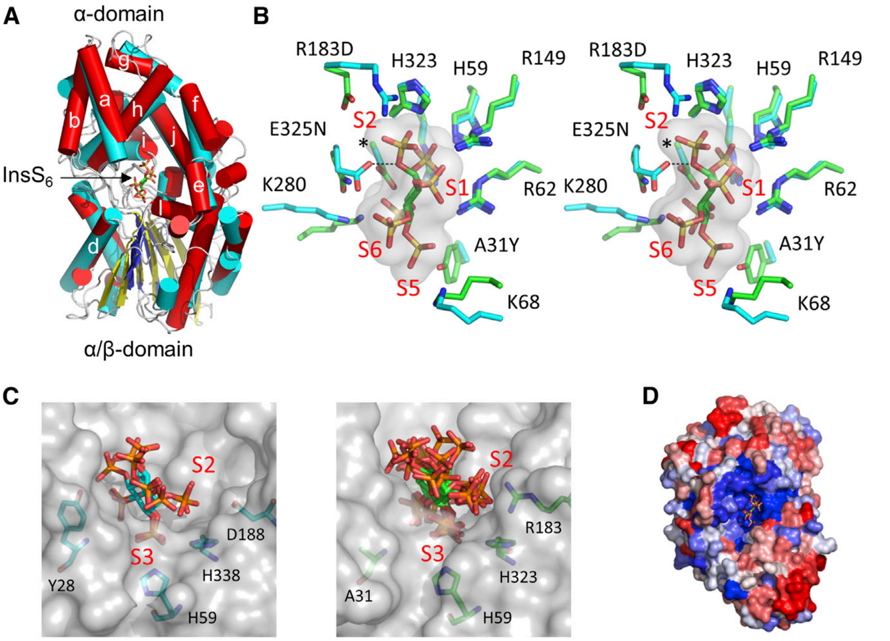

Figure 3. X-Ray Crystal Structure of the BtMinpp

(A) Cylinder and ribbon diagram showing a superposition of the crystal structures of BtMinpp (α helices in red, β sheet in yellow) and the *Aspergillus niger* phytase PhyA (Oakley, 2010) (α helices in cyan, β sheet in blue) both taken from their complexes with the $InsP_6$ analog, $InsS_6$. The core $\alpha+\beta$ and capping α-domains are indicated. The naming of α helices in the capping domain of BtMinpp (indicated by a lowercase letter) follows that suggested for PhyA (Oakley, 2010). The atoms of the bound substrate analog are shown as sticks.

(B) A stereoview of the superposition of the side chains of $InsS_6$-binding residues (shown as sticks) of BtMinpp (carbon atoms colored cyan) and *Aspergillus niger* PhyA (carbon atoms colored green). The residues selected for display form the primary sites of interaction with $InsS_6$ in both complexes. The van der Waals surface of $InsS_6$ is shown in gray. Residue labels follow the numbering for the BtMinpp structure. The leading character identifies the residue in BtMinpp, whereas the trailing identifies the corresponding variant residue found in PhyA. An asterisk (*) indicates the position of the A324D substitution. Selected binding pockets (S1, S2, S5, and S6) are indicated, numbered according to corresponding sulfate group on the bound ligand. Pocket S4 is obscured in this orientation by the 6-sulfate group of the ligand. The interaction distance between a carboxyl group oxygen of residue E325 and the bridging ether oxygen of the S3 sulfate group of $InsS_6$ is 3.3 Å and is indicated by a dashed line.

(C) Predicted binding modes within 1 kcal mol^{-1} of the minimum binding energy resulting from molecular docking experiments for $InsP_6$ with PhyA (left, three modes) and BtMinpp (right, seven modes). Selected active site residues are shown as sticks and labeled as are binding pockets S2 and S3. The enzyme molecular surfaces are shown in gray.

(D) View of a ConSurf (Landau et al., 2005) color-coded surface representation of BtMinpp. The normalized conservation scores calculated by ConSurf are a relative measure of evolutionary conservation at each residue position based on the alignment of 23 MINPP sequences. The highest scores (8 and 9 on the ConSurf scale) represent the most conserved residue positions and are shown colored blue. The residues with the lowest scores (i.e., the most variable) are colored red. The bound $InsS_6$ ligand is shown in stick format.

but retained the ability to attack the 5- and 1/3-positions (Figure 1C, inset). Thus, our mutagenic data confirm that the positional flexibility of BtMinpp toward $InsP_6$ reflects a larger and less discriminating active site compared to phyA. The R183D mutant was also about 10-fold less active than wild-type enzyme (compare Figures 1A and 1C), suggesting that increased catalytic activity may be an evolutionary advantage of BtMinpp's more flexible active site.

Given that human MINPP1 and BtMinpp share a common ability to attack $InsP_6$ substrate at different positions and remove a 3-phosphate from Ins(1,3,4,5)P_4 (see above), we considered whether our crystallographic analysis of BtMinpp offers an explanation of the substrate specificity of mammalian MINPPs. Analysis of the conservation of residues that are predicted to contribute atoms to the molecular surface of 23 representative MINPP enzymes from different kingdoms of life reveals highest sequence identity in a region of the active site centered on H59 (Figure 3D). There is 52% sequence identity between human MINPP1 and BtMinpp for those active site residues that have an atom within 8 Å of the $InsS_6$ bound to the bacterial enzyme. Interestingly, residue A31, forming part of binding pocket S5 in BtMinpp, is commonly replaced with a lysine in other MINPP enzymes (Figure S5). Introduction of a lysine residue may allow additional polar contacts with highly negatively charged substrates. Its size may also serve to make binding pocket S5 smaller. This substitution may therefore be of relevance in explaining the differential activity of *Bacteroides* and eukaryotic enzymes toward lower inositol phosphate substrates by enforcing more positional specificity on the mammalian enzyme.

The MINPP Protein Family Includes Microbial Members

Having defined Minpp as a bacterial product, we next performed a phylogenetic analysis to determine how widespread is the distribution of *minpp* homologs in microbial organisms. A BLASTP search of BtMinpp encoded protein sequence against the nonredundant protein database identified 326 sequences with significant alignments (e value $\leq 1 \times 10^{-4}$ and a minimal number of 60 identical amino acids over the entire sequence length). These included proteins of bacterial, animal, and plant origin. Whereas most bacterial phyla were represented, no representative of the Firmicutes, a major phylum of the human GI tract microbiota, was included. Nevertheless, a significant number of the other dominant GI-resident organisms (*Bacteroides*, *Bifidobacterium*, *Prevotella*, and *Alistipes)* encode proteins belonging to the MINPP family. In contrast, a search for microbial genes predicted to encode microbial phytase enzymes among 341 genomes of the human GI tract (Human Microbiome Project at http://commonfund.nih.gov/hmp/index) revealed that microbial phytases are scarce in this environment and are only present in the genome of subdominant bacterial species (Table S5).

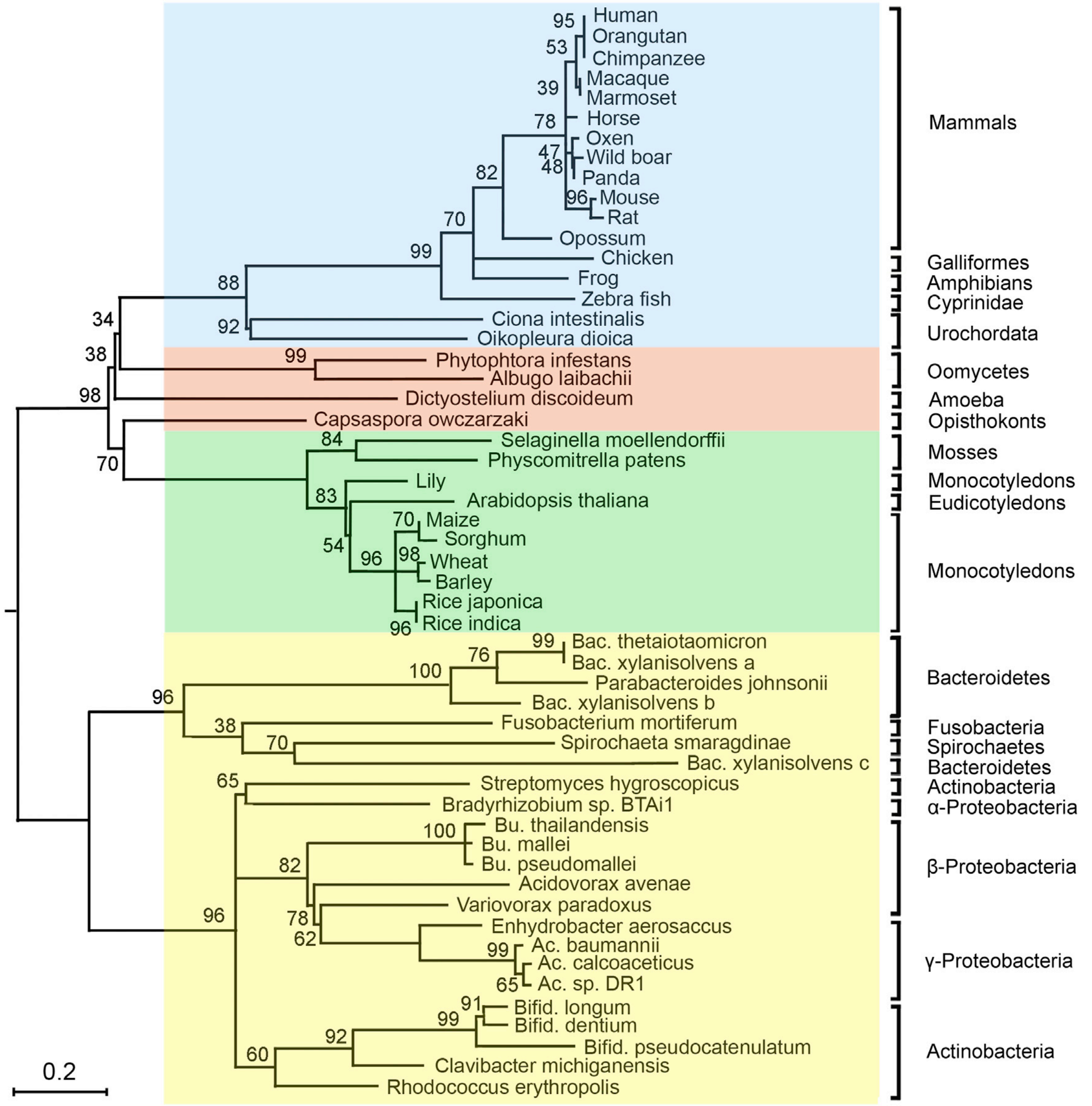

Figure 4. The MINPP Family Phylogenetic Tree

A phylogenetic tree derived from the alignment of 54 MINPP protein representatives from different kingdoms of life was constructed using the Maximum Likelihood method. The different kingdoms have been assigned the following background colors: animals, blue; plants, green; protists, salmon-pink; bacteria, yellow. Ac, *Acinetobacter*; Bac, *Bacteroides*; Bu, *Burkholderia*; Bifid, *Bifidobacterium*. The tree is drawn to scale, with branch lengths measured in the number of substitutions per site.

This analysis leads us to conclude that bacterial Minpps are the major representative of $InsP_6$-degrading enzymes within the GI tract of humans and are encoded by dominant bacterial species that are niche-adapted human symbionts.

A phylogenetic tree (Figure 4) was constructed from the alignment of 54 representatives (Table S6) of different kingdoms of life. In general, MINPP-related sequences are found within the bacteria, plant, and animal kingdoms with the sequences in plants and animals being evolutionarily conserved within their respective clade. However, in the bacterial group, the Bacteroidetes phylum does not group with the Actinobacteria phylum as previously reported (Ciccarelli et al., 2006). The different Minpps from the bacterial species displayed in the tree all appear equally related to their eukaryotic homologs (Figure 4) with the eukaryotic and bacterial proteins each forming monophyletic groups, suggesting an ancient origin for the MINPP family of proteins.

Among the 2,536 completed bacterial genomes listed in EMBL-EBI (http://www.ebi.ac.uk/genomes/bacteria.html), only 55 contained one or more copies of a gene homologous to BtMinpp, accounting for 2.2% of the completed genomes. In contrast, greater than 50% of animal and plant completed genomes contained at least one copy of a gene predicted to encode a MINPP representative. Our phylogenetic analyses strengthen the conclusion from our biochemical and structural

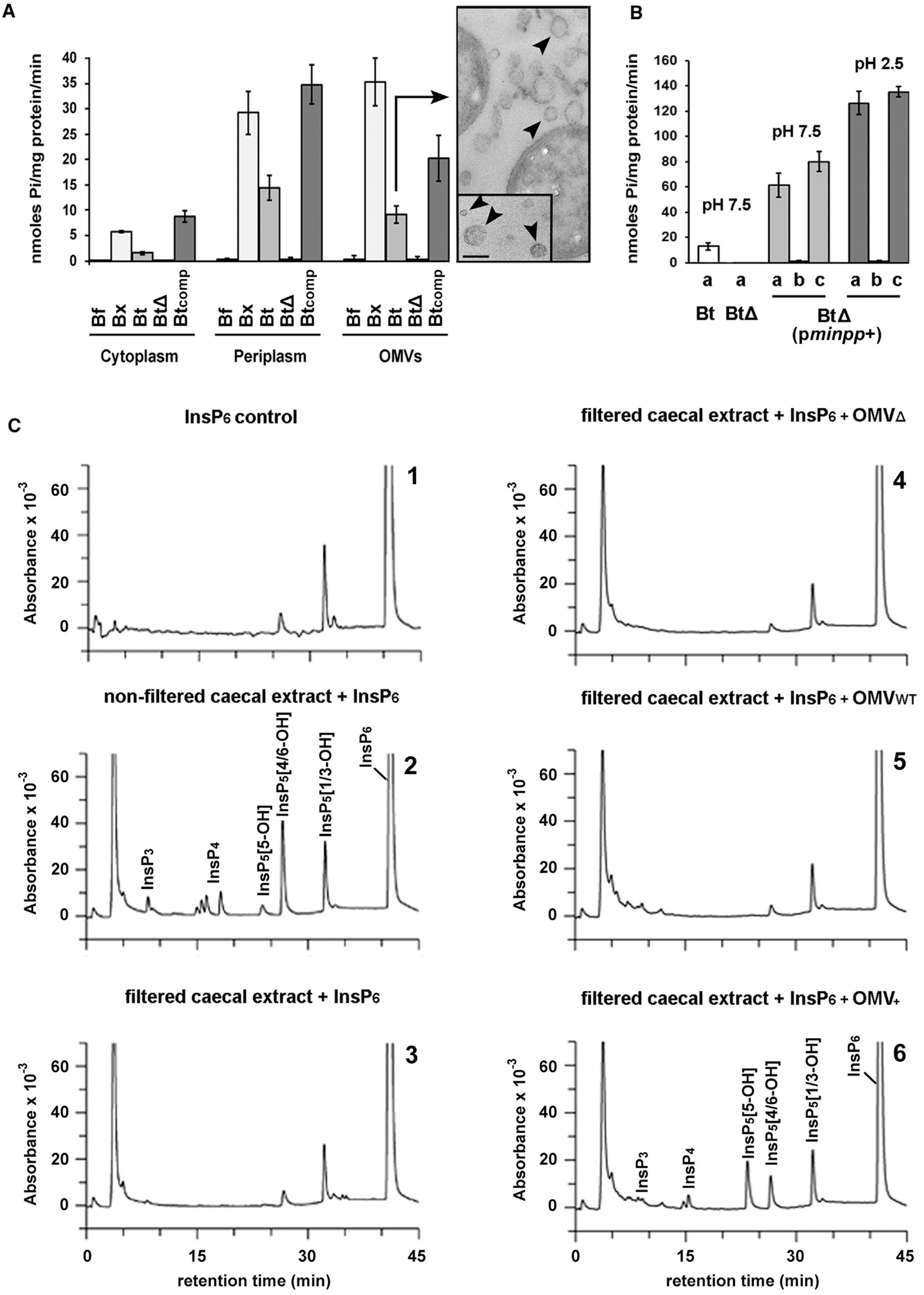

(legend on next page)

studies (see above) that the bacterial Minpp-related protein sequences constitute a different family, distinct from phytases, but nevertheless a subset of clade 2 of the histidine phosphatase superfamily (HAP; IPR000560) (Rigden, 2008).

$InsP_6$ Metabolism by BtMinpp Secreted from *B. thetaiotaomicron* in Outer Membrane Vesicles

The key question of how BtMinpp in the gut survives a hostile protease-containing environment yet accesses extracellular $InsP_6$ was addressed. BtMinpp is predicted to contain an N-terminal signal peptide (http://www.cbs.dtu.dk/services/SignalP/), so we hypothesized that the protein would be secreted into the periplasmic space. Indeed, the periplasmic fraction of *B. thetaiotaomicron* showed substantial Minpp activity (Figure 5A). This observation led us to investigate if BtMinpp might also be packaged into outer membrane vesicles (OMVs). Although OMV blebbing from the cell surface has been observed in different *Bacteroides* species, *B. fragilis* is the only example for which various enzymatic activities in OMVs have previously been assayed (Patrick et al., 1996). Transmission electron microscopy confirmed the presence of membrane blebs and the release of intact OMVs by *B. thetaiotaomicron* (Figure 5A). Substantial $InsP_6$ phosphatase activity was detected in OMV protein extracts (Figure 5A).

We prepared the protein fraction from the cytoplasm, periplasm and OMVs of a strain of *B. thetaiotaomicron* from which we had deleted the *minpp* gene. All of these fractions had negligible $InsP_6$ phosphatase activity (Figure 5A), indicating that BtMinpp accounts for the $InsP_6$ phosphatase activity observed in the corresponding fractions prepared from the wild-type strain (Figure 5A). Expression of *minpp* in *trans* in the mutant increased the activity to levels higher than in the wild-type parental strain in each of the subcellular fractions (Figure 5A). It is noteworthy that no $InsP_6$ hydrolysis activity was detected in another human gut *Bacteroides* species, *B. fragilis*, that does not contain a gene related to *minpp.* In contrast, high levels of $InsP_6$ phosphatase activity were detected in the periplasm and OMVs of *B. xylanisolvens* (Figure 5A), which contains three copies of the *minpp* gene.

We next investigated the capacity of intact vesicles containing BtMinpp to degrade $InsP_6$ in the external milieu. $InsP_6$ was hydrolyzed by OMVs produced and isolated from the wild-type strain, whereas no phosphate release from $InsP_6$ was detected by OMVs produced by the Minpp1-deficient strain (Figure 5B). We also engineered a *B. thetaiotaomicron* strain that overexpresses BtMinpp and recorded between 6 and 12 times more $InsP_6$ degradation for its isolated OMVs compared to those from wild-type *B. thetaiotaomicron* (Figure 5B). The conditioned media from which OMVs had been removed showed no enzyme activity; sonication of the OMVs was required for enzyme activity to be released (Figure 5B). These findings are consistent with OMVs being intact and that the enzyme is retained in the vesicles and is not released due to leakage.

The functional significance of this packaging of BtMinpp in OMVs was investigated in physiological experiments using the cecal contents of mice to which $InsP_6$ substrate was added and using HPLC to detect $InsP_6$ metabolites. Note that in these experiments the commercial $InsP_6$ sample was contaminated with small peaks of $InsP_5$ (Figure 5C1). Control cecal extracts contained $InsP_6$-phosphatase activity (Figure 5C2) that was no longer detected after filtration using a 100 kDa cutoff membrane (Figure 5C3), capable of removing large protein complexes or OMVs produced by resident bacteria. In further experiments, no significant $InsP_6$-phosphatase activity was observed in filtered cecal extracts supplemented with OMVs prepared from Minpp-deficient bacteria (Figure 5C4). The assay was also unable to detect $InsP_6$ phosphatase activity in OMVs produced by wild-type *B. thetaiotaomicron* (Figure 5C5), probably due to limitations in its sensitivity (see Figure 5B). However, when filtered cecal contents were supplemented with OMVs produced by *B. thetaiotaomicron* that overexpress Minpp, considerable $InsP_6$-phosphatase activity was detected (Figure 5C6). Collectively, these findings demonstrate that BtMinpp is retained inside the OMVs, which $InsP_6$ must enter in order to access the BtMinpp.

Minpp1-Loaded Vesicles Induce the Release of Intracellular Calcium in Colonic Epithelial Cells

It is well established that OMVs produced by bacterial pathogens such as *Helicobacter pylori*, *Legionella pneumophila*, or *E. coli* are capable of interacting with host cells via a membrane fusion event or via adhesin-receptor-mediated attachment to deliver virulence factors such as proteases and toxins (Ellis and Kuehn, 2010). We hypothesized therefore that either

Figure 5. Intact OMVs Produced by *B. thetaiotaomicron* Degrade Exogenous $InsP_6$

(A) Minpp activity was assessed in different fractions of *B. thetaiotaomicron* and other *Bacteroides* spp. using $InsP_6$ as a substrate and a molybdate/malachite green-based enzyme assay to measure released phosphate. The different fractions were extracted from *B. fragilis* (Bf), *B. xylanisolvens* (Bx), *B. thetaiotaomicron* (Bt), Bt *minpp*-deleted mutant strain GH59 (Bt_{Δ}), and the same strain containing the pGH38 plasmid (GH120) expressing BtMinpp (Bt_{comp}). The values shown represent the mean ± SEM values obtained from five to six independent experiments. The TEM images show Bt cells and associated OMVs (arrowed) with the inset showing images of isolated OMVs. Scale bar, 100 nm.

(B) (a) Degradation of $InsP_6$ by intact OMVs produced by *B. thetaiotaomicron* wild-type strain (Bt), the *minpp*-deleted mutant (Bt_{Δ}), and the *minpp*-deleted mutant containing the pGH037 plasmid overexpressing BtMinpp (Bt_{Δ} (p*minpp*+). OMVs were isolated, concentrated, and resuspended in a Tris buffer (pH 7.5) and for Bt_{Δ} (p*minpp*+) in a glycine-HCl buffer (pH 2.5) to which $InsP_6$ substrate was added and the suspension was incubated for 1 hr at 37°C. (b) BtMinpp activity was measured in the buffer fraction of Bt_{Δ} (p*minpp*+) OMV suspensions after incubation for 1 hr at 37°C and removal of OMVs. (c) Enzyme activity was measured in sonicated extracts of OMVs recovered from the mixtures described in (b). The values shown represent the mean ± SEM values obtained from at least five independent experiments.

(C) Cecal contents were obtained from C57BL/6 mice as described in the Experimental Procedures. Each HPLC chromatogram panel shown is representative of samples from four different mice. $InsP_6$ was added to all samples. (C1) $InsP_6$ control, showing contaminating $InsP_5$s of this commercial $InsP_6$ sample; (C2) nonfiltered cecal extract; (C3) cecal extracts filtered (100 kDa molecular weight cutoff membrane); (C4–6) cecal extracts filtered (100 kDa molecular weight cutoff membrane) and supplemented with OMVs from either a BtMinpp-deleted mutant (C4), the WT strain (C5), or a strain overexpressing Minpp (C6).

Cell PRESS
Open ACCESS

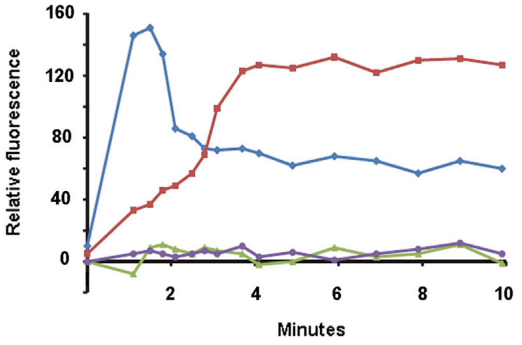

Figure 6. Minpp1-Loaded Vesicles Modify Colonic Epithelial Cell Signaling

Fluorescence intensity of Fluo-8 AM-loaded HT29 cells in response to treatment with ionomycin (blue), OMVs from a BtMinpp-deleted mutant (purple), OMVs containing the overexpressed BtMinpp (red), or PBS alone (green). The data shown are representative of three experiments, each with three replicates.

mechanism might be used by *B. thetaiotaomicron* OMVs to deliver BtMinpp into host intestinal epithelial cells, with the potential consequence that BtMinpp could interact with the inositol polyphosphate signaling pathways of host cells. Indeed, Yu et al. (2003) have shown that in mammalian cells expression of an engineered truncated cytosolic form of the endoplasmic reticulum-confined MINPP1 leads to a significant enhancement of Ins(1,4,5)P_3 concentration, triggering the release of calcium from intracellular stores via the $InsP_3$ receptor/Ca^{2+} channel. To test these ideas, BtMinpp-containing OMVs were added to HT29 human colonic epithelial cells in the absence of extracellular calcium. Immediately after addition of the OMVs, a gradual increase in intracellular calcium concentration was observed that plateaued within approximately 4 min (Figure 6). By contrast, no calcium response was observed when HT29 cells were incubated with OMVs that did not contain any BtMinpp. This result suggests that OMVs interact with epithelial cells leading to the release of BtMinpp and generation of $InsP_3$ products. Additionally, it is possible that attachment of OMVs to target cells may locally increase the concentration of an $InsP_6$ metabolite that stimulates Ca^{2+} mobilization. Nevertheless, irrespective of their mechanism of action, OMVs containing BtMinpp are able, in vitro, to trigger the release of calcium from intracellular stores to the cytosol of colonic epithelial cells.

DISCUSSION

The main impact of our study centers on the identification of a homolog of a eukaryotic inositolphosphate phosphatase, MINPP, in major species of human gut bacterial genomes. Detailed biomolecular and phylogenetic analyses of Minpp from *B. thetaiotaomicron* (BtMinpp) validated it as a member of the MINPP family with an exceptionally high catalytic activity that makes it exquisitely suited for facilitating $InsP_6$ homeostasis in the mammalian GI tract. Moreover, we rationalize the catalytic activity and evolutionary conservation of BtMinpp by mutagenic studies and by an atomic-level description of the structure of this enzyme at 1.9 Å resolution. We further demonstrate that BtMinpp is packaged inside OMVs, thereby protecting the phosphatase activity from degradation by gastrointestinal proteases, and also facilitating an example of cross-kingdom, long-range, cell-to-cell signaling; we show that the OMVs that are released by *B. thetaiotaomicron* are able to deliver BtMinpp to intestinal epithelial cells, promoting intracellular Ca^{2+} signaling.

The physiological significance of $InsP_6$ phosphatase activity in the human gut is likely complex and multifactorial. First, there is nutritional benefit to both the host and the bacterial community from the inorganic phosphate and the inositol moiety that are both released. Additionally, the hydrolysis of $InsP_6$ eliminates its antinutritive properties, such as divalent ion chelation and inhibition of polysaccharide digestibility. High concentrations of $InsP_6$ have been considered to have anticarcinogenic properties in the human colon (Fox and Eberl, 2002; Vucenik and Shamsuddin, 2003). However, a recent paper indicates that lower and more physiologically relevant levels of $InsP_6$ in the diet might promote tumor development (Windhorst et al., 2013). This last finding raises the possibility of further human health impacts of bacterial Minpp in the GI tract.

B. thetaiotaomicron is a dominant human GI symbiont. Moreover, our phylogenetic studies indicate that the majority of other bacteria known to harbor a *minpp* gene are also members of the human intestinal microbiota. These 149 representatives are from the *Bacteroides*, *Bifidobacterium*, *Prevotella*, and *Alistipes* communities. Indeed, our analysis of $InsP_6$ hydrolysis ex vivo from endogenous cecal contents indicates that the endogenous enzymatic activity is positionally promiscuous toward its substrates, and contained in OMVs. These are both properties of BtMinpp making it likely that bacterial Minpps are a widespread yet hitherto unappreciated source of highly active $InsP_6$ phosphatase activity in the GI tract.

Our characterization of the crystal structure of BtMinpp provides a description of members of the MINPP family at the atomic level. In particular, the structure of the protein in complex with $InsS_6$, a substrate analog, gave insight into the reaction mechanism. We propose that H59 acts as a nucleophile for cleavage of the 3-phosphate from $InsP_6$, with E325 in an HAE tripeptide being the proton donor for the leaving group. Our structural work also illuminates the positional promiscuity of the MINPP superfamily, which distinguishes it from the various classes of phytases in the HAP family that each exhibits a distinct preference for a particular phosphate group (e.g., 3-phytases, 4/6-phytases, and 5-phytases; Konietzny and Greiner, 2002; Kumar et al., 2010). Our overlay of the structure of the active sites of *A. niger* 3-phytase and BtMinpp revealed that the latter has a larger and less polar ligand-binding pocket that, according to our molecular models, accepts $InsP_6$ in several different orientations (Figure 3C). Furthermore, in a synthetic biology approach, we mutated R183 in BtMinpp to the corresponding Arg in the 3-phytase in *A. niger*. This resulted in BtMinpp losing the ability to remove the 4/6-phosphate from $InsP_6$ (Figure 1). That is, the R183D mutation caused the positional preference of the enzyme to become more phytase-like in nature.

Interestingly, A31 in BtMinpp is commonly replaced with Lys in other MINPPs (Figure S5), and so this residue may be of relevance in explaining the differential positional specificities of *Bacteroides* and eukaryotic enzymes toward lower inositol

phosphate substrates. For example, the mammalian MINPPs only removes the 3-phosphate from Ins(1,3,4,5)P_4 (Caffrey et al., 1999), whereas the bacterial enzyme is less selective. It is an intriguing possibility that a lack of positional selectivity may be associated with elevated catalytic activity of BtMinpp against $InsP_6$. Nevertheless, we found a 52% sequence identity between human MINPP1 and BtMinpp for those active site residues that have an atom within 8 Å of the $InsS_6$ that cocrystallized in the active site. Such a high degree of evolutionary conservation would suggest that bacterial and mammalian Minpps have originated from a common primordial ancestor. It is alternately possible that bacteria acquired Minpps from eukaryotes by horizontal gene transfer (HGT), although known cases of this phenomenon are rare (Keeling and Palmer, 2008). Our phylogenetic analyses were unable to distinguish between these two alternatives.

Our characterization of the properties of a vesicularized bacterial homolog of a mammalian signaling enzyme challenges established orthodoxies concerning our understanding of the mechanisms by which symbiotic bacteria in the mammalian GI tract interact with their host. First, it has been thought that OMVs primarily mediate pathogenic processes (Ellis and Kuehn, 2010). Our data reveal that commensal gut bacteria also utilize OMVs in a manner that is beneficial to the host, by contributing to $InsP_6$ homeostasis. The ability of BtMinpp-containing OMVs to stimulate intracellular Ca^{2+} release in human colonic epithelial cells suggests a further biological significance to bacterial Minpps, namely, a role in interkingdom communication pathways (Figure 6). Another example of this phenomenon is the OMV-mediated delivery of immunoregulatory capsular polysaccharide A from *B. fragilis* to host dendritic cells to effect disease protection (Shen et al., 2012). Nevertheless, the application of an enzyme to mediate dialog between gut bacteria and the human host is an addition to a field of research that has previously focused on the roles of diffusible, small-molecule hormones and nutrients. Further research into variations in the expression and secretion of Minpps by the different species of GI bacteria could increase our insight into the complex interrelationships between the microbiome and the human host.

EXPERIMENTAL PROCEDURES

Bacterial Strains and Growth Conditions

All *E. coli* and *Bacteroides* strains used in this study are listed in Table S7. The bacterial growth conditions are described in the Supplemental Information.

HPLC

Inositol phosphate products of assays using ^{32}P-labeled substrate were resolved by HPLC, details of which are provided in the Supplemental Information.

Crystal Structure Determination

A single crystal of selenomethionyl-derivitized BtMinpp was used to collect a SAD data set, which was processed using the CCP4 package to obtain a crystal structure as described in the Supplemental Information.

Phylogenetic Analysis

The evolutionary history of the MINPP protein was inferred using the Maximum Likelihood method in the MEGA5 software tool (Tamura et al., 2011). Amino acid sequences were aligned with PRANK and highly variable regions removed from the data set. The Whelan and Goldman (2001) model of evolution was used, and initial tree(s) for the heuristic search was obtained automatically as follows. When the number of common sites was <100 or less than one-fourth of the total number of sites, the maximum parsimony method was used; otherwise, BIONJ method with MCL distance matrix was used. To provide statistical support for each node on the tree, a consensus tree was generated from 1,000 bootstrap data sets.

Periplasmic and OMV Protein Extraction and BtMinpp Phosphatase Activity Measurement

The method of Osborn et al. (1972) was used to obtain periplasmic fractions of *B. thetaiotaomicron* and OMVs were harvested from bacterial cultures by ultracentrifugation and assayed for phosphatase activity using the PiColorLock Gold Phosphate Detection System as described in the Supplemental Information.

Intracellular Calcium Measurement

HT-29 cells were propagated in Sarstedt culture flasks (25 cm^2) in a 5% CO_2 humidified atmosphere at 37°C. Cells were fed with DMEM (Lonza) supplemented with 10% heat-inactivated FBS (Biosera) and 2 mM L-glutamine (Lonza). For the intracellular calcium measurement assay, cells were seeded in 24-well microplates (Corning CellBind Surface) at a density of 10^5 cells/well. After 16 hr, the culture medium was replaced with fresh medium containing 5 μM of the calcium indicator Fluo-8 AM (AAT Bioquest), 4 mM of Probenecid (Sigma-Aldrich), and 0.025% (w/v) Pluronic acid F-127 (Invitrogen) according to a method adapted from Abrahamse and Rechkemmer, 2001, and cells were incubated for 30 min at 37°C, 5% CO_2. The medium was removed, and calcium-free PBS was added before the cells were incubated at 37°C for another 30 min. The cell monolayer was washed twice with calcium-free PBS and PBS was added. Fluo-8 fluorescence was measured immediately after addition of Ionomycin (Sigma-Aldrich) 1 μg/ml, PBS, or purified OMVs (corresponding to 10 μg of soluble protein) with a FLUOstar Optima fluorescence plate reader (BMG Labtechnologies) fitted with custom excitation/emission filters (485/538 nm). OMVs were purified from 500 ml culture supernatants, washed with calcium-free PBS, and concentrated to a 2 ml suspension.

ACCESSION NUMBERS

The Protein Data Bank ID codes for the atomic coordinates and structure factors for the phosphate- and $InsS_6$-bound complexes of BtMinpp reported in this paper are 4FDT and 4FDU, respectively.

SUPPLEMENTAL INFORMATION

Supplemental Information includes Supplemental Experimental Procedures, five figures, and seven tables and can be found with this article online at http://dx.doi.org/10.1016/j.celrep.2014.01.021.

AUTHOR CONTRIBUTIONS

R.S. and S.R.C. conceived the study and R.S., S.R.C., C.A.B., S.B.S., and A.M.H. wrote the paper. R.S., S.R.C., C.A.B., A.M.H., P.B., and S.B.S. designed experiments and analyzed data. R.S., S.O., N.H., A.W.H.L., C.A.B., I.H., R.B., M.R., and S.B.S. performed the experiments. S.R.C. and S.B.S. obtained funding to support the study.

ACKNOWLEDGMENTS

We would like to thank Kathryn Cross for help with electron microscopy, Dr. Ida Porcelli for providing advice on periplasmic extraction, and Dr. Nathalie Juge for scientific discussions. This work was supported by institutional grants from Biotechnology and Biological Sciences Research Council (BBSRC; BB/J004529/1, S.R.C.), by a BBSRC postgraduate studentship (BB/F016816/1, S.O.), and by the Intramural Research Program of the National Institutes of Health and National Institute of Environmental Health Sciences (S.B.S.).

Cell PRESS Open ACCESS

Received: August 30, 2013
Revised: December 13, 2013
Accepted: January 15, 2014
Published: February 13, 2014

REFERENCES

Abrahamse, S.L., and Rechkemmer, G. (2001). Identification of an organic anion transport system in the human colon carcinoma cell line HT29 clone 19A. Pflugers Arch. *441*, 529–537.

Ali, N., Craxton, A., Sumner, M., and Shears, S.B. (1995). Effects of aluminium on the hepatic inositol polyphosphate phosphatase. Biochem. J. *305*, 557–561.

Brünger, A.T., and Rice, L.M. (1997). Crystallographic refinement by simulated annealing: methods and applications. Methods Enzymol. *277*, 243–269.

Caffrey, J.J., Hidaka, K., Matsuda, M., Hirata, M., and Shears, S.B. (1999). The human and rat forms of multiple inositol polyphosphate phosphatase: functional homology with a histidine acid phosphatase up-regulated during endochondral ossification. FEBS Lett. *442*, 99–104.

Chakraborty, A., Koldobskiy, M.A., Bello, N.T., Maxwell, M., Potter, J.J., Juluri, K.R., Maag, D., Kim, S., Huang, A.S., Dailey, M.J., et al. (2010). Inositol pyrophosphates inhibit Akt signaling, thereby regulating insulin sensitivity and weight gain. Cell *143*, 897–910.

Chi, H.B., Yang, X.N., Kingsley, P.D., O'Keefe, R.J., Puzas, J.E., Rosier, R.N., Shears, S.B., and Reynolds, P.R. (2000). Targeted deletion of Minpp1 provides new insight into the activity of multiple inositol polyphosphate phosphatase in vivo. Mol. Cell. Biol. *20*, 6496–6507.

Cho, J., Choi, K., Darden, T., Reynolds, P.R., Petitte, J.N., and Shears, S.B. (2006). Avian multiple inositol polyphosphate phosphatase is an active phytase that can be engineered to help ameliorate the planet's "phosphate crisis". J. Biotechnol. *126*, 248–259.

Ciccarelli, F.D., Doerks, T., von Mering, C., Creevey, C.J., Snel, B., and Bork, P. (2006). Toward automatic reconstruction of a highly resolved tree of life. Science *311*, 1283–1287.

Craxton, A., Caffrey, J.J., Burkhart, W., Safrany, S.T., and Shears, S.B. (1997). Molecular cloning and expression of a rat hepatic multiple inositol polyphosphate phosphatase. Biochem. J. *328*, 75–81.

Dionisio, G., Holm, P.B., and Brinch-Pedersen, H. (2007). Wheat (Triticum aestivum L.) and barley (Hordeum vulgare L.) multiple inositol polyphosphate phosphatases (MINPPs) are phytases expressed during grain filling and germination. Plant Biotechnol. J. *5*, 325–338.

Ellis, T.N., and Kuehn, M.J. (2010). Virulence and immunomodulatory roles of bacterial outer membrane vesicles. Microbiol. Mol. Biol. Rev. *74*, 81–94.

Fallingborg, J. (1999). Intraluminal pH of the human gastrointestinal tract. Dan. Med. Bull. *46*, 183–196.

Fox, C.H., and Eberl, M. (2002). Phytic acid (IP6), novel broad spectrum antineoplastic agent: a systematic review. Complement. Ther. Med. *10*, 229–234.

Haefner, S., Knietsch, A., Scholten, E., Braun, J., Lohscheidt, M., and Zelder, O. (2005). Biotechnological production and applications of phytases. Appl. Microbiol. Biotechnol. *68*, 588–597.

Haros, M., Bielecka, M., Honke, J., and Sanz, Y. (2007). Myo-inositol hexakisphosphate degradation by Bifidobacterium infantis ATCC 15697. Int. J. Food Microbiol. *117*, 76–84.

Keeling, P.J., and Palmer, J.D. (2008). Horizontal gene transfer in eukaryotic evolution. Nat. Rev. Genet. *9*, 605–618.

Konietzny, U., and Greiner, R. (2002). Molecular and catalytic properties of phytate-degrading enzymes (phytases). Int. J. Food Sci. *37*, 791–812.

Kostrewa, D., Grüninger-Leitch, F., D'Arcy, A., Broger, C., Mitchell, D., and van Loon, A.P. (1997). Crystal structure of phytase from Aspergillus ficuum at 2.5 A resolution. Nat. Struct. Biol. *4*, 185–190.

Kumar, V., Sinha, A.K., Makkar, H.P.S., and Becker, K. (2010). Dietary roles of phytate and phytase in human nutrition: A review. Food Chem. *120*, 945–959.

Landau, M., Mayrose, I., Rosenberg, Y., Glaser, F., Martz, E., Pupko, T., and Ben-Tal, N. (2005). ConSurf 2005: the projection of evolutionary conservation scores of residues on protein structures. Nucleic Acids Res. *33* (Web Server issue), W299–W302.

Michell, R.H. (2008). Inositol derivatives: evolution and functions. Nat. Rev. Mol. Cell Biol. *9*, 151–161.

Nogimori, K., Hughes, P.J., Glennon, M.C., Hodgson, M.E., Putney, J.W., Jr., and Shears, S.B. (1991). Purification of an inositol (1,3,4,5)-tetrakisphosphate 3-phosphatase activity from rat liver and the evaluation of its substrate specificity. J. Biol. Chem. *266*, 16499–16506.

Oakley, A.J. (2010). The structure of *Aspergillus niger* phytase PhyA in complex with a phytate mimetic. Biochem. Biophys. Res. Commun. *397*, 745–749.

Osborn, M.J., Gander, J.E., Parisi, E., and Carson, J. (1972). Mechanism of assembly of the outer membrane of Salmonella typhimurium. Isolation and characterization of cytoplasmic and outer membrane. J. Biol. Chem. *247*, 3962–3972.

Patrick, S., McKenna, J.P., O'Hagan, S., and Dermott, E. (1996). A comparison of the haemagglutinating and enzymic activities of Bacteroides fragilis whole cells and outer membrane vesicles. Microb. Pathog. *20*, 191–202.

Prasad, A., Jia, Y.H., Chakraborty, A., Li, Y.T., Jain, S.K., Zhong, J., Roy, S.G., Loison, F., Mondal, S., Sakai, J., et al. (2011). Inositol hexakisphosphate kinase 1 regulates neutrophil function in innate immunity by inhibiting phosphatidylinositol-(3,4,5)-trisphosphate signaling. Nat. Immunol. *12*, 752–760.

Rigden, D.J. (2008). The histidine phosphatase superfamily: structure and function. Biochem. J. *409*, 333–348.

Romano, P.R., Wang, J., O'Keefe, R.J., Puzas, J.E., Rosier, R.N., and Reynolds, P.R. (1998). HiPER1, a phosphatase of the endoplasmic reticulum with a role in chondrocyte maturation. J. Cell Sci. *111*, 803–813.

Shen, Y., Giardino Torchia, M.L., Lawson, G.W., Karp, C.L., Ashwell, J.D., and Mazmanian, S.K. (2012). Outer membrane vesicles of a human commensal mediate immune regulation and disease protection. Cell Host Microbe *12*, 509–520.

Steer, T.E., Gee, J.N., Johnson, I.T., and Gibson, G.R. (2004). Biodiversity of human faecal bacteria isolated from phytic acid enriched chemostat fermenters. Curr. Issues Intest. Microbiol. *5*, 23–39.

Szijgyarto, Z., Garedew, A., Azevedo, C., and Saiardi, A. (2011). Influence of inositol pyrophosphates on cellular energy dynamics. Science *334*, 802–805.

Tamayo-Ramos, J.A., Sanz-Penella, J.M., Yebra, M.J., Monedero, V., and Haros, M. (2012). Novel phytases from Bifidobacterium pseudocatenulatum ATCC 27919 and Bifidobacterium longum subsp. infantis ATCC 15697. Appl. Environ. Microbiol. *78*, 5013–5015.

Tamura, K., Peterson, D., Peterson, N., Stecher, G., Nei, M., and Kumar, S. (2011). MEGA5: molecular evolutionary genetics analysis using maximum likelihood, evolutionary distance, and maximum parsimony methods. Mol. Biol. Evol. *28*, 2731–2739.

Tremaroli, V., and Bäckhed, F. (2012). Functional interactions between the gut microbiota and host metabolism. Nature *489*, 242–249.

Vucenik, I., and Shamsuddin, A.M. (2003). Cancer inhibition by inositol hexaphosphate (IP6) and inositol: from laboratory to clinic. J. Nutr. *133* (*Suppl 1*), 3778S–3784S.

Whelan, S., and Goldman, N. (2001). A general empirical model of protein evolution derived from multiple protein families using a maximum-likelihood approach. Mol. Biol. Evol. *18*, 691–699.

Windhorst, S., Lin, H.Y., Blechner, C., Fanick, W., Brandt, L., Brehm, M.A., and Mayr, G.W. (2013). Tumour cells can employ extracellular Ins(1,2,3,4,5,6)P(6) and multiple inositol-polyphosphate phosphatase 1 (MINPP1) dephosphorylation to improve their proliferation. Biochem. J. *450*, 115–125.

Xu, J., Bjursell, M.K., Himrod, J., Deng, S., Carmichael, L.K., Chiang, H.C., Hooper, L.V., and Gordon, J.I. (2003). A genomic view of the human-Bacteroides thetaiotaomicron symbiosis. Science *299*, 2074–2076.

Yu, J., Leibiger, B., Yang, S.N., Caffery, J.J., Shears, S.B., Leibiger, I.B., Barker, C.J., and Berggren, P.O. (2003). Cytosolic multiple inositol polyphosphate phosphatase in the regulation of cytoplasmic free Ca2+ concentration. J. Biol. Chem. *278*, 46210–46218.

Supplemental Information

Supplemental Experimental Procedures

Bacterial growth conditions

Purification of recombinant BtMinpp

BtMinpp enzymatic properties

HPLC

Inositol phosphates

$InsP_6$ assays

$InsP_4$ assay

Crystal structure determination

Molecular docking calculations

Site directed mutagenesis

Comparison of the structure of BtMinpp with other Branch 2 Histidine Phosphatases

Construction of *minpp* deletion mutant

Complementation of the *minpp* deletion mutant

Periplasmic protein extraction

Glucose-6-phosphate dehydrogenase assay

Outer membrane vesicles protein extraction

$InsP_6$ phosphatase activity measurement

Measurement of Minpp activity in the extracellular medium

Measurement of the catalytic activity of BtMinpp in resuspended OMVs

References

Supplemental Figure Legends

Figure S1, Related to Figure 1. The products of BtMinpp activity against $InsP_6$ are distinct from the products of attack of fungal phytase.

Figure S2, Related to Figure 1. The products of hydrolysis of Ins(1,3,4,5)P_4 and $InsP_6$

Figure S3, Related to Figure 1. Relative $InsP_6$ phosphatase activity (%) of recombinant BtMinpp at different pH values.

Figure S4, Related to Figure 2 Active site electron density for the complex of BtMinpp with inorganic phosphate.

Figure S5, Related to Figure 2. An alignment of the sequences of BtMinpp and selected MINPPs.

Supplemental Tables

Table S1, Related to Figure 1. Alignment of BT_4744 protein sequence with known MINPPs and phytases

Table S2, Related to Figure 1. Molecular and enzymatic properties of BtMinpp

Table S3, Related to Figure 2. Statistics from the comparison of BtMinpp with the structures of selected branch 2 histidine phosphatases

Table S4, Related to Figure 2.. Predicted low energy binding modes resulting from *in silico* docking of $InsP_6$ to enzyme structures

Table S5, Related to Figure 4. Genes annotated as phytases in the microbiome of the human gastrointestinal tract

Table S6, Related to Figure 4. Source organisms of MINPP proteins

Table S7, Related to Experimental Procedures. *E. coli* and *Bacteroides* strains used in the study

Materials and methods

Bacterial growth conditions

E. coli was grown at 37°C in Luria-Bertani medium (Sambrook & Russel, 2001) supplemented with 100 µg/mL ampicillin and/or 34 µg/mL chloramphenicol, 50 µg/mL kanamycin or 100 µg/mL spectinomycin. *Bacteroides* species were grown under anaerobic conditions at 37°C in BHI medium (Oxoid) supplemented with 0.001% haemin (BHIH). Antibiotic-resistance markers in *Bacteroides thetaiotaomicron* were selected using tetracycline 1 µg/mL, erythromycin 5 µg/mL or gentamicin 200 µg/mL. *E. coli* electrocompetent cells were prepared and transformed by the method of Sambrook and Russel (Sambrook & Russel, 2001).

Purification of recombinant and selenomethionine-labeled BtMinpp

The 405 amino acids product of the BtMinpp C-terminal region excluding the 20 amino acid N-terminal predicted signal peptide was purified using the His-Tag technique. A PCR fragment was generated using the primer pair MINPPam (CATGCATATGCAAACTAAGATACAGAAGTA) and MINPPav (CATGGGATCCCTATTCATTGAAAACTGA) and the resulting fragment was cloned into the *Nde*I/*Bam*HI restriction sites of the pET-15b expression vector (Novagen), which carries an N-terminal His-Tag sequence. The resulting plasmid pGH07 was used to transform BL21-CodonPlus(DE3)-RIL *E. coli* cells (Stratagene) and B834 (DE3) (Novagen) transformed with the pACYC-based tRNA-encoding plasmid RIL (Stratagene). The Ni-NTA Fast Start kit (Qiagen) was used to purify the protein according to the instruction of the manufacturer. Cells were grown at 30°C during 16 hours with a concentration of 0.5 mM IPTG. The imidazole used to elute the protein was removed using a PD-10 desalting column (Amersham Biosciences) and the buffer exchanged with 50 mM Tris-HCl, 300 mM NaCl pH7.2. The protein concentration was determined by direct UV measurement at 280 nm and by using the Bradford method. For the production of selenomethionine-labelled His-tagged BtMinpp, the plasmid pGH07 was transformed into the *E. coli* protease-deficient B834(DE3) strain (Novagen) containing the pACYC-based tRNA-encoding plasmid RIL (Stratagene)

using double selection with ampicillin (100 µg/mL) and chloramphenicol (34 µg/mL). The resulting strain was grown overnight in LB medium and 20mL of culture was used to inoculate 1 liter of Medium Base and Nutrient Mix (Athena Enzyme Systems) supplemented with 40 µg/mL L-selenomethionine solution (Athena Enzyme Systems). The cells were grown to an OD of 0.5 before induction with 0.5 mM IPTG. Cells were harvested after 16 h induction and recombinant selenomethionine-labelled proteins were extracted and purified using the Ni-NTA Fast Start kit (Qiagen) as described above.

BtMinpp enzymatic properties

Standard assay. $InsP_6$ phosphatase activity was measured using the PiColorLock Gold Phosphate Detection System (Innova Biosciences) based on the change in absorbance of the dye malachite green in the presence of phosphomolybdate complexes. 10 µM $InsP_6$ (Merck) was mixed with purified BtMinpp or protein extracts pre-treated with PiBind™ resin (Innova Biosciences) that removes contaminating Pi from buffers.

pH optimum and pH stability. The assay was performed using a variety of buffers: pH 1.0–3.5, 0.2 M glycine–HCl; pH 4.0–6.0, 0.05 M sodium acetate; pH 6.5–9.5, 0.2 M Tris–HCl. To determine the optimal pH, 2 ng of BtMinpp was incubated for 5 minutes at 37°C at different pH in the presence of 100µM $InsP_6$ and Pi release was measured under the standard $InsP_6$ dephosphorylation assay conditions. pH stability was determined after dilution and incubation of 10 ng of enzyme in different buffers and storage at 4°C for 24 hours.

Effect of temperature on enzyme activity. The temperature profile of the purified enzyme was determined in the temperature range from 10 C° to 80 C° using the standard assay. To check thermal stability, the purified enzyme was incubated at different temperatures for 10 minutes, cooled to 4 C°, and assayed using the standard $InsP_6$ dephosphorylation assay.

Effect of pepsin on enzyme activity. Enzyme inactivation by pepsin was determined by incubating 20 mU of BtMinpp in 0.2 M glycine–HCl pH 2.5, containing 3000 U pepsin for 30 min at 37 C°. After incubation, $InsP_6$ phosphatase activity was determined using the standard $InsP_6$ dephosphorylation assay.

HPLC

Inositol phosphate products of assays using ^{32}P-labelled substrate were resolved by HPLC on a 23.5 cm x 4.6 mm i.d.WVS Partisphere SAX column (Whatman, UK, Ltd) fitted with a SAX guard cartridge (Whatman). The column was eluted at 1 mL/min with a gradient formed by mixing solvents from buffer reservoirs containing: water (A) and 1.25M-$(NH_4)_2HPO_4$, adjusted to pH 3.8 with H_3PO_4 (B). Solvents were mixed according to the following gradient: time (min), %B; 0,0; 5,0; 65,100; 75,100. Additional separations were performed on a 25 cm x 4.6 mm Alltech Adsorbosphere SAX column (Grace Davison Discovery Sciences, Deerfield, IL, USA) fitted with a Alltech guard cartridge. The column was eluted at 1 mL/min with a gradient formed by mixing solvents from buffer reservoirs containing: water (A) and 1.25M-$(NH_4)_2HPO_4$, adjusted to pH 3.8 with H_3PO_4 (B). Solvents were mixed according to the following gradient: time (min), %B; 0,0; 5,0; 65,25; 67,0. Radioactivity was estimated by Cerenkov counting with a Canberra Packard (Pangbourne, Berks, UK) A500 series Radiomatic Flo Detector fitted with a 0.5 mL flow cell. The integration interval for counting was 12 s. Data was exported from the FloOne for Windows software (Canberra Packard) as an ASCII file and redrawn in Delta Graph v.4.0 (DeltaPoint, Monterey, CA). In some experiments fractions (0.2 or 0.3 min) were collected and radioactivity estimated by Cerenkov counting in a scintillation counter. Subsequently, 1mL water and 4mL Optima Flo-AP (Canberra Packard) scintillation fluid were added and the fractions were counted for ^{3}H or ^{14}C by scintillation counting in either a Wallac (Turku, Finland) 1409 or LabLogic (Sheffield, UK) 300SL scintillation counter.

Inositol phosphates

Myo-inositol(1,[^{32}P]2,3,4,5,6)P_6 was prepared using recombinant AtIPK1 according to Nagy et al. (Nagy et al, 2009). *Myo*-[2-^{3}H]inositol-l and *myo*-[^{14}C]inositol-labelled $InsP_5$ standards were prepared by acid treatment of the respective Ins(1,3,4,5,6)P_5 according to Brearley and Hanke (Brearley & Hanke, 1996).

Assays with Ins(1,[^{32}P]2,3,4,5,6)P_6 substrate

BtMinpp was assayed at 37 °C by the addition of 10 µL of 0.44 mg/mL protein in 20 mM glycine-HCl, pH 2.5, to 1 mL of 10 mM sodium salt of $InsP_6$ (Sigma) containing approximately 74 kBq Ins(1,[^{32}P]2,3,4,5,6)P_6. Aliquots of the assay were removed at intervals and subjected to chromatography. The data shown in Figure S3C was obtained after 60 min. Human MINPP1 was assayed at a protein concentration of 0.1 mg/mL in 25 mM Tris, 150 mM NaCl, 2mM $MgCl_2$, pH 7.2, with approximately 3.7 kBq of Ins(1,[^{32}P]2, 3,4,5,6)P_6. Reaction products were analyzed after 2 hours at 37 °C (Figure S3A). *Aspergillus ficuum* phytase (Sigma) was assayed at 37 °C by the addition of 10 µL of 0.44 mg/mL protein in 20 mM glycine-HCl, pH 2.5, to 1 mL of 10 mM sodium salt of $InsP_6$ (Sigma) containing approximately 7.4 kBq Ins(1,[^{32}P]2,3,4,5,6)P_6. The data shown in Figure S3B were obtained after 60 min. Assays of BtMinpp mutants were performed, beside native protein, at 37 °C by the addition of 1 µL of 1 mg/mL protein to 0.4 mL of 10 mM sodium salt of $InsP_6$ (Sigma) in 50 mM glycine-HCl, pH 3.5, containing approximately 7.4 kBq Ins(1,[^{32}P]2,3,4,5,6)P_6. The data shown in Figure 1 were obtained after 2 min. Reactions were stopped by boiling for 2 min, before addition of nucleotide standards (to monitor chromatography) and radiolabelled standards (to confirm identity of $InsP_5$ peaks).

Assay of BtMinpp with Ins(1,3,4,5)P_4 substrate

BtMinpp (2.5 ng/mL) was incubated for 30 min at 37 ^{0}C with 10 µM [^{3}H]Ins(1,3,4,5)P_4 in assay buffer containing 25 mM HEPES, 50 mM KCl. Assays were quenched and neutralized and analyzed by HPLC as described previously (Craxton et al, 1997).

Crystal structure determination

Recombinant BtMinpp was purified prior to crystallization by gel filtration using a Superdex 75 16/60 column and concentrated to 1 mg/ml. Crystallization proceeded by vapour diffusion using a 1:1 mixture of BtMinpp and a precipitant containing 0.2 M ammonium acetate pH 5.0, 18 % (w/v) PEG 3350. This crystal form, of space group $P2_1$, has a solvent content of 48 % and has 2 molecules of Minpp in the asymmetric unit. A native dataset was collected on beamline I-02 of the Diamond Light

Source (DLS) (Didcot, UK) from a single crystal at 100 K cryoprotected with 25 % (v/v) ethylene glycol. Crystals of selenomethionyl-derivitized BtMinpp were obtained by the same procedure except that the precipitant employed for crystallization contained 0.2 M imidazole-malate pH 6.0, 15 % (w/v) PEG 4000. A single crystal was used to collect a SAD (single-wavelength anomalous dispersion) dataset on beamline I-24 of the DLS at the Se K edge (λ = 0.9799 Å) to a resolution of 2.50 Å. Datasets were processed using MOSFLM (Leslie, 1999) and SCALA (Evans, 2006) as part of the CCP4 package (Collaborative Computational Project, 1994). The structure of SeMet Minpp was determined by SAD phasing using SHELX (Sheldrick, 2008) and chain-traced with BUCCANEER (Cowtan, 2006). This interim structural model was transferred to the cell of the native protein and refined against the native protein structure factors using PHENIX (Adams et al, 2010), alternating with manual rebuilding in COOT (Emsley & Cowtan, 2004). Refinement converged to give a final model for native Minpp displaying R-work and R-free values of 16.6 % and 21.3 %, respectively (Figure S4). When analyzed for stereochemical quality using MOLPROBITY (Chen et al, 2010) the final structure has only 2 residues (Ala 220 in both molecular copies in the AU) in the disallowed region of the Ramachadran plot. Following refinement this structure was found to contain a single inorganic phosphate ion bound at the active site. To produce crystals of substrate analogue-bound enzyme, phosphate-bound crystals were soaked in mother liquor solution containing 1 mM *myo*-inositol hexakis (hydrogen sulfate) hexapotassium salt ($InsS_6$) and 25 % (v/v) ethylene glycol. Collection of X-ray diffraction data followed on beamline I-24 of the DLS giving a dataset to a resolution of 2.42 Å. The structure of the $InsS_6$-bound complex was solved and refined to give R-work and R-free values of 15.6 % and 21.7 %, respectively. When analyzed for stereochemical quality using MOLPROBITY (Chen et al, 2010), the $InsS_6$-bound structure again has only residue Ala 220 in both molecular copies in the disallowed region of the Ramachandran plot. Full data collection, phasing and refinement statistics for all structures reported herein are presented in Table S3III.

Molecular docking calculations

Molecular docking experiments using torsionally-flexible phytate as ligand and the crystal structures of BtMinpp and *A.niger* PhyA (Kostrewa et al, 1997) as receptors were carried out using AutoDock

Vina (Trott & Olson, 2010). A D-2 axial model for $InsP_6$ (D-2 axial and five equatorial phosphates) was used as representative of the predominant conformation at the acidic pH used in our hydrolysis assays (Isbrandt & Oertel, 1980). Atomic coordinates for the ligand obtained from the Hic-Up database (Kleywegt et al, 2003). The structures of ligand and receptor were formatted with AutoDockTools 1.5.4 (Morris et al, 2009). The chemical bonds involving the phosphate groups of $InsP_6$ were defined as rotatable giving a total of 12 degrees of torsional freedom. Fixed (i.e. inflexible) enzyme models were used. A search space of $16 \times 16 \times 16$ Å^3 was used, centred on and encompassing each enzyme active site. Docking poses were visualized with PyMOL (The PyMOL Molecular Graphics System, Version 1.3, Schrödinger, LLC). Productive binding modes were assigned as those poses found with (i) binding energies within 1 kcalmol^{-1} of the global energy minimum and (ii) a His59 imidazole Nε2 atom to phosphate phosphorous distance of 4 Å or less. These were classified according to the phosphate groups bound in the S3 and S2 specificity subsites (Table SIV).

Site-directed mutagenesis

The A31Y and R183D BtMinpp protein variants were generated using the PCR-based Phusion Site-Directed Mutagenesis kit (Thermo Scientific) according to the manufacturer's instructions. The primer pairs A31Y (GAAGTATGCAGGGACGTACATGCCCTATCCTAATAG), A31Y_antisense (TGTATCTTAGTTTGCATATGGCTGCCG) and R183D (CAATATAATCATATCCTTGATTTTTTTGATCTGAATAAATC), R183D_antisense (TTTTCCTTCACTTCGCTGTAC) were used to obtain the A31Y and R183D amino acid substitutions, respectively, using pGH07 as a template. The resulting plasmids were used to transform Rosetta2(DE3)pLysS *E. coli* cells and protein expression and purification were carried out as described above.

Comparison of the structure of BtMinpp with other Branch 2 Histidine Phosphatases

The refined crystal structure of BtMinpp was compared to a representative subset of the available branch 2 histidine phosphates of known structure (Rigden, 2008). Dali-Lite (Holm & Park, 2000) was used to perform the structural alignments. Residue identities are calculated as the percentage of

residues found to be structurally-equivalent and chemically identical in the alignment. RMSDs are based on structurally-equivalent alpha-carbon atom positions. The protein structures with the following PDB codes were included: 1. Phytase PhyA from *A.fumigatus* (AfPhyA), PDB entry 1SK8, Uniprot sequence entry O000092 (Liu et al, 2004); 2. Phytase PhyA from *A.niger* (AnPhyA), 3K4Q, P34752 (Oakley, 2010); 3. Phytase PhyB from *A.niger* (AnPhyB), 1QFX, P34755 (Kostrewa et al, 1999); 4. Phytase from *Debaryomyces castellii* (DcPhyt), 2GFI, A2TBB4 (Ragon et al, 2009); 5. Phytase AppA from *E.coli* (EcAppA), 1DKL, P07102 (Lim et al, 2000); 6. Glucose-1-phosphatase from *E.coli* (EcG1Pase), 1NT4, P19926 (Lee et al, 2003); 7. Histidine acid phosphatase from *Francisella tularensis* (FtHAP), 3IT1, Q2A612 (Singh et al, 2009); 8. Phytase from *Hafnia Alvei* (HfPhyA), 4ARO, H9TUK5 (Ariza et al, 2013); 9. Human prostatic acid phosphatase (HsPAP), 1CVI, P15309 (Jakob et al, 2000); 10. Rat prostatic acid phosphatase (RnPAP), 1RPA, P20646 (Lindqvist et al, 1993).

Construction of *minpp* deletion mutant

A 755 bp chromosomal DNA fragment downstream from *minpp*, including the last 196 nucleotides within the C-terminal region, was amplified by PCR and cloned into the *Sph*I/*Sal*I sites of the pBluescript SK- vector into *E. coli* GC10 cells (Sigma). Subsequently, a 2.2-kb PCR-generated *Hind*III/*Pst*I fragment containing the tetracycline resistance gene *tetQ* from the *Bacteroides* plasmid pBT-2 (Tancula et al, 1992) was cloned into the *Hind*III/*Pst*I sites of the resulting plasmid, upstream from the 5'-truncated *minpp* gene. A *Sal*I/*Sac*I fragment containing the tetQ and 'minpp genes was cloned into the *E. coli-Bacteroides* shuttle suicide vector pFD516 (Smith et al, 1995). Finally, a 783 bp chromosomal DNA fragment upstream from *minpp*, including the first 244 nucleotides within the N-terminal region, was amplified by PCR and cloned into the *Sph*I/*Sal*I sites of *the* resulting pFD516-based plasmid. The new plasmid pGH051 containing the Δ*minpp*::*tetQ* construct, was mobilized from *E. coli* GC10 into *B. thetaiotaomicron* by triparental filter mating protocols (Shoemaker et al, 1986) using *E. coli* HB101(pRK2013) as the helper strain. Transconjugants were selected on BHI-hemin agar containing gentamicin and tetracycline. Determination of sensitivity to either tetracycline or erythromycin was carried out to identify recombinants that were tetracycline resistant and

erythromycin sensitive. PCR analysis and sequencing were used to confirm the double-crossover genetic allele exchange. A transconjugant, GH59, containing the Δ*minpp*::*tetQ* construct inserted into the *B. thetaiotaomicron* chromosome was selected for further studies.

Complementation of the *minpp* deletion mutant and overexpression

To complement the *B. thetaiotaomicron minpp* deletion mutant GH59, the *Bacteroides* expression vector pFI2716 (Wegmann et al, 2013) was employed. The primer pair *Bsp*HI_minPP1_5' (ATTCATGAAAAGATTATTATTTGTT) and minPP1_*Bam*HI_3' (TAGGATCCTATTCATTGAAAACTGA) was used to amplify a 1279 bp region encoding *minpp* from *B. thetaiotaomicron* VPI-5482 genomic DNA. The *minpp* fragment was digested with *Bsp*HI and *Bam*HI before cloning into the *Nco*I/*Bam*HI sites of the *Bacteroides* expression vector pFI2716, creating pGH035. Conversion of tetracycline resistance to clindamycin-erythromycin resistance: the primer pair ccr_amont2 (CATGCATATGAGCTCCATGCTATAGCTACC) and ccr_aval2 (CATGGGATCCGCCAGCCGTTATGCGGCAGC) was used to amplify a 1252 bp region encoding *ermF* from the plasmid pFD516 (Smith et al, 1995). The *ermF* fragment was digested with *Nde*I and cloned into *Nde*I digested (blunted) and *Nsi*I digested pGH035, to replace the existing 1803 bp *tetQ* portion of pGH035, creating pGH038. For overexpression purposes the *Bsp*HI and *Bam*HI digested minpp fragment described above was cloned into similar sites of the medium-level expression vector pGH020 (Wegmann et al, 2013) creating pGH036. This was followed by the conversion from tetracycline resistance to erythromycin resistance as described above, creating pGH037. The transformation of the *minpp* deletion mutant GH59 with pGH037 resulted in the creation GH115.

Periplasmic protein extraction

Bacteroides species were grown in 20 mL of BHI supplemented with 0.001% hemin for 16 hours. The cells were centrifuged at 3500 g for 10 minutes and the periplasmic fraction was prepared according to the method described by Osborn *et al*. (Osborn et al, 1972). Briefly, the cell pellet was resuspended in 4 ml of fractionation buffer (Tris 30 mM, sucrose 20%, EDTA 1mM, pH8) and incubated for 10 minutes at 20°C. The cell suspension was centrifuged for 10 minutes at 3000 g and the cell pellet was

resuspended in 0.8 ml of ice-cold 5 mM $MgSO_4$ and the suspension was left on ice for 10 minutes. The osmotic shock fluid was harvested by centrifugation for 10 minutes at 3000 g, 4°C. To verify that no cross-contamination of cytoplasmic proteins occurred, glucose-6-phosphate dehydrogenase activity was assessed (described in *S1 Materials and Methods*) in the different periplasmic fractions and no activity was detected.

Glucose-6-phosphate dehydrogenase assay

100 µl of periplasmic or cytoplasmic extract was added to the following solution, pre-incubated at 30°C: 2.5 mL of 50 mM glycylglycine buffer pH 7.4, 0.2 mL of 150 mM $MgCl_{2,}$; 0.1 mL of 20 mM NADP and 0.1 mL of 60 mM glucose-6-phosphate (Noltmann et al, 1961). The increase in A_{340}/min for 4 to 5 minutes was recorded (UVIKON XS spectrophotometer, NorthStar Scientific) and the A_{340}/minute from the initial linear portion of the curve was calculated.

Outer membrane vesicles protein extraction

Cultures of bacteria were centrifuged at 5000 g for 15 minutes at 4ºC and the supernatant filtered through a 0.22 µm PES membrane (Sartorius) to remove debris and cells. Supernatants were concentrated by molecular weight (100 kDa MWCO, Sartorius) and the retentate ultracentrifuged (150,000 g for 2 h at 4 °C in a Ti70 rotor (Beckman Instruments). The supernatant was carefully aspirated from the tubes and the vesicle pellet was resuspended with 25 mM Tris buffer (pH 7.4). The pellet was washed with the same Tris buffer and centrifuged at 16000 g for 30 min. The pellet was resuspended in Tris buffer and OMV sterility examined by checking for growth of any contaminating bacterial cells on BHIH agar. The OMVs were disrupted by sonication. The OMV protein content was determined using the Total Protein Micro protein assay reagent kit (Sigma).

$InsP_6$ phosphatase activity measurement

Contaminating inorganic phosphate was removed from *Bacteroides* cytoplasmic, periplasmic and OMV fractions using PiBind™ resin (Innova Biosciences) prior to $InsP_6$ phosphatase activity measurements. The phosphatase activity was measured after diluting 10 µl of phosphate-depleted

extracts with Tris buffer (25mM, pH 7.5) in the presence of 10 μM of $InsP_6$ (Merck) and the mixture was incubated at 37ºC for 1 hour. $InsP_6$ dephosphorylation was measured using a molybdate/malachite green-based activity assay for the quantification of phosphate release (PiColorLock Gold Phosphate Detection System, Innova Biociences) according to the manufacturer's instructions.

Measurement of Minpp activity in the extracellular medium

20 mL of *Bacteroides* 16-hour cultures in BHI-hemin were centrifuged at 3500 g for 10 minutes and the supernatants were filtered through a 0.22 μm PES membrane (Sartorius) to remove debris and cells. The supernatants were then filtered through a 1000 kDa MWCO (Sartorius) filter to remove OMVs and the filtrate was further filtrated through a 10 kDa MWCO filter (Sartorius) to concentrate the 20 mL solution containing Minpp (49 kDa) down to 200 µl (100x). $InsP_6$ phosphatase activity was measured following depletion of inorganic phosphate with PiBind™ resin (Innova Biosciences) in 10 µl of the concentrated solution using the malachite green activity assay.

Measurement of the catalytic activity of BtMinpp in resuspended OMVs

Bacterial cells from 500 mL cultures were centrifuged at 5000 g for 20 minutes at 4ºC and the supernatant filtered through a 0.22 µm PES membrane (Sartorius) to remove debris and cells. Supernatants were concentrated by molecular weight (100 kDa MWCO) using a Vivaflow 50 ultrafiltration device (Sartorius) at 4ºC. To rinse the vesicles, the 5 mL of residual retentate was diluted 100 times in Tris buffer (25mM, pH 7.5) and the resulting 500 mL concentrated to 5 mL. The vesicles were further concentrated to 2 mL with a Vivaspin 20 centrifugal concentrator (Sartorius) and filtered through a 0.22 µm PES membrane (Sartorius). Vesicles concentrations were adjusted according to the protein concentration obtained after sonication of the suspensions using the Bradford method. To measure the activity of BtMinpp, 5 µL of vesicle suspension (corresponding to 5 µg of soluble protein) was added to 70 µL of either Tris buffer (25mM, pH 7.5) or in glycine-HCl buffer (20 mM, pH 2.5) and the PiColorLock Gold Phosphate Detection System was used. For *ex vivo* $InsP_6$ degradation experiments, caecal contents from 4 C57BL/6 mice were homogenised in distilled water,

centrifuged at 10,000 g for 5 min and the pellet was washed once with distilled water. Supernatants at each washing step were collected and the volume was adjusted to a final dilution factor of 1:10 (wt:v). To remove microorganisms, the supernatants were filtered through a 0.22 µm PES membrane (Sartorius). The pH of the extracts was measured at 6.5. 10 µl of vesicle suspension was added to 90 µl of extract to which 1mM of $InsP_6$ was added. HPLC: Inositol phosphates were resolved by anion exchange chromatography on a Dionex CarboPac PA-200 column (3mm x 250mm) with guard column (3mm x 50mm) eluted at a flow rate of 0.4 $ml.min^{-1}$ with a gradient of methane sulfonic acid prepared from buffer reservoirs containing water (A) and 0.6M methane sulfonic acid (B) mixed in ratio: time (min), % B; 0,10; 22,60; 25,100; 38,100; 39,10; 49,10. Inositol phosphates were detected by absorbance measurement at 290nm after post-column addition at 0.2 ml. min^{-1}, of 0.1% (w/v) ferric nitrate in 2% (w/v) perchloric acid using a 375 µl knitted reaction coil.

References

Adams PD, Afonine PV, Bunkoczi G, Chen VB, Davis IW, Echols N, Headd JJ, Hung LW, Kapral GJ, Grosse-Kunstleve RW, McCoy AJ, Moriarty NW, Oeffner R, Read RJ, Richardson DC, Richardson JS, Terwilliger TC, Zwart PH (2010) PHENIX: a comprehensive Python-based system for macromolecular structure solution. *Acta Crystallogr D* 66: 213-221

Ariza A, Moroz OV, Blagova EV, Turkenburg JP, Waterman J, Roberts SM, Vind J, Sjoholm C, Lassen SF, De Maria L, Glitsoe V, Skov LK, Wilson KS (2013) Degradation of phytate by the 6-phytase from Hafnia alvei: a combined structural and solution study. *PloS one* 8: e65062

Brearley CA, Hanke DE (1996) Metabolic evidence for the order of addition of individual phosphate esters to the myo-inositol moiety of inositol hexakisphosphate in the duckweed Spirodela polyrhiza L. *Biochem J* 314: 227-233

Caffrey JJ, Hidaka K, Matsuda M, Hirata M, Shears SB (1999) The human and rat forms of multiple inositol polyphosphate phosphatase: functional homology with a histidine acid phosphatase up-regulated during endochondral ossification. *Febs Lett* 442: 99-104

Chassard C, Delmas E, Lawson PA, Bernalier-Donadille A (2008) Bacteroides xylanisolvens sp nov., a xylan-degrading bacterium isolated from human faeces. *Int J Syst Evol Micr* 58: 1008-1013

Chen VB, Arendall WB, Headd JJ, Keedy DA, Immormino RM, Kapral GJ, Murray LW, Richardson JS, Richardson DC (2010) MolProbity: all-atom structure validation for macromolecular crystallography. *Acta Crystallogr D* 66: 12-21

Collaborative Computational Project N (1994) The CCP4 suite: Programs for protein crystallography. *Acta Crystallogr D Biol Crystallogr* 50: 760-763

Cowtan K (2006) The Buccaneer software for automated model building. 1. Tracing protein chains. *Acta Crystallogr D* 62: 1002-1011

Craxton A, Caffrey JJ, Burkhart W, Safrany ST, Shears SB (1997) Molecular cloning and expression of a rat hepatic multiple inositol polyphosphate phosphatase. *Biochem J* 328: 75-81

Dassa E, Boquet PL (1985) Identification of the Gene Appa for the Acid-Phosphatase (Ph Optimum 2.5) of Escherichia-Coli. *Mol Gen Genet* 200: 68-73

Ehrlich KC, Montalbano BG, Mullaney EJ, Dischinger HC, Jr., Ullah AH (1993) Identification and cloning of a second phytase gene (phyB) from Aspergillus niger (ficuum). *Biochemical and biophysical research communications* 195: 53-57

Emsley P, Cowtan K (2004) Coot: model-building tools for molecular graphics. *Acta Crystallogr D* 60: 2126-2132

Evans P (2006) Scaling and assessment of data quality. *Acta Crystallogr D Biol Crystallogr* 62: 72-82

Holm L, Park J (2000) DaliLite workbench for protein structure comparison. *Bioinformatics* 16: 566-567

Isbrandt LR, Oertel RP (1980) Conformational States of Myoinositol Hexakis(Phosphate) in Aqueous-Solution - a C-13 Nmr, P-31 Nmr, and Raman-Spectroscopic Investigation. *J Am Chem Soc* 102: 3144-3148

Jakob CG, Lewinski K, Kuciel R, Ostrowski W, Lebioda L (2000) Crystal structure of human prostatic acid phosphatase. *The Prostate* 42: 211-218

Kleywegt GJ, Henrick K, Dodson EJ, van Aalten DMF (2003) Pound-wise but penny-foolish-How well do macromolecules fare in macromolecular refinement? *Structure* 11: 1051-1059

Kostrewa D, Leitch FG, DArcy A, Broger C, Mitchell D, vanLoon APGM (1997) Crystal structure of phytase from Aspergillus ficuum at 2.5 angstrom resolution. *Nat Struct Biol* 4: 185-190

Kostrewa D, Wyss M, D'Arcy A, van Loon APGM (1999) Crystal structure of Aspergillus niger pH 2.5 acid phosphatase at 2.4 angstrom resolution. *J Mol Biol* 288: 965-974

Lee DC, Cottrill MA, Forsberg CW, Jia Z (2003) Functional insights revealed by the crystal structures of Escherichia coli glucose-1-phosphatase. *J Biol Chem* 278: 31412-31418

Leslie AG (1999) Integration of macromolecular diffraction data. *Acta Crystallogr D Biol Crystallogr* 55: 1696-1702

Lim D, Golovan S, Forsberg CW, Jia Z (2000) Crystal structures of Escherichia coli phytase and its complex with phytate. *Nat Struct Biol* 7: 108-113

Lindqvist Y, Schneider G, Vihko P (1993) Three-dimensional structure of rat acid phosphatase in complex with L(+)-tartrate. *J Biol Chem* 268: 20744-20746

Liu Q, Huang Q, Lei XG, Hao Q (2004) Crystallographic snapshots of Aspergillus fumigatus phytase, revealing its enzymatic dynamics. *Structure* 12: 1575-1583

Morris GM, Huey R, Lindstrom W, Sanner MF, Belew RK, Goodsell DS, Olson AJ (2009) AutoDock4 and AutoDockTools4: Automated Docking with Selective Receptor Flexibility. *J Comput Chem* 30: 2785-2791

Nagy R, Grob H, Weder B, Green P, Klein M, Frelet-Barrand A, Schjoerring JK, Brearley C, Martinoia E (2009) The Arabidopsis ATP-binding Cassette Protein AtMRP5/AtABCC5 Is a High Affinity Inositol Hexakisphosphate Transporter Involved in Guard Cell Signaling and Phytate Storage. *J Biol Chem* 284: 33614-33622

Noltmann E, Gubler CJ, Kuby SA (1961) Glucose 6-Phosphate Dehydrogenase (Zwischenferment) .1. Isolation of Crystalline Enzyme from Yeast. *J Biol Chem* 236: 1225-&

Notredame C, Higgins DG, Heringa J (2000) T-Coffee: A novel method for fast and accurate multiple sequence alignment. *J Mol Biol* 302: 205-217

Oakley AJ (2010) The structure of *Aspergillus nige*r phytase PhyA in complex with a phytate mimetic. *Biochem Biophys Res Comm* 397: 745-749

Osborn MJ, Gander JE, Parisi E, Carson J (1972) Mechanism of Assembly of Outer Membrane of Salmonella-Typhimurium - Isolation and Characterization of Cytoplasmic and Outer Membrane. *J Biol Chem* 247: 3962-&

Ragon M, Hoh F, Aumelas A, Chiche L, Moulin G, Boze H (2009) Structure of Debaryomyces castellii CBS 2923 phytase. *Acta Crystallogr F* 65: 321-326

Rigden DJ (2008) The histidine phosphatase superfamily: structure and function. *Biochem J* 409: 333-348

Rodriguez E, Han YM, Lei XG (1999) Cloning, sequencing, and expression of an Escherichia coli acid phosphatase/phytase gene (appA2) isolated from pig colon. *Biochemical and biophysical research communications* 257: 117-123

Romano PR, Wang J, O'Keefe RJ, Puzas JE, Rosier RN, Reynolds PR (1998) HiPER1, a phosphatase of the endoplasmic reticulum with a role in chondrocyte maturation. *J Cell Sci* 111: 803-813

Sambrook J, Russel DW (2001) *Molecular cloning: A laboratory Manual*, New York, USA: Cold Spring Harbor Press.

Sheldrick GM (2008) SHELXL97 and SHELXS97, programs for crystal structure solution and refinement. *Acta Crystallogr* 64: 112-122

Shoemaker NB, Getty C, Gardner JF, Salyers AA (1986) Tn4351 Transposes in Bacteroides Spp and Mediates the Integration of Plasmid R751 into the Bacteroides Chromosome. *J Bacteriol* 165: 929-936

Singh H, Felts RL, Schuermann JP, Reilly TJ, Tanner JJ (2009) Crystal Structures of the histidine acid phosphatase from Francisella tularensis provide insight into substrate recognition. *J Mol Biol* 394: 893-904

Smith CJ, Rollins LA, Parker AC (1995) Nucleotide sequence determination and genetic analysis of the Bacteroides plasmid, pBI143. *Plasmid* 34: 211-222

Tancula E, Feldhaus MJ, Bedzyk LA, Salyers AA (1992) Location and Characterization of Genes Involved in Binding of Starch to the Surface of Bacteroides-Thetaiotaomicron. *J Bacteriol* 174: 5609-5616

Trott O, Olson AJ (2010) Software News and Update AutoDock Vina: Improving the Speed and Accuracy of Docking with a New Scoring Function, Efficient Optimization, and Multithreading. *J Comput Chem* 31: 455-461

Ullah AH, Dischinger HC, Jr. (1993) Aspergillus ficuum phytase: complete primary structure elucidation by chemical sequencing. *Biochemical and biophysical research communications* 192: 747-753

van Hartingsveldt W, van Zeijl CM, Harteveld GM, Gouka RJ, Suykerbuyk ME, Luiten RG, van Paridon PA, Selten GC, Veenstra AE, van Gorcom RF, et al. (1993) Cloning, characterization and overexpression of the phytase-encoding gene (phyA) of Aspergillus niger. *Gene* 127: 87-94

Wegmann U, Horn N, Carding SR (2013) Defining the Bacteroides Ribosomal Binding Site. *Appl Environ Microb* 79: 1980-1989

Figure S1. The products of BtMinpp activity against $InsP_6$ are distinct from the products of attack of fungal phytase. Phytase and Minpps were incubated with *myo*-inositol(1,[^{32}P]2,3,4,5,6)P_6 and the products resolved on a Partisphere SAX HPLC column eluted with a gradient of $(NH_4)_2HPO_4$. human MINPP1 (A), *Aspergillus ficuum* phytase (Sigma) (B), recombinant *B. thetaiotaomicron* BtMinpp (C). The traces also show the elution of internal standards of AMP, ADP and ATP monitored at a wavelength of 259nm (grey line), and radioactivity monitored on-line by Cerenkov counting (black line). The regions of the chromatogram in which $InsP_2$, $InsP_3$ and $InsP_4$ isomers elute are indicated in (A). The Ins(1,[^{32}P]2,3,4,5,6)P_6 preparation used in (A) contained residual [^{32}P]ATP.

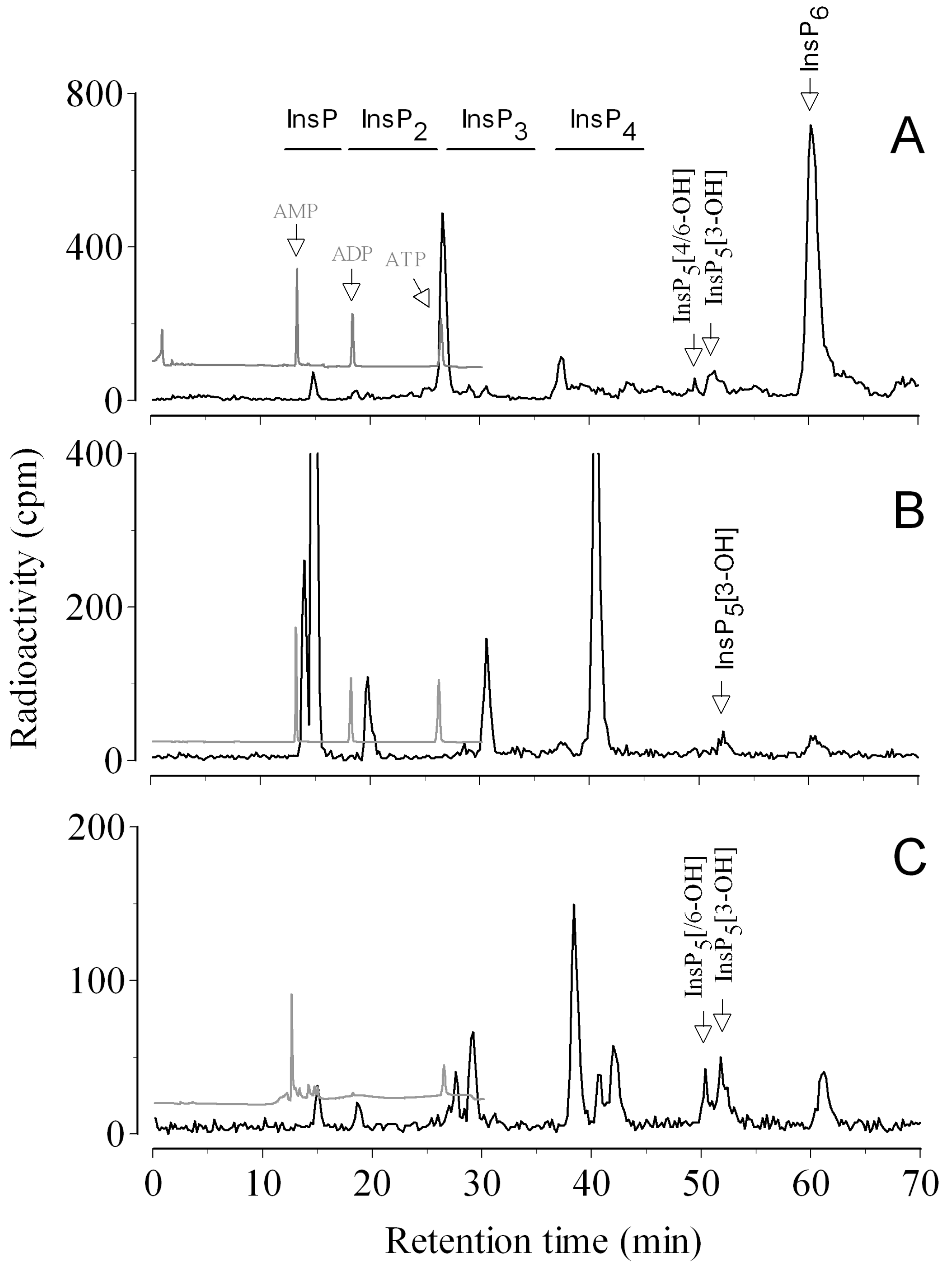

Figure S2. Products of hydrolysis of Ins(1,3,4,5)P_4 and $InsP_6$. (A) Native BtMinpp was incubated with [3H]Ins(1,3,4,5)P_4 at pH 7.0. The products were mixed with [14C]Ins(1,3,4)P_3 and resolved by HPLC. Fractions were collected and radioactivity estimated by scintillation counting: 3H, open circles; 14C, filled circles. The position of elution of Ins(1,4,5)P_3, revealed in parallel HPLC runs, is also shown. (B) Native BtMinpp was incubated with *myo*-inositol(1,[32P]2,3,4,5,6)P_6. Products were resolved by Partisphere SAX HPLC and detected by on-line Cerenkov counting. The regions of the chromatogram in which $InsP_3$ and $InsP_4$ isomers elute are indicated. The peak eluting at approximately 15 min was present in the preparation of *myo*-inositol(1,[32P]2,3,4,5,6)P_6.

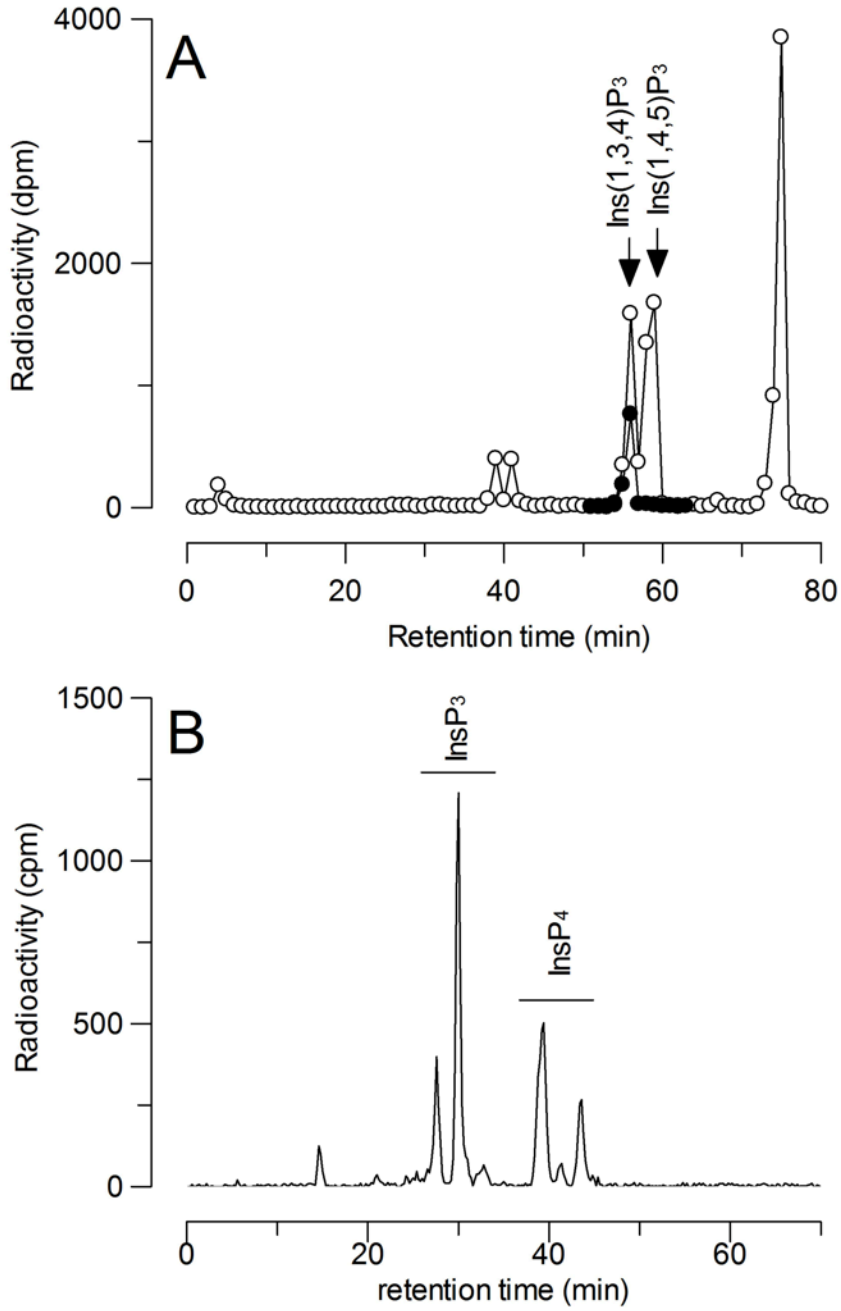

Figure S3. Relative $InsP_6$ phosphatase activity (%) of recombinant BtMinpp at different pH values

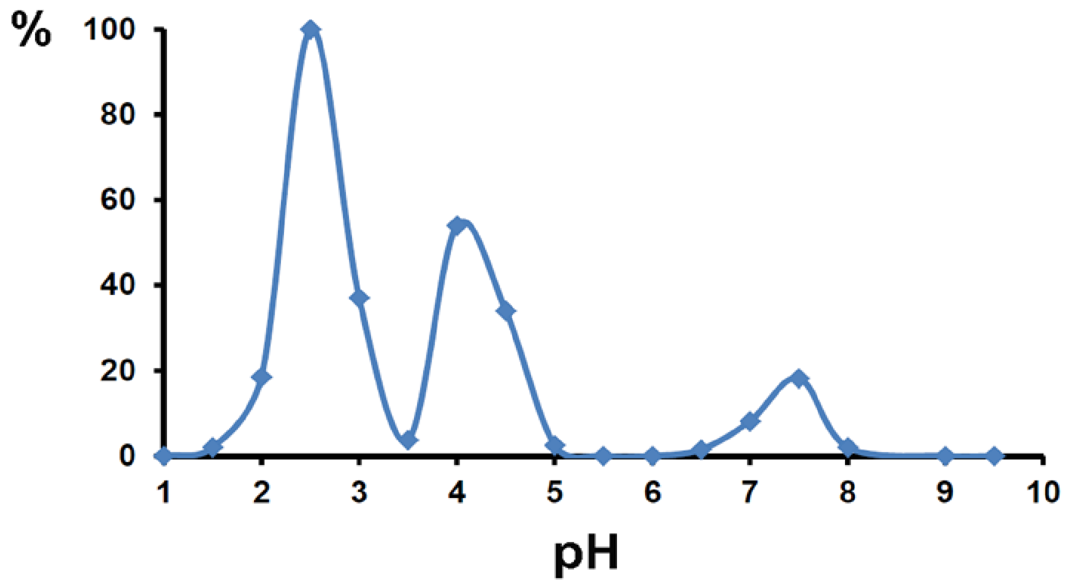

Figure S4. Active site electron density for the complex of BtMinpp with inorganic phosphate. A region of the Sigma-A weighted double difference Fourier electron density map contoured at 1.1 σ for the residues of the fingerprint RHGXRXP (58-64) and HAE (323-325) residues of the active site of the complex of BtMinpp with inorganic phosphate. Active site residues and phosphate group are shown as sticks.

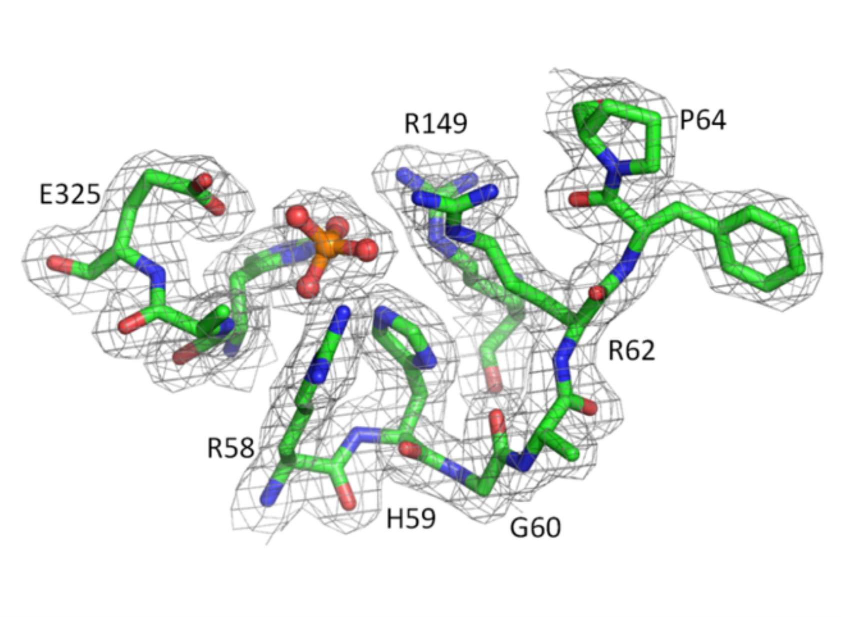

Figure S5. A T-coffee (Notredame et al, 2000) alignment of the sequences of BtMinpp and selected MINPPs. Sequences are: BtMinpp – this study, sequence database entry Q89YI8; Human – Human multiple inositol polyphosphate histidine phosphatase 1, Q9UNW1; Rat - Rat multiple inositol polyphosphate histidine phosphatase 1 O35217; Chicken - Histidine phosphatase of the endoplasmic reticulum from chicken, Q92170. Residue numbering follows that of BtMinpp. Absolutely conserved residues are shown in red boxes, conserved residues are boxed in blue. Secondary structural elements of BtMinpp are shown, labelled to reflect homology with the corresponding elements in the structure of *A. niger* PhyA (Oakley, 2010). Blue triangles indicate residues which make direct contact with the $InsS_6$ ligand in its complex with BtMinpp, whilst green triangles show those additional residues which have an atom within 8 Å of the ligand.

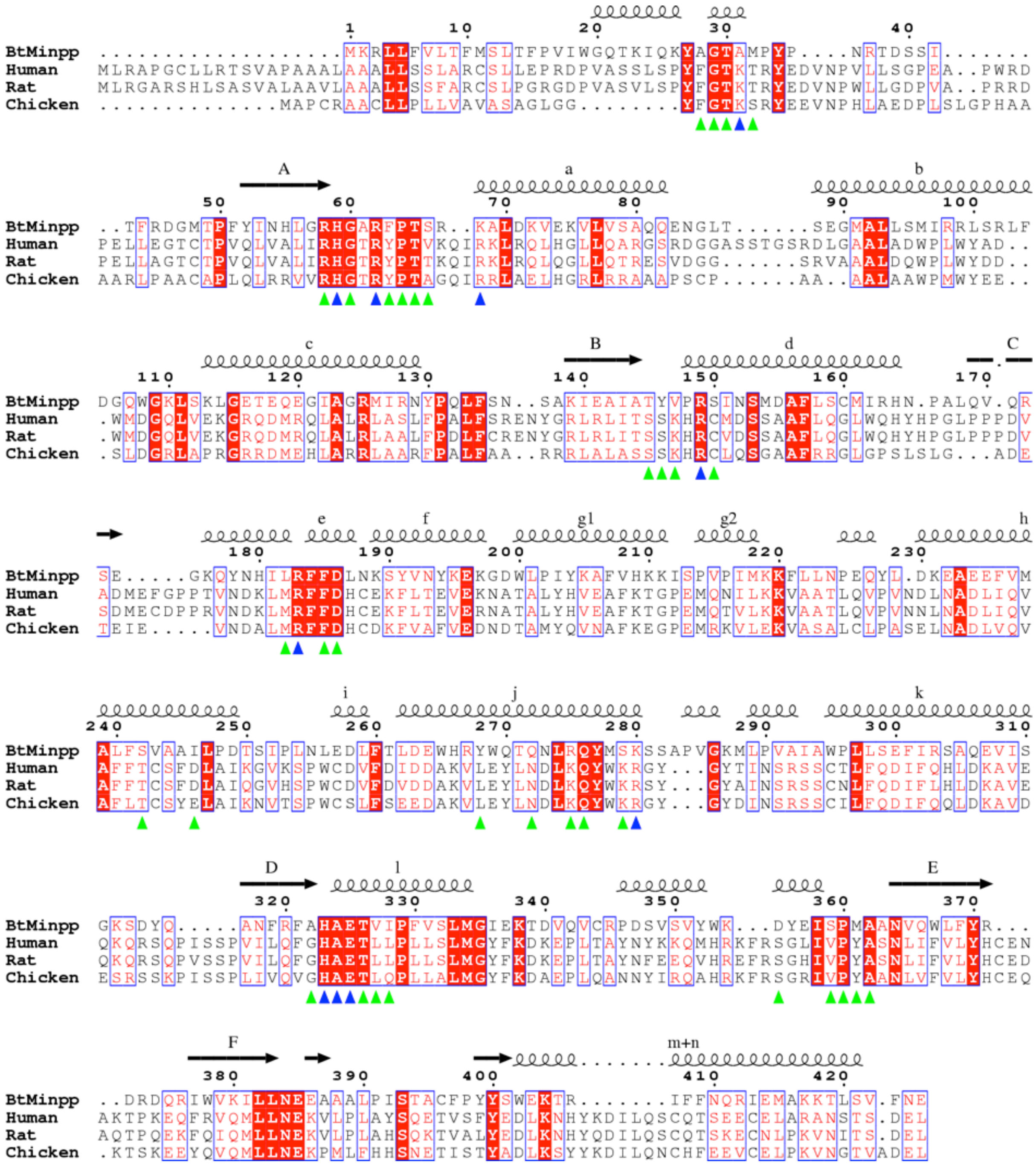

BtMinpp ...................MKRLLFVLTFMSLTFPVIWGQTKIQKYAGTAMPYP....NRTDSSI.......
Human MLRAPGCLLRTSVAPAAALAAALLSSLARCSLLEPRDPVASSLSPYFGTKTRYEDVNPVLLSGPEA..PWRD
Rat MLRGARSHLSASVALAAVLAAALLSSFARCSLPGRGDPVASVLSPYFGTKTRYEDVNPWLLGDPVA..PRRD
Chicken .............MAPCRAACLLPLLVAVASAGLGG........YFGTKSRYEEVNPHLAEDPLSLGPHAA
A
a
b
BtMinpp ..TFRDGMTPFYINHLGRHGARFPTSR..KALDKVEKVLVSAQQENGLT......SEGMALLSMIRRLSRLF
Human PELLEGTCTPVQLVALIRHGTRYPTVKQIRKLRQLHGLLQARGSRDGGASSTGSRDLGAALADWPLWYAD..
Rat PELLAGTCTPVQLVALIRHGTRYPTTKQIRKLRQLQGLLQTRESVDGG......SRVAAALDQWPLWYDD..
Chicken AARLPAACAPLQLRRVVRHGTRYPTAGQIRRLAELHGRLRRAAAPSCP......AA..AALAAWPMWYEE..
c
B
d
C
BtMinpp DGQWGKLSKLGETEQEGIAGRMIRNYPQLFSN..SAKIEAIATYVPRSINSMDAFLSCMIRHN.PALQV.QR
Human .WMDGQLVEKGRQDMRQLALRLASLFPALFSRENYGRLRLITSSKHRCMDSSAAFLQGLWQHYHPGLPPDV
Rat .WMDGQLVEKGRQDMRQLALRLAALFPDLFCRENYGRLRLITSSKHRCVDSSAAFLQGLWQHYHPGLPPDV
Chicken .SLDGRLAPRGRRDMEHLARRLAARFPALFAA..RRRLALASSSKHRCLQSGAAFRRGLGPSLSLG...ADE
e
f
g1
g2
h
BtMinpp SE.....GKQYNHILRFFDLNKSYVNYKEKGDWLPIYKAFVHKKISPVPIMKKFLLNPEQYL.DKEAEEFVM
Human ADMEFGPPTVNDKLMRFFDHCEKFLTEVEKNATALYHVEAFKTGPEMQNILKKVAATLQVPVNDLNADLIQV
Rat SDMECDPPRVNDKLMRFFDHCEKFLTEVERNATALYHVEAFKTGPEMQTVLKKVAATLQVPVNNLNADLIQV
Chicken TEIE.....VNDALMRFFDHCDKFVAFVEDNDTAMYQVNAFKEGPEMRKVLEKVASALCLPASELNADLVQV
i
j
k
BtMinpp ALFSVAAILPDTSIPLNLEDLFTLDEWHRYWQTQNLRQYMSKSSAPVGKMLPVAIAWPLLSEFIRSAQEVIS
Human AFFTCSFDLAIKGVKSPWCDVFDIDDAKVLEYLNDLKQYWKRGY...GYTINSRSSCTLFQDIFQHLDKAVE
Rat AFFTCSFDLAIQGVHSPWCDVFDVDDAKVLEYLNDLKQYWKRSY...GYAINSRSSCNLFQDIFLHLDKAVE
Chicken AFLTCSYELAIKNVTSPWCSLFSEEDAKVLEYLNDLKQYWKRGY...GYDINSRSSCILFQDIFQQLDKAVD
D
l
E
BtMinpp GKSDYQ.....ANFRFAHAETVIPFVSLMGIEKTDVQVCRPDSVSVYWK...DYEISPMAANVQWLFYR...
Human QKQRSQPISSPVILQFGHAETLLPLLSLMGYFKDKEPLTAYNYKKQMHRKFRSGLIVPYASNLIFVLYHCEN
Rat QKQRSQPVSSPVILQFGHAETLLPLLSLMGYFKDKEPLTAYNFEEQVHREFRSGHIVPYASNLIFVLYHCED
Chicken ESRSSKPISSPLIVQVGHAETLQPLLALMGYFKDAEPLQANNYIRQAHRKFRSGRIVPYAANLVFVLYHCEQ
F
m+n
BtMinpp ..DRDQRIWVKILLNEEAAALPISTACFPYYSWEKTR.......IFFNQRIEMAKKTLSV.FNE
Human AKTPKEQFRVQMLLNEKVLPLAYSQETVSFYEDLKNHYKDILQSCQTSEECELARANSTS.DEL
Rat AQTPQEKFQIQMLLNEKVLPLAHSQKTVALYEDLKNHYQDILQSCQTSKECNLPKVNITS.DEL
Chicken .KTSKEEYQVQMLLNEKPMLFHHSNETISTYADLKSYYKDILQNCHFEEVCELPKVNGTVADEL

Table S1 Alignment of BtMinpp protein sequence with known MINPPs and phytases*

Accession	Description	Coverage (%)	E value	Identity	Alignment scores
NP_813655	*B. thetaiotaomicron* BtMinpp	100	0	425/425 (100%)	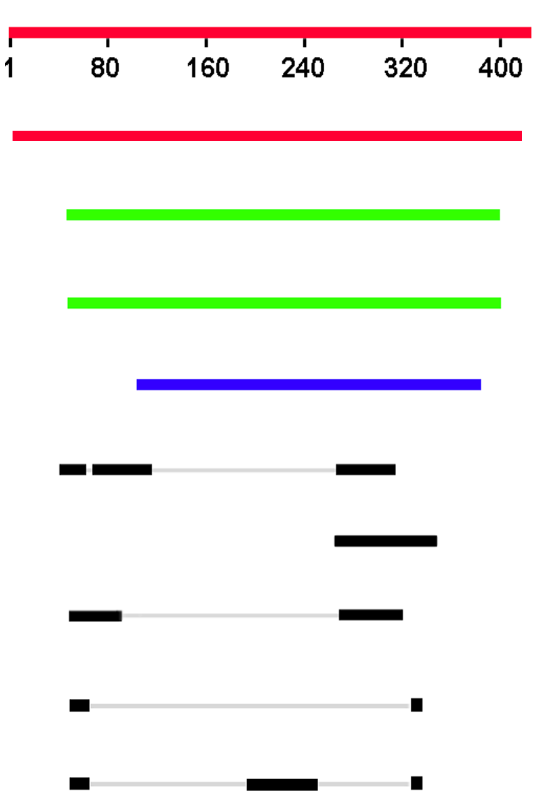
ZP_03475410	*Parabacteroides johnsonii* PRABACTJOHN_01069	97	0	257/416 (62%)	
NP_004888	Human MINPP1 (Caffrey et al, 1999)	82	2e-14	90/381 (23%)	
NP_062136	Rat MINPP1 (Craxton et al, 1997)	82	6e-15	81/377 (21%)	
NP_989975	Chicken HiPER1 (Romano et al, 1998)	65	5e-08	70/306 (23%)	
CAA78904	*Aspergillus niger* PhyA (van Hartingsveldt et al, 1993)	28	4e-05	32/83 (38%)	
AAA02934	*A. niger* PhyB (Ehrlich et al, 1993)	18	6e-04	21/84 (25%)	
AAB26466	*A. ficuum* phytase (Ullah & Dischinger, 1993)	19	2e-05	32/83 (38%)	
AAA72086	*Escherichia coli* AppA (Dassa & Boquet, 1985)	5	0.27	7/15 (47%)	
AAR87658	*E. Coli* AppA2 (Rodriguez et al, 1999)	17	0.18	21/70 (30%)	

*Similarities of BtMinpp to known proteins were determined using the BLASTP 2 sequences tool for local alignment. BtMinpp is shown on the top line of the Table so that its entire length can be displayed for comparative purposes. The colour key for the alignment scores (in bits) is: red, ≥ 200; green, 50-80; blue, 40-50; black, 18-40.

Table S2. Molecular and Enzymatic Properties of BtMinpp

Molecular mass	49 kDa, monomer
pH optimum	2.5, 4.0 and 7.5 (see Figure S1)
pH stability	Stable for 24 hours at pH between 2 and 5 at 4°C
Kinetic constants at pH 7.5 and 37°C	$\underline{InsP_6}$ K_M : 44.78 ± µM<br>V_{max} : 69.63 µmoles/min/mg<br>K_{cat} : 57.1 s^{-1}<br>$\underline{InsP_4}$ $K_{M\,:}$ 19.4 ± 8.9 µM<br>$V_{max\,:}$ 1423 ± 24 µmoles/min/mg<br>K_{cat} : 1167 s^{-1}
Kinetic constants at pH 2.5 and 37°C	$\underline{InsP_6}$ K_M : 18.4 ± 4.1 µM<br>V_{max} : 178 ± 32 µmoles/min/mg<br>K_{cat} : 146 s^{-1}<br>$\underline{InsP_4}$ K_M : 27.1 ± 7.2 µM<br>V_{max} : 6385 ± 732 µmoles/min/mg<br>K_{cat} : 5238 s^{-1}
Pepsin resistance	High; >95% of initial activity after 30min at 37°C with 3000U pepsin
Enzyme activity at low pH	pH 2.0: 18% of initial activity<br>pH 1.0: 1.6% of initial activity
Temperature optimum	55°C
Residual activity at high temperatures	70°C, 10 min: 1.26% of initial activity<br>80°C, 10 min: 0% of initial activity

Table S3. Statistics from the comparison of BtMinpp with the structures of branch 2 histidine phosphatases. The residue identity is calculated as the percentage of residues found to be structurally-equivalent and chemically identical in the alignment.

Protein	PDB entry	Uniprot entry	Z-score	Number of Equivalent residues	Residue identity (%)	RMSD (Å)
AfPhyA	1SK8	O00092	35.0	366	15	2.7
AnPhyA	3K4Q	P34752	34.5	360	16	2.5
AnPhyB	1QFX	P34755	32.6	362	14	3.2
DcPhyt	2GFI	A2TBB4	33.1	363	18	2.7
EcAppA	1DKL	P07102	23.9	308	13	4.1
EcG1Pase	1NT4	P19926	24.4	306	12	3.8
FtHAP	3IT1	O57942	25.1	291	14	3.6
HaPhyA	4ARO	H9TUK5	23.6	306	12	4.2
HsPAP	1CVI	P15309	25.5	300	13	3.6
RnPAP	1RPA	P20646	25.1	300	14	3.6

Table S4. Predicted low energy binding modes resulting from *in silico* docking of $InsP_6$ to enzyme structures. Columns show the identities of the phosphate groups of $InsP_6$ bound at either the S3 or S2 specificity subsites of BtMinpp and PhyA (*A.niger* phytase, PDB entry 3K4Q). The binding modes are indicated as either obverse (O) or reverse (R).

**Enzyme**	**S3 pocket**	**S2 pocket**	**Binding Mode**
BtMinpp	D-5	D-6	R
	D-6	D-1	R
	D-3	D-2	O
	D-6	D-5	O
PhyA	D-3	D-2	O

Table S5. Genes annotated as phytases in 341 microbial genomes isolated from the human gastrointestinal tract (from the Human Microbiome Project, http://www.hmpdacc-resources.org/cgi-bin/imgm_hmp/main.cgi)

Genome	Phylum	Locus Tag	Gene Product Name
Acidaminococcus sp. D21	Firmicutes	ACDG_00696	phytase
Oxalobacter formigenes OXCC13	Proteobacteria	OFBG_00876	4-phytase
Paenibacillus sp. HGF7	Firmicutes	HMPREF9413_5666	3-phytase
Providencia stuartii ATCC 25827	Proteobacteria	PstuA_020100004501	4-phytase

Table S6. Source organisms for MINPP proteins*

Common name	NCBI Reference Sequence	Scientific name
Acidovorax avenae	YP_004236708.1	*Acidovorax avenae subsp. avenae* ATCC 19860
Acinetobacter baumannii	YP_001846348.1	*Acinetobacter baumannii* ACICU
Acinetobacter calcoaceticus	ADY81607.1	*Acinetobacter calcoaceticus* PHEA-2
Acinetobacter sp. DR1	YP_003732360.1	*Acinetobacter sp.* DR1
Aeromonas veronii	YP_004393571.1	*Aeromonas veronii* B565
Bacteroides thetaiotaomicron	NP_813655.1	*Bacteroides thetaiotaomicron* VPI-5482
Bacteroides xylanisolvens	CBK68912.1	*Bacteroides xylanisolvens* XB1A
Bacteroides xylanisolvens	CBK66891.1	*Bacteroides xylanisolvens* XB1A
Bacteroides xylanisolvens	CBK68541.1	*Bacteroides xylanisolvens* XB1A
Bifidobacterium dentium	YP_003361382.1	*Bifidobacterium dentium* Bd1
Bifidobacterium longum	YP_002321769.1	*Bifidobacterium longum subsp. infantis* ATCC 15697
Bifidobacterium pseudocatenulatum	ZP_03743199.1	*Bifidobacterium pseudocatenulatum* DSM 20438
Bradyrhizobium sp. BTAi1	YP_001236730.1	*Bradyrhizobium sp. BTAi1*
Burkholderia ambifaria	ZP_02888054.1	*Burkholderia ambifaria* IOP40-10
Burkholderia cenocepacia	ZP_04942680.1	*Burkholderia cenocepacia* PC184
Burkholderia pseudomallei	ZP_01765847.1	*Burkholderia pseudomallei* 305
Burkholderia thailandensis	ZP_02466008.1	*Burkholderia thailandensis* MSMB43
Clavibacter michiganensis	YP_001221200.1	*Clavibacter michiganensis* subsp. *michiganensis* NCPPB 382
Enhydrobacter aerosaccus	ZP_05619011.1	*Enhydrobacter aerosaccus* SK60
Fusobacterium mortiferum	ZP_04568756.1	*Fusobacterium mortiferum* ATCC 9817
Marinomonas sp. MWYL1	YP_001339041.1	*Marinomonas sp.* MWYL1
Parabacteroides johnsonii	ZP_03475410.1	*Parabacteroides johnsonii* DSM 18315
Photobacterium leiognathi	ZP_08311993.1	*Photobacterium leiognathi* subsp. *mandapamensis* svers.1.1.
Rhodococcus erythropolis	YP_002763531.1	*Rhodococcus erythropolis* PR4
Spirochaeta smaragdinae	YP_003803564.1	*Spirochaeta smaragdinae* DSM 11293
Streptomyces hygroscopicus	ZP_07292879.1	*Streptomyces hygroscopicus* ATCC 53653
Variovorax paradoxus	YP_004156191.1	*Variovorax paradoxus* EPS
Arabidopsis thaliana	NP_563856.1	*Arabidopsis thaliana*
Barley	ABJ98329.1	*Hordeum vulgare*
Lily	ABD96177.1	*Lilium longiflorum*
Maize	NP_001149246.1	*Zea mays*
Physcomitrella patens	XP_001765102.1	*Physcomitrella patens subsp. patens*
Rice Indica	EAY92352.1	*Oryza sativa Indica Group*
Rice Japonica	NP_001051705.1	*Oryza sativa Japonica Group*
Selaginella moellendorffii	XP_002985961.1	*Selaginella moellendorffii*
Sorghum	XP_002466207.1	*Sorghum bicolor*
Wheat	ABJ98331.1	*Triticum aestivum*
Albugo laibachii	CCA15134.1	*Albugo laibachii Nc14*
Capsaspora owczarzaki	EFW47484.1	*Capsaspora owczarzaki ATCC 30864*
Dictyostelium discoideum	XP_638245.1	*Dictyostelium discoideum AX4*
Phytophthora infestans	XP_002907675.1	*Phytophthora infestans T30-4*
Chimpanzee	XP_507896.2	*Pan troglodytes*

Common marmoset	XP_003087945.1	*Callithrix jacchus*
Giant panda	XP_002756410.1	*Ailuropoda melanoleuca*
Gray short-tailed opossum	XP_001374318.1	*Monodelphis domestica*
Horse	XP_001503270.1	*Equus caballus*
Human	AAD02437.1	*Homo sapiens*
Macaque	XP_001101346.1	*Macaca mulatta*
Mouse	EDL41725.1	*Mus musculus*
Oikopleura dioica	CBY40869.1	*Oikopleura dioica*
Oxen	NP_001033664.2	*Bos taurus*
Rat	NP_062136.1	*Rattus norvegicus*
Sumatran orangutan	NP_001126173.1	*Pongo abelii*
Vase tunicate	XP_002121164.1	*Ciona intestinalis*
Western clawed frog	XP_002935472.1	*Xenopus (Silurana) tropicalis*
Wild boar	XP_001927707.1	*Sus scrofa*

*Animals, blue; Plants, green; Protists, salmon-pink; Bacteria, yellow.

Table S7. *Escherichia coli* and *Bacteroides* strains used in this study

Species	Strain	Relevant characteristics*	Source or reference
E. coli	HB101(pRK2013)	$Kana^r$, Contains RK2 transfer genes	DMSZ Collection
	GC10	Cloning strain	Sigma
	GC10(pGH051)	Tet^R	This work
	BL21-CodonPlus (DE3) RIL	Cm^R	Stratagene
	Rosetta™ 2(DE3)pLysS	Cm^R	Novagen
	B834 (DE3)		Novagen
	B834-CodonPlus (DE3) RIL	Cm^R, B834 (DE3) transformed with RIL plasmid	This work
	GH24	Amp^R Cm^R, *E. coli* BL21 (DE3) codon+ with pGH07	This work
	GH25	Amp^R Cm^R, *E. coli* B834 (DE3) codon+ with pGH07	This work
B. fragilis	NCTC 9343		DMSZ Collection
B. xylanisolvens	XB1A		(Chassard et al, 2008)
B. thetaiotomicron	VPI-5482		DMSZ Collection
B. thetaiotomicron	GH59	VPI-5482 with tetracycline marked minpp, Tet^r	This work
B. thetaiotomicron	GH120	GH59 containing pGH038 plasmid, Tet^r, Cc^r, expressing low levels of BtMinpp	This work
B. thetaiotomicron	GH115	GH59 containing pGH037 plasmid, Tet^r, Cc^r, expressing high levels of BtMinpp	This work

* Kana, kanamycin; Tet, tetracycline; Cm, chloramphenicol, Amp, ampicillin; Cc, clindamycin
